# Leveraging Internet Search Data to Improve the Prediction and Prevention of Noncommunicable Diseases: Retrospective Observational Study

**DOI:** 10.2196/18998

**Published:** 2020-11-12

**Authors:** Chenjie Xu, Zhi Cao, Hongxi Yang, Ying Gao, Li Sun, Yabing Hou, Xinxi Cao, Peng Jia, Yaogang Wang

**Affiliations:** 1 School of Public Health Tianjin Medical University Tianjin China; 2 Department of Epidemiology and Health Statistics School of Public Health Zhejiang University School of Medicine Hangzhou China; 3 School of Public Health Yale University New Haven, CT United States; 4 Health Management Center Tianjin Medical University General Hospital Tianjin China; 5 School of Nursing Tianjin Medical University Tianjin China; 6 Department of Land Surveying and Geo-Informatics The Hong Kong Polytechnic University Hong Kong China; 7 International Institute of Spatial Lifecourse Epidemiology Hong Kong China

**Keywords:** noncommunicable diseases, internet searches, Google Trends, infodemiology, infoveillance, early warning model, United States

## Abstract

**Background:**

As human society enters an era of vast and easily accessible social media, a growing number of people are exploiting the internet to search and exchange medical information. Because internet search data could reflect population interest in particular health topics, they provide a new way of understanding health concerns regarding noncommunicable diseases (NCDs) and the role they play in their prevention.

**Objective:**

We aimed to explore the association of internet search data for NCDs with published disease incidence and mortality rates in the United States and to grasp the health concerns toward NCDs.

**Methods:**

We tracked NCDs by examining the correlations among the incidence rates, mortality rates, and internet searches in the United States from 2004 to 2017, and we established forecast models based on the relationship between the disease rates and internet searches.

**Results:**

Incidence and mortality rates of 29 diseases in the United States were statistically significantly correlated with the relative search volumes (RSVs) of their search terms (*P*<.05). From the perspective of the goodness of fit of the multiple regression prediction models, the results were closest to 1 for diabetes mellitus, stroke, atrial fibrillation and flutter, Hodgkin lymphoma, and testicular cancer; the coefficients of determination of their linear regression models for predicting incidence were 80%, 88%, 96%, 80%, and 78%, respectively. Meanwhile, the coefficient of determination of their linear regression models for predicting mortality was 82%, 62%, 94%, 78%, and 62%, respectively.

**Conclusions:**

An advanced understanding of search behaviors could augment traditional epidemiologic surveillance and could be used as a reference to aid in disease prediction and prevention.

## Introduction

One of the Sustainable Development Goals (SDGs) set by the United Nations General Assembly in 2015 was to reduce premature mortality from noncommunicable diseases (NCDs) by one-third by 2030 [1]. According to the World Health Statistics 2019 [2], NCDs have collectively caused 41 million deaths worldwide, equivalent to 71% of all global deaths. The majority of those deaths were caused by the following NCDs: cardiovascular disease (CVD), cancer, and diabetes. According to the statistics of the Global Burden of Disease (GBD) in 2017 [3], diabetes was the most common NCD in the United States and ischemic heart disease ranked second. In 2020, 1,806,590 new cancer cases and 606,520 cancer deaths are projected to occur in the United States [4].

In the era of social media, there is a current trend in the population for individuals to search the internet for information before they consult with specialists for recommendations [5-7]. Various social media and online communities that enable connectivity have unprecedented influence. They are expanding their reach into the health care domain [8-12]. Researchers have shown that patients with cancer, diabetes, and other chronic conditions search online before and after diagnosis [13]. According to a report by the American Community Survey [14], the percentage of households with a computer has increased almost tenfold since 1984. Like computer use, the percentage of households using the internet has also increased over time [14]. Google accounts for the vast majority of the US search engine market, reaching more than 80% of the population [15].

The application of search engine data to the field of disease surveillance is a classic case of big data application. When GFT was first launched [16], it attracted widespread attention and was followed by many scholars. Previous studies have mainly utilized Google to study outbreaks of influenza epidemics [17-19]. Other studies have tried to mine social network data (such as Facebook and Twitter) through text mining to identify patients’ concerns about drugs, examine their feelings, and understand public opinion [20]. Due to the lack of real-time monitoring data of NCDs, few studies had used internet data to predict the trends of NCDs [21,22]. Our study is the first to explore a correlation among these many different types of online NCD searches with disease prevalence in the United States. We hypothesized that internet search behaviors could reflect people’s awareness of NCDs, and thus the disease characteristics of NCDs (such as incidence and mortality rates) would correlate strongly with the internet search frequency. It also provides a new information channel for grasping public health concerns and promoting NCD prevention.

## Methods

### Disease Data

We obtained the national incidence and mortality rates for NCDs in the United States from the GBD database for 14 years (from 2004 to 2017) [3]. In this study, we initially selected 31 types of NCDs, namely diabetes mellitus, ischemic heart disease, stroke, atrial fibrillation and flutter, prostate cancer, breast cancer, lung cancer, colon and rectal cancer, malignant skin melanoma, non-Hodgkin lymphoma, uterine cancer, cardiomyopathy and myocarditis, kidney cancer, pancreatic cancer, bladder cancer, leukemia, liver cancer, stomach cancer, lip and oral cavity cancer, brain and nervous system cancer, thyroid cancer, multiple myeloma, ovarian cancer, cervical cancer, esophageal cancer, larynx cancer, gallbladder and biliary tract cancer, Hodgkin lymphoma, testicular cancer, mesothelioma, and hypertensive heart disease.

### Internet Search Data

Because internet search data are updated in real time, this study mainly considered monthly internet searches from the Google Trends website from 2004 to 2018 at the national level [23]. We downloaded monthly relative search volumes for each search term of each disease. The search data were downloaded from Google Trends in December 2019.

### Selection of Search Terms

Different kinds of NCDs have different search terms and each disease has a core search term. We determined the core search term based on the disease name in the GBD database. Google Trends has the function of “related searches” [24]: after entering the core search term, other search terms related to this term in the “related searches” section can be seen at the bottom of the page, which contains up to 49 related search terms and sentences. This method determines the approximate scope of a search term selection based on the object to be studied, which can avoid the subjectivity of search term selection in the research to a certain extent and can minimize the omission of core terms. After the primary selection of terms, the next step was the filtering of search terms. Three types of terms were generally filtered out in this study. The first type was terms with meanings that had nothing to do with the research object. After the primary selection, some terms were still irrelevant to the research object, even if a phrase included the object to be studied, likely because some words have multiple meanings. The second type of terms to filter out was those with a small search volume. Some of the included terms had zero search volume within the specified time frame. Because this study focused on time differences, it was required that search terms had a high search frequency throughout the entire period.

Specific search terms are listed in Multimedia Appendix 1. The selected terms were not searched in quotes. Each data point represents the relative search volumes (RSVs) of specific query terms on a normalized scale of 0 to 100. The RSVs were divided by the total searches of the particular geographic location and a particular time range it represents to compare the relative popularity of the query terms. For example, compared with the total search volumes, if a particular region had a higher number of specific query terms, its RSV would be closer to 100. Data of internet searches used in this study are publicly available, anonymous, and cannot be tracked back to identifiable individuals.

### Statistical Analysis

We identified search terms for each NCD based on the above criteria. We performed the Pearson correlation analysis to evaluate the relationship between the known incidence and mortality rates of the NCDs and the RSVs to filter search terms continuously. Finally, the terms that have no significant correlation with the research object were also deleted in the subsequent analysis.

We also considered dealing with the multicollinearity of the RSVs of the search terms. Multicollinearity refers to the distortion or inaccuracy of model estimation due to the high correlation between explanatory variables in a linear regression model. In this study, we calculated the correlation coefficients between the RSVs of the search terms of each disease.

Based on the above steps, we established multiple linear regression models for each disease, and the RSVs of multiple search terms were used for prediction and analysis.

The general form of the multiple linear regression model can be expressed as follows:

y = β_0_ + β_1_x_2_ + β_2_x_2_ + ... + β*_K_*x*_k_* + ε,

where β_0_, β_1_, β_2_,..., β*_K_* is the parameter of the model and ε is the error term. The error term reflects the influence of random factors on y, which cannot be determined by the variability explained by the linear relationship between x*_k_* and y. In this study, we established two regression models for each NCD, one based on the correlation between RSVs and incidence rate and the other based on the correlation between RSVs and the mortality rate.

Statistical analysis was conducted using IBM SPSS software (version 22.0), and Stata (version 15; StataCorp LLC). The statistical significance was set as *P*<0.05 (two-sided test).

## Results

### Correlation Analysis

Table 1 and Multimedia Appendix 2 display the correlation coefficients between the incidence rates and the RSVs of all of the selected search terms for the NCDs. They also display the correlation coefficients between the mortality rates and the RSVs. We found statistically significant correlations between the rates and the RSVs, especially for five diseases with high incidence rates: diabetes mellitus, stroke, atrial fibrillation and flutter, Hodgkin lymphoma, and testicular cancer. For Hodgkin lymphoma, the RSV of each search term was negatively correlated with the incidence rate. For some diseases, we did not find statistically significant correlations between the RSVs and the mortality and incidence rates; these search terms were excluded from the subsequent analysis. If the correlations between the rates and RSVs of all search terms of a disease were found to be not statistically significant, the disease would be excluded from the analysis. For example, prostate cancer and hypertensive heart disease were excluded from further analysis because the RSVs of all of their search terms did not correlate with incidence and mortality rates at the same time.

The RSVs of the specific search terms were correlated with the incidence rates for all of the NCDs, with *P* values less than .05 (Multimedia Appendix 3). In addition, the RSVs of the specific search terms were correlated with the mortality rates for all of the NCDs. Multimedia Appendix 4 displays the cross correlation analysis results among search terms.

**Table 1:** Correlation coefficients between the incidence and mortality rates of diabetes mellitus, stroke, atrial fibrillation and flutter, Hodgkin lymphoma, and testicular cancer and their relative search volumes.

		Incidence rate		Mortality rate		
		*R*_incidence_	*P* value	*R*_mortality_	*P* value	
**Search terms for diabetes mellitus**						
	What is diabetes mellitus type 2	0.648	<.001	–0.636	<.001	
	What is type 2 diabetes	0.746	<.001	–0.789	<.001	
	Causes of diabetes mellitus	–0.295	<.001	0.250	.001	
	Signs of diabetes	0.866	<.001	–0.900	.001	
	What is type 1 diabetes	0.729	<.001	–0.756	<.001	
	Hypoglycemia	–0.522	<.001	0.403	<.001	
	Hyperlipidemia	0.686	<.001	–0.757	<.001	
**Search terms for stroke**						
	Signs of stroke in women	0.893	<.001	–0.495	<.001	
	Symptoms of stroke in women	0.609	<.001	–0.663	<.001	
	Signs of a stroke in women	0.906	<.001	–0.447	<.001	
	Stroke symptoms in men	0.805	<.001	–0.655	<.001	
	Minor stroke	0.245	.001	0.164	.03	
	Symptoms of mini stroke	0.558	<.001	–0.223	.004	
	Signs of stroke in men	0.887	<.001	–0.392	<.001	
	Signs of mini stroke	0.733	<.001	–0.277	.003	
	What are the signs of a stroke	0.745	<.001	–0.381	<.001	
**Search terms for atrial fibrillation and flutter**						
	Atrial fibrillation	0.163	.03	0.200	.009	
	Afib^a^	0.953	<.001	0.936	<.001	
	Atrial fibrillation with rvr^b^	0.218	.004	0.209	.007	
	Atrial fibrillation and stroke	–0.350	<.001	–0.359	<.001	
	Ablation of atrial fibrillation	–0.224	.003	–0.233	.002	
	Heart flutter	0.880	<.001	0.878	<.001	
	Atrial fibrillation vs flutter	0.363	<.001	0.376	<.001	
	Signs of atrial fibrillation	0.202	.009	0.205	.008	
	Atrial flutter vs atrial fibrillation	0.230	.003	0.230	.002	
	Atrial flutter	0.389	<.001	0.378	<.001	
	Atrial flutter ecg^c^	0.179	.02	0.161	.04	
	Atrial flutter ekg^d^	–0.163	.03	–0.165	.03	
	Atrial flutter vs fibrillation	0.391	<.001	0.403	<.001	
	Atrial fibrillation ecg	0.435	<.001	0.430	<.001	
	What is atrial fibrillation	0.685	<.001	0.681	<.001	
	a fib^e^	0.965	<.001	0.957	<.001	
	Treatment of atrial flutter	–0.187	.015	–0.185	.02	
	What causes atrial flutter	0.328	<.001	0.331	<.001	
**Search terms for Hodgkin lymphoma**						
	Hodgkin lymphoma	–0.518	<.001	–0.404	<.001	
	Hodgkin lymphoma cancer	–0.407	<.001	–0.316	<.001	
	Hodgkin lymphoma symptoms	–0.496	<.001	–0.413	<.001	
	Lymphoma symptoms	–0.365	<.001	–0.524	<.001	
	What is lymphoma	–0.773	<.001	–0.688	<.001	
	What is Hodgkin lymphoma	–0.635	<.001	–0.561	<.001	
	Hodgkins	0.823	<.001	0.801	<.001	
	Symptoms of Hodgkin lymphoma	–0.416	<.001	–0.346	<.001	
	Symptoms of lymphoma	–0.534	<.001	–0.590	<.001	
	Hodgkin lymphoma survival rate	–0.425	<.001	–0.357	<.001	
	Hodgkin lymphoma vs non Hodgkin lymphoma	–0.513	<.001	–0.432	<.001	
	Hodgkin vs non Hodgkin	–0.547	<.001	–0.452	<.001	
	Hodgkin lymphoma prognosis	–0.227	.003	–0.201	.009	
	B cell lymphoma	–0.303	<.001	–0.233	.002	
	B cell non odgkin lymphoma	–0.538	<.001	–0.493	<.001	
	Hodgkin lymphoma causes	–0.433	<.001	–0.363	<.001	
	Stage 4 lymphoma	–0.416	<.001	–0.366	<.001	
	Classical Hodgkin lymphoma	–0.495	<.001	–0.490	<.001	
	Hodgkin lymphoma stage 4	–0.415	<.001	–0.344	<.001	
**Search terms for testicular cancer**						
	Testicular cancer	–0.821	<.001	0.742	<.001	
	Causes of testicular cancer	–0.292	<.001	0.280	<.001	
	Symptoms for testicular cancer	–0.196	.01	0.248	.001	
	Testicular cancer ribbon	0.342	<.001	–0.275	<.001	
	Testicular cancer prognosis	–0.366	<.001	0.368	<.001	
	Testicular cancer risk factors	–0.185	.02	0.217	.005	
	What does testicular cancer look like	0.317	<.001	–0.254	.001	
	How do you get testicular cancer	0.320	<.001	–0.214	.005	
	Does testicular cancer spread	0.297	<.001	–0.217	.005	
	What are signs of testicular cancer	0.557	<.001	–0.476	<.001	

^a^afib: atrial fibrillation.

^b^rvr: rapid ventricular response.

^c^ecg: electrocardiogram.

^d^ekg: electrocardiogram.

^e^a fib: atrial fibrillation.

### Trends in Internet Searches, Incidence Rates, and Mortality Rates

**Figure 1:**
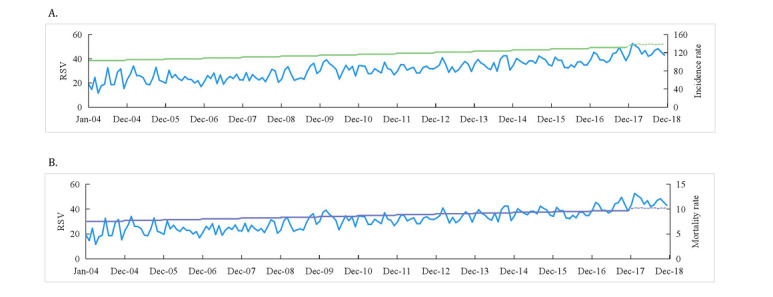
Trends of atrial fibrillation and flutter from 2004 to 2018. (A) Trends of incidence rate and relative search volume (RSV) of atrial fibrillation and flutter from 2004 to 2018. (B) Trends of mortality rate and RSV of atrial fibrillation and flutter from 2004 to 2018. The blue line represents the RSV of each noncommunicable disease from 2004 to 2018, the green line represents the incidence rates, the purple line represents the mortality rates, and the dotted line represents the forecast for morbidity and mortality in 2018.

Figure 1 shows a time series of the RSVs, incidence rates, and mortality rates for atrial fibrillation and flutter from 2004 to 2018. Trends of other diseases are displayed in Multimedia Appendix 5. Based on the correlation analysis, we predicted the incidence and mortality rates in 2018. As can be seen in the figure, the incidence rates of most diseases and RSVs fit well. A similar pattern was observed between RSVs and mortality rates. The predicted incidence and mortality rates varied with the fluctuations in the RSVs for most NCDs.

### Multiple Linear Regression Models

**Figure 2:**
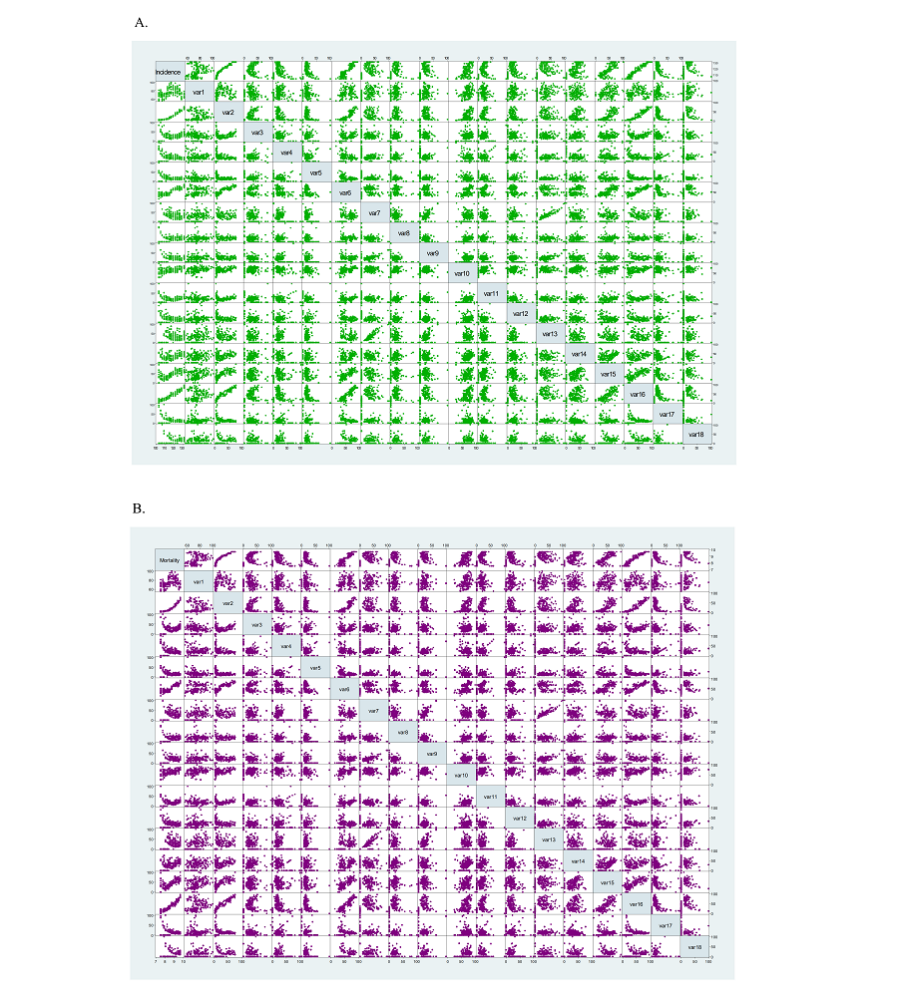
(A) Scatter plot of relative search volumes and incidence rate of atrial fibrillation and flutter in the United States. (B) Scatter plot of relative search volumes and mortality rate of atrial fibrillation and flutter in the United States.

Based on the correlations, two prediction models were established for each disease, namely the incidence prediction model and the mortality prediction model. Figure 2 shows the relationship between the independent variable (RSV for each search term) and the dependent variable (incidence and mortality rates of atrial fibrillation and flutter) in the model. The relationship between the independent variable and the dependent variable of all of the NCDs can be seen in Multimedia Appendix 6. The prediction models are shown in Multimedia Appendix 7. Table 2 shows the degree of fit of multiple linear regression models. The results of all of the NCDs are displayed in Multimedia Appendix 8. For diabetes mellitus, stroke, atrial fibrillation and flutter, Hodgkin lymphoma, and testicular cancer, the coefficient of determination of the linear regression models for predicting incidence was 80%, 88%, 96%, 80%, and 78%, respectively.

Meanwhile, the coefficient of determination of the linear regression models for predicting mortality was 82%, 62%, 94%, 78%, and 62%, respectively. From the perspective of the goodness of fit of the multiple regression prediction models of other NCDs, most of the results were close to 1.

**Table 2:** Evaluation results of prediction models.

	Incidence rate			Mortality rate		
	*R²*	Adjusted *R²*	RMSE^a^	*R²*	Adjusted *R²*	RMSE
Diabetes mellitus	80%	80%	20.54	82%	81%	0.59
Stroke	88%	87%	1.43	62%	60%	1.15
Atrial fibrillation and flutter	96%	95%	1.91	94%	94%	0.17
Hodgkin lymphoma	80%	77%	0.10	78%	75%	0.01
Testicular cancer	78%	77%	0.01	62%	60%	0.00

^a^Root-mean-square error.

## Discussion

### Principal Findings

In recent years, using internet search data to detect influenza has been a research hotspot. Most of the studies utilized data sources from Google Trends or Google Flu Trends [25-27]. Although the existing literature has conducted empirical research on the correlation between search data and influenza, there is generally a lack of systematic preprocessing methods for NCDs. This study mainly focused on three types of diseases: diabetes, cancer, and CVDs. We found that the frequency of searches correlated strongly with previously reported disease epidemiology.

The choice of search terms was a key feature of this study. Based on the disease names used by the GBD, we used the “related searches” function of the Google search engine to supplement the search term and thus obtain a more comprehensive primary selection at a low cost. In terms of the search term selection, compared with Pearson correlation analysis conducted in the previous research, the use of cross-correlation analysis can also determine the time relationship between search terms and disease occurrence and determine the leading search terms in time so as to build a predictive model. The above preprocessing methods provided a better data foundation for the establishment of early warning models.

This study demonstrated the feasibility of combining historical information with search information for the early warning of NCDs, laying the foundation for future model optimization. As the most recent data publicly available from the Centers for Disease Control and Prevention is at least 2 years old while Google Trends data are available nearly instantaneously, this resource could potentially provide a more timely and cost-effective data source for public health researchers [28]. Thus, real-time internet searches could be particularly useful. Studies have shown that tracking and monitoring search behaviors, as well as text mining on social media, can provide new ways to study public concerns about NCDs and information-seeking behaviors [29-32]. Researchers have realized that people can search, understand, and evaluate health information on internet resources and that they harness the information they receive to address health problems [33]. At present, it is necessary to enhance the efficiency of prevention and auxiliary diagnosis for patients with NCDs or the general population by online information transmission. The collection of real-time relevant search data from search engines provides a new way to prevention and control NCDs.

Most types of NCDs that we examined showed statistically significant correlations, although prostate cancer and hypertensive heart disease did not. This pattern can be attributable to different reasons. First, NCDs are highly prevalent. As people are paying more attention to these diseases, the self-management consciousness of patients with certain NCDs has been improving. Second, the monthly rates of diagnosis of NCDs are missing. Third, this pattern could be partly influenced by active public health campaigns, which may broadly increase search volume regardless of disease metrics. Between 2007 and 2014, the incidence of cancer in men in the United States declined rapidly, and then remained stable until 2016, which was related to changes in screening strategies for colon, rectal, and prostate cancer [4]. Doctors and scientists in the United States have noticed that previous prostate-specific antigen (PSA)-based screenings may lead to an overdiagnosis for prostate cancer and have reduced the use of PSA for prostate cancer screening. A previous study showed that fewer older adults in the United States are having heart attacks, and among those who do have them, more are surviving from them [34]. The country has made continuous efforts to prevent heart attacks and improve patient care. Health insurance agencies, the American Heart Association, and other research organizations—as well as a large number of researchers, clinicians, and public health experts—are committed to reducing risk by promoting a healthy lifestyle. At the same time, the recommendations for the secondary prevention of heart disease are more common and standardized. The extensive development of angiography and other related technologies, and the deepening of people’s understanding of CVDs, will affect the frequency of retrieval of related diseases on the internet. This trend can also be seen in our prediction model. The incidence of lung cancer in the United States has also continued to decline due to the continuous implementation of antismoking activities and effective control of the smoking rate. Fourth, we extracted internet search data from as early as 2004. In the past, individuals with the highest risk of NCDs often had limited access to the internet. To date, the internet has ushered in great changes. The number of internet users is currently growing at a rate of more than 11 new users per second, bringing the total number of new users per day to an astonishing 1 million. More and more people tend to search for health-related information online. Our results also indicated that the coefficient of determination and adjusted coefficient of determination of the regression models for diabetes, stroke, atrial fibrillation and flutter, Hodgkin lymphoma, and testicular cancer were higher than for the other diseases studied, indicating that the regression models are better fit to the incidence and mortality rates of these NCDs. This might mean that online search behaviors and volumes can help health professionals to conduct near real-time monitoring of NCDs.

Although the method of using internet search data to predict influenza has made great progress in real time, it still lacks in accuracy. Since 2011, GFT has been overestimating the number of influenza-like illnesses (ILIs), especially the forecast of the peak season of influenza. In 2013, the forecast deviation was even as high as 140%, which may indicate that there is a certain gap in the forecast based on internet data alone. In the traditional methods of influenza prediction, although the manually collected ILI monitoring samples are lagging behind, they are more accurate because of rigorous scientific experiments. Therefore, internet data cannot completely replace the traditional data collection methods but should be used as a complement to the latter.

### Limitations

Our study has some unavoidable limitations. First, because the data from internet searches are public and anonymous, we could not determine who conducted search activities in this study, and the limited number of search terms could not fully represent the search preferences of all people. Second, the use of Google Trends cannot be fully representative of the overall population, since only individuals with access to the internet can be accounted for. Third, we could only obtain the annual incidence and mortality data for NCDs, but not the data with smaller granularity, which might have affected the accuracy of the prediction model. Fourth, as the search algorithm of Google Trends is dynamic, we could not retrieve the original RSVs, and the RSVs of the same search term obtained in different time periods were different. Fifth, the spatiotemporal data of search engines have many limitations, such as high noise and uncertainty. We hope to find ways to identify and reduce bias in search engine data before we utilize web-based data to provide useful information.

### Public Health Implications

With the widespread use of internet searches, our study found a correlation between the RSVs and the incidence and mortality rates of the NCDs. This indicates that the search engine data can be used in the early warning and prevention of NCDs, such as diabetes, cancer, and CVDs. We should make good use of such data, especially when the traditional registry data are insufficient or unavailable.

## Appendix

Multimedia Appendix 1 Search terms of all the noncommunicable diseases.

Multimedia Appendix 2 Correlation analysis among the relative search volumes, incidence rates, and mortality rates.

Multimedia Appendix 3 Correlation coefficients among internet searches, incidence rates, and mortality rates in the United States (contains search terms that were ultimately included in the study).

Multimedia Appendix 4 Cross-correlation analysis results among search terms.

Multimedia Appendix 5 Trends of all the noncommunicable diseases.

Multimedia Appendix 6 Relationship between the independent variable and the dependent variable of all the noncommunicable diseases.

Multimedia Appendix 7 Prediction models.

Multimedia Appendix 8 Evaluation results of prediction models for all the chronic diseases.

## Abbreviations

CVD: cardiovascular disease
GBD: Global Burden of Disease
GFT: Google Flu Trends
ILIs: influenza-like illnesses
NCDs: noncommunicable diseases
PSA: prostate-specific antigen
RSV: relative search volume
SDGs: Sustainable Development Goals
