# Supplementary material for: Leveraging Internet Search Data to Improve the Prediction and Prevention of Noncommunicable Diseases: Retrospective Observational Study
Source: J Med Internet Res. 2020 Nov 12;22(11):e18998. doi: 10.2196/18998 (PMC7691086; doi:10.2196/18998)
Supplement: Multimedia Appendix 3 [file jmir_v22i11e18998_app3.doc]

| **Multimedia Appendix 3. Correlation coefficients among internet searches, incidence and mortality rates in the US (Contains search terms that were ultimately included in the study)** | | | | | |
| --- | --- | --- | --- | --- | --- |
|  | **Search Term** | **Incidence** | | **Mortality** | |
|  |  | Rincidence | **P** | Rmortality | **P** |
| 1 | what is diabetes mellitus type 2 | 0.648 | <0.001 | -0.636 | <0.001 |
| 2 | what is type 2 diabetes | 0.746 | <0.001 | -0.789 | <0.001 |
| 3 | causes of diabetes mellitus | -0.295 | <0.001 | 0.25 | 0.001 |
| 4 | signs of diabetes | 0.866 | <0.001 | -0.9 | 0.001 |
| 5 | what is type 1 diabetes | 0.729 | <0.001 | -0.756 | <0.001 |
| 6 | hypoglycemia | -0.522 | <0.001 | 0.403 | <0.001 |
| 7 | hyperlipidemia | 0.686 | <0.001 | -0.757 | <0.001 |
|  | **Search Term** | **Incidence** | | **Mortality** | |
|  |  | Rincidence | **P** | Rmortality | **P** |
| 1 | ischemic heart disease | -0.433 | <0.001 | -0.468 | <0.001 |
| 2 | what is Ischemic heart disease | -0.28 | <0.001 | -0.29 | <0.001 |
| 3 | coronary heart disease | 0.335 | <0.001 | 0.336 | <0.001 |
| 4 | agent orange ischemic heart disease | -0.389 | <0.001 | -0.421 | <0.001 |
| 5 | ischemic stroke | -0.421 | <0.001 | -0.422 | <0.001 |
| 6 | cad | 0.536 | <0.001 | 0.602 | <0.001 |
| 7 | ischemic heart disease is most accurately defined as | -0.365 | <0.001 | -0.4 | <0.001 |
| 8 | angina pectoris | -0.468 | <0.001 | -0.481 | <0.001 |
|  | **Search Term** | **Incidence** | | **Mortality** | |
|  |  | Rincidence | **P** | Rmortality | **P** |
| 1 | signs of stroke in women | 0.893 | <0.001 | -0.495 | <0.001 |
| 2 | symptoms of stroke in women | 0.609 | <0.001 | -0.663 | <0.001 |
| 3 | signs of a stroke in women | 0.906 | <0.001 | -0.447 | <0.001 |
| 4 | stroke symptoms in men | 0.805 | <0.001 | -0.655 | <0.001 |
| 5 | minor stroke | 0.245 | 0.001 | 0.164 | 0.034 |
| 6 | symptoms of mini stroke | 0.558 | <0.001 | -0.223 | 0.004 |
| 7 | signs of stroke in men | 0.887 | <0.001 | -0.392 | <0.001 |
| 8 | signs of mini stroke | 0.733 | <0.001 | -0.277 | 0.003 |
| 9 | what are the signs of a stroke | 0.745 | <0.001 | -0.381 | <0.001 |
|  | **Search Term** | **Incidence** | | **Mortality** | |
|  |  | Rincidence | **P** | Rmortality | **P** |
| 1 | atrial fibrillation | 0.163 | 0.034 | 0.2 | 0.009 |
| 2 | afib | 0.953 | <0.001 | 0.936 | <0.001 |
| 3 | atrial fibrillation with rvr | 0.218 | 0.004 | 0.209 | 0.007 |
| 4 | atrial fibrillation and stroke | -0.35 | <0.001 | -0.359 | <0.001 |
| 5 | ablation of atrial fibrillation | -0.224 | 0.003 | -0.233 | 0.002 |
| 6 | heart flutter | 0.88 | <0.001 | 0.878 | <0.001 |
| 7 | atrial fibrillation vs flutter | 0.363 | <0.001 | 0.376 | <0.001 |
| 8 | signs of atrial fibrillation | 0.202 | 0.009 | 0.205 | 0.008 |
| 9 | atrial flutter vs atrial fibrillation | 0.23 | 0.003 | 0.23 | 0.002 |
| 10 | atrial flutter | 0.389 | <0.001 | 0.378 | <0.001 |
| 11 | atrial flutter ecg | 0.179 | 0.02 | 0.161 | 0.038 |
| 12 | atrial flutter ekg | -0.163 | 0.034 | -0.165 | 0.032 |
| 13 | atrial flutter vs fibrillation | 0.391 | <0.001 | 0.403 | <0.001 |
| 14 | atrial fibrillation ecg | 0.435 | <0.001 | 0.43 | <0.001 |
| 15 | what is atrial fibrillation | 0.685 | <0.001 | 0.681 | <0.001 |
| 16 | a fib | 0.965 | <0.001 | 0.957 | <0.001 |
| 17 | treatment of atrial flutter | -0.187 | 0.015 | -0.185 | 0.017 |
| 18 | what causes atrial flutter | 0.328 | <0.001 | 0.331 | <0.001 |
|  | **Search Term** | **Incidence** | | **Mortality** | |
|  |  | Rincidence | **P** | Rmortality | **P** |
| 1 | triple negative breast cancer | -0.188 | 0.015 | -0.555 | <0.001 |
| 2 | signs of breast cancer in women | -0.228 | 0.003 | -0.528 | <0.001 |
| 3 | inflammatory breast cancer symptoms | -0.154 | 0.046 | -0.171 | 0.027 |
| 4 | how do you get breast cancer | -0.212 | 0.006 | -0.383 | <0.001 |
| 5 | what month is breast cancer awareness | -0.164 | 0.034 | -0.306 | <0.001 |
| 6 | what month is breast cancer awareness month | -0.177 | 0.021 | -0.32 | <0.001 |
| 7 | is breast cancer painful | -0.205 | 0.008 | -0.432 | <0.001 |
|  | **Search Term** | **Incidence** | | **Mortality** | |
|  |  | Rincidence | **P** | Rmortality | **P** |
| 1 | early signs of lung cance | 0.216 | 0.005 | 0.175 | 0.024 |
| 2 | lung cancer symptoms in women | -0.355 | <0.001 | -0.597 | <0.001 |
| 3 | symptoms of lung cancer in women | -0.279 | <0.001 | -0.475 | <0.001 |
| 4 | stage 4 lung cancer survival rate | -0.195 | 0.011 | -0.4 | <0.001 |
| 5 | lung cancer symptoms in men | -0.331 | <0.001 | -0.608 | <0.001 |
| 6 | early symptoms of lung cancer | 0.204 | 0.008 | 0.267 | <0.001 |
| 7 | what are signs of lung cancer | -0.234 | 0.002 | -0.28 | <0.001 |
| 8 | keytruda lung cancer | 0.518 | <0.001 | 0.205 | 0.008 |
| 9 | lung cancer | 0.407 | <0.001 | 0.627 | <0.001 |
|  | **Search Term** | **Incidence** | | **Mortality** | |
|  |  | Rincidence | **P** | Rmortality | **P** |
| 1 | colon cancer symptoms | -0.164 | 0.033 | -0.408 | <0.001 |
| 2 | symptoms of colon cancer in women | -0.164 | 0.034 | -0.591 | <0.001 |
| 3 | colon cancer signs in women | 0.164 | 0.034 | -0.367 | <0.001 |
| 4 | signs of colon cancer in women | 0.187 | 0.015 | -0.349 | <0.001 |
| 5 | signs for colon cancer | 0.221 | 0.004 | 0.186 | 0.016 |
| 6 | signs of colon cancer in men | 0.183 | 0.018 | -0.382 | <0.001 |
| 7 | colon cleanse | -0.389 | <0.001 | -0.331 | <0.001 |
| 8 | stage 4 colon cancer life expectancy | 0.165 | 0.032 | -0.26 | 0.001 |
|  | **Search Term** | **Incidence** | | **Mortality** | |
|  |  | Rincidence | **P** | Rmortality | **P** |
| 1 | malignant skin melanoma | -0.317 | <0.001 | -0.291 | <0.001 |
| 2 | basal cell carcinoma | 0.646 | <0.001 | 0.629 | <0.001 |
| 3 | skin cancer types | 0.49 | <0.001 | 0.468 | <0.001 |
|  | **Search Term** | **Incidence** | | **Mortality** | |
|  |  | Rincidence | **P** | Rmortality | **P** |
| 1 | non hodgkin lymphoma | 0.601 | <0.001 | 0.272 | <0.001 |
| 2 | non hodgkins lymphoma | -0.16 | 0.038 | 0.232 | 0.003 |
|  | **Search Term** | **Incidence** | | **Mortality** | |
|  |  | Rincidence | **P** | Rmortality | **P** |
| 1 | uterine cancer | -0.21 | 0.009 | -0.164 | 0.034 |
| 2 | uterine polyps cancer | -0.386 | <0.001 | -0.378 | <0.001 |
| 3 | endometrial cancer symptoms | 0.251 | 0.001 | 0.223 | 0.004 |
| 4 | uterine cancer ribbon | 0.496 | <0.001 | 0.479 | <0.001 |
| 5 | uterine cancer diagnosis | -0.288 | <0.001 | -0.274 | <0.001 |
| 6 | uterine cancer survival rates | -0.232 | 0.002 | -0.228 | 0.003 |
| 7 | uterine fibroid symptoms | -0.219 | 0.004 | -0.189 | 0.014 |
| 8 | endometriosis symptoms | 0.354 | <0.001 | 0.256 | 0.001 |
| 9 | symptoms of uterine cancer | 0.359 | <0.001 | 0.321 | <0.001 |
|  | **Search Term** | **Incidence** | | **Mortality** | |
|  |  | Rincidence | **P** | Rmortality | **P** |
| 1 | acute cardiomyopathy | 0.195 | 0.011 | 0.183 | 0.017 |
| 2 | treatment for cardiomyopathy | 0.196 | 0.011 | 0.41 | <0.001 |
| 3 | myocarditis | 0.401 | <0.001 | 0.196 | 0.011 |
|  | **Search Term** | **Incidence** | | **Mortality** | |
|  |  | Rincidence | **P** | Rmortality | **P** |
| 1 | kidney cancer stage 4 | 0.67 | <0.001 | 0.669 | <0.001 |
| 2 | kidney cancer ribbon | 0.642 | <0.001 | 0.651 | <0.001 |
| 3 | kidney cancer life expectancy | 0.271 | <0.001 | 0.247 | 0.001 |
| 4 | kidney cancer metastasis | -0.258 | 0.001 | -0.248 | 0.001 |
| 5 | survival rate of kidney cancer | 0.193 | 0.012 | 0.165 | 0.032 |
|  | **Search Term** | **Incidence** | | **Mortality** | |
|  |  | Rincidence | **P** | Rmortality | **P** |
| 1 | pancreatic cancer | 0.263 | 0.001 | 0.23 | 0.003 |
| 2 | cause of pancreatic cancer | 0.424 | <0.001 | 0.427 | <0.001 |
| 3 | pancreatic cancer ribbon | 0.729 | <0.001 | 0.717 | <0.001 |
| 4 | test for pancreatic cancer | 0.516 | <0.001 | 0.502 | <0.001 |
| 5 | stage 3 pancreatic cancer | 0.626 | <0.001 | 0.624 | <0.001 |
| 6 | pancreatic cancer awareness | 0.283 | <0.001 | 0.272 | <0.001 |
| 7 | what cause pancreatic cancer | 0.428 | <0.001 | 0.428 | <0.001 |
| 8 | what are symptoms of pancreatic cancer | 0.287 | <0.001 | 0.269 | <0.001 |
| 9 | types of pancreatic cancer | -0.229 | 0.003 | -0.212 | 0.006 |
|  | **Search Term** | **Incidence** | | **Mortality** | |
|  |  | Rincidence | **P** | Rmortality | **P** |
| 1 | bladder cancer | -0.259 | 0.001 | -0.269 | <0.001 |
| 2 | treatment for bladder cancer | 0.182 | 0.018 | 0.172 | 0.026 |
| 3 | bladder cancer in men | 0.27 | <0.001 | 0.247 | 0.001 |
| 4 | treatment of bladder cancer | -0.176 | 0.023 | -0.176 | 0.022 |
| 5 | cause of bladder cancer | 0.26 | 0.001 | 0.263 | 0.001 |
| 6 | symptoms of bladder infection | 0.309 | <0.001 | 0.292 | <0.001 |
| 7 | bladder cancer stage 4 | 0.234 | 0.002 | 0.237 | 0.002 |
| 8 | symptoms of bladder cancer in women | 0.189 | 0.014 | 0.16 | 0.039 |
| 9 | bladder cancer symptoms in men | 0.301 | <0.001 | 0.275 | <0.001 |
| 10 | metastatic bladder cancer | -0.269 | <0.001 | -0.267 | <0.001 |
| 11 | cystoscopy | 0.694 | <0.001 | 0.696 | <0.001 |
| 12 | what are symptoms of bladder cancer | 0.161 | 0.038 | 0.165 | 0.033 |
|  | **Search Term** | **Incidence** | | **Mortality** | |
|  |  | Rincidence | **P** | Rmortality | **P** |
| 1 | acute leukemia prognosis | 0.198 | 0.01 | -0.216 | 0.005 |
| 2 | symptoms of leukemia | 0.303 | <0.001 | -0.372 | <0.001 |
| 3 | leukemia symptoms in women | 0.471 | <0.001 | -0.286 | <0.001 |
| 4 | signs of leukemia in children | 0.329 | <0.001 | -0.202 | 0.009 |
|  | **Search Term** | **Incidence** | | **Mortality** | |
|  |  | Rincidence | **P** | Rmortality | **P** |
| 1 | liver cancer | -0.635 | <0.001 | -0.606 | <0.001 |
| 2 | liver cancer ribbon | 0.644 | <0.001 | 0.653 | <0.001 |
| 3 | stage 4 liver cancer life expectancy | 0.43 | <0.001 | 0.412 | <0.001 |
| 4 | liver cancer symptoms in women | 0.323 | <0.001 | 0.291 | <0.001 |
| 5 | survival rate for liver cancer | 0.201 | 0.009 | 0.188 | 0.015 |
| 6 | stage 4 liver cancer survival rate | 0.182 | 0.018 | 0.175 | 0.023 |
| 7 | sign of liver cancer | 0.605 | <0.001 | 0.58 | <0.001 |
| 8 | stage four liver cancer | -0.255 | 0.001 | -0.251 | 0.001 |
|  | **Search Term** | **Incidence** | | **Mortality** | |
|  |  | Rincidence | **P** | Rmortality | **P** |
| 1 | stomach cancer | 0.179 | 0.02 | -0.248 | 0.001 |
| 2 | h pylori | -0.012 | <0.001 | -0.656 | <0.001 |
| 3 | treatment of stomach cancer | 0.204 | 0.008 | 0.355 | <0.001 |
| 4 | stomach cancer symptoms in women | -0.476 | <0.001 | -0.677 | <0.001 |
|  | **Search Term** | **Incidence** | | **Mortality** | |
|  |  | Rincidence | **P** | Rmortality | **P** |
| 1 | mouth cancer | 0.473 | <0.001 | 0.483 | <0.001 |
| 2 | cancer of the lip | -0.305 | <0.001 | -0.272 | <0.001 |
| 3 | symptoms of lip cancer | -0.212 | 0.006 | -0.198 | 0.01 |
| 4 | cancer in mouth | 0.76 | <0.001 | 0.71 | <0.001 |
|  | **Search Term** | **Incidence** | | **Mortality** | |
|  |  | Rincidence | **P** | Rmortality | **P** |
| 1 | brain cancer survival rate | 0.347 | <0.001 | 0.307 | <0.001 |
| 2 | cancer metastasis to brain | 0.287 | <0.001 | 0.284 | <0.001 |
| 3 | john mccain brain cancer | 0.219 | 0.004 | 0.218 | 0.004 |
| 4 | glioma brain cancer | -0.405 | <0.001 | -0.402 | <0.001 |
| 5 | pediatric brain cancer | -0.177 | 0.021 | -0.158 | 0.041 |
| 6 | brain cancer | 0.321 | <0.001 | 0.332 | <0.001 |
|  | **Search Term** | **Incidence** | | **Mortality** | |
|  |  | Rincidence | **P** | Rmortality | **P** |
| 1 | thyroid cancer | 0.589 | <0.001 | 0.59 | <0.001 |
| 2 | thyroid cancer survival rate | 0.482 | <0.001 | 0.488 | <0.001 |
| 3 | thyroid cancer ribbon | 0.684 | <0.001 | 0.708 | <0.001 |
| 4 | symptoms of thyroid problems | 0.555 | <0.001 | 0.45 | <0.001 |
| 5 | metastatic thyroid cancer | -0.411 | <0.001 | -0.382 | <0.001 |
| 6 | thyroid cancer symptoms in women | 0.344 | <0.001 | 0.201 | 0.009 |
| 7 | thyroid cancer awareness | 0.331 | <0.001 | 0.325 | <0.001 |
| 8 | stage 4 thyroid cancer | 0.472 | <0.001 | 0.493 | <0.001 |
| 9 | can thyroid cancer spread | 0.516 | <0.001 | 0.478 | <0.001 |
|  | **Search Term** | **Incidence** | | **Mortality** | |
|  |  | Rincidence | **P** | Rmortality | **P** |
| 1 | multiple myeloma | 0.169 | 0.028 | 0.21 | 0.006 |
| 2 | multiple myeloma stages | 0.354 | <0.001 | 0.355 | <0.001 |
| 3 | is multiple multiple myeloma cancer | 0.367 | <0.001 | 0.332 | <0.001 |
| 4 | what is multiple myeloma cancer | 0.227 | 0.003 | 0.195 | 0.011 |
| 5 | multiple melanoma | -0.541 | <0.001 | -0.438 | <0.001 |
| 6 | multiple myeloma treatments | -0.324 | <0.001 | 0.97 | <0.001 |
| 7 | bone marrow cancer | 0.174 | 0.024 | 0.236 | 0.002 |
| 8 | multiple myeloma ribbon | 0.604 | <0.001 | 0.547 | <0.001 |
|  | **Search Term** | **Incidence** | | **Mortality** | |
|  |  | Rincidence | **P** | Rmortality | **P** |
| 1 | ovarian cancer | 0.608 | <0.001 | 0.508 | <0.001 |
| 2 | ovarian cancer early symptoms | 0.308 | <0.001 | 0.297 | <0.001 |
| 3 | ovarian cancer metastasis | 0.322 | <0.001 | 0.283 | <0.001 |
| 4 | avastin | 0.604 | <0.001 | 0.418 | <0.001 |
| 5 | do I have ovarian cancer | -0.433 | <0.001 | -0.352 | <0.001 |
| 6 | what are signs of ovarian cancer | -0.488 | <0.001 | -0.448 | <0.001 |
| 7 | testing for ovarian cancer | 0.338 | <0.001 | 0.305 | <0.001 |
| 8 | screening for ovarian cancer | 0.212 | 0.006 | 0.171 | 0.027 |
| 9 | early symptoms of ovarian cancer | 0.365 | <0.001 | 0.318 | <0.001 |
| 10 | can ovarian cysts cause cancer | -0.263 | 0.001 | -0.231 | 0.003 |
|  | **Search Term** | **Incidence** | | **Mortality** | |
|  |  | Rincidence | **P** | Rmortality | **P** |
| 1 | symptoms of cervical cancer | -0.564 | <0.001 | 0.189 | 0.014 |
| 2 | what causes cervical cancer | -0.213 | 0.006 | 0.418 | <0.001 |
| 3 | what is hpv | 0.157 | 0.042 | 0.77 | <0.001 |
| 4 | how do you get cervical cancer | -0.256 | 0.001 | 0.263 | 0.001 |
| 5 | cervical cancer definition | 0.304 | <0.001 | 0.208 | 0.007 |
| 6 | cervical cancer staging | 0.168 | 0.03 | -0.462 | <0.001 |
|  | **Search Term** | **Incidence** | | **Mortality** | |
|  |  | Rincidence | **P** | Rmortality | **P** |
| 1 | esophageal cancer | 0.455 | <0.001 | 0.375 | <0.001 |
| 2 | symptoms of esophageal cancer | 0.454 | <0.001 | 0.393 | <0.001 |
| 3 | what is esophageal cancer | 0.298 | <0.001 | 0.289 | <0.001 |
| 4 | esophageal cancer signs | 0.424 | <0.001 | 0.406 | <0.001 |
| 5 | stage 4 esophageal cancer | 0.417 | <0.001 | 0.382 | <0.001 |
| 6 | signs of esophageal cancer | 0.495 | <0.001 | 0.479 | <0.001 |
| 7 | esophagus cancer symptoms | 0.251 | 0.001 | 0.245 | 0.001 |
| 8 | what causes esophageal cancer | 0.477 | <0.001 | 0.411 | <0.001 |
| 9 | esophageal tumor | -0.266 | 0.001 | -0.229 | 0.003 |
| 10 | esophageal cancer life expectancy | 0.188 | 0.015 | 0.164 | 0.034 |
|  | **Search Term** | **Incidence** | | **Mortality** | |
|  |  | Rincidence | **P** | Rmortality | **P** |
| 1 | larynx cancer | -0.231 | 0.003 | 0l071 | 0.363 |
| 2 | laryngectomy | 0.238 | 0.002 | 0.171 | 0.027 |
| 3 | cancer of larynx | -0.173 | 0.025 | 0.171 | 0.027 |
|  | **Search Term** | **Incidence** | | **Mortality** | |
|  |  | Rincidence | **P** | Rmortality | **P** |
| 1 | gallbladder pain symptoms | 0.698 | <0.001 | 0.254 | 0.001 |
| 2 | bile duct cancer | 0.564 | <0.001 | 0.425 | <0.001 |
|  | **Search Term** | **Incidence** | | **Mortality** | |
|  |  | Rincidence | **P** | Rmortality | **P** |
| 1 | Hodgkin lymphoma | -0.518 | <0.001 | -0.404 | <0.001 |
| 2 | hodgkin lymphoma cancer | -0.407 | <0.001 | -0.316 | <0.001 |
| 3 | hodgkin lymphoma symptoms | -0.496 | <0.001 | -0.413 | <0.001 |
| 4 | lymphoma symptoms | -0.365 | <0.001 | -0.524 | <0.001 |
| 5 | what is lymphoma | -0.773 | <0.001 | -0.688 | <0.001 |
| 6 | what is hodgkin lymphoma | -0.635 | <0.001 | -0.561 | <0.001 |
| 7 | hodgkins | 0.823 | <0.001 | 0.801 | <0.001 |
| 8 | symptoms of hodgkin lymphoma | -0.416 | <0.001 | -0.346 | <0.001 |
| 9 | symptoms of lymphoma | -0.534 | <0.001 | -0.59 | <0.001 |
| 10 | hodgkin lymphoma survival rate | -0.425 | <0.001 | -0.357 | <0.001 |
| 11 | hodgkin lymphoma vs non hodgkin lymphoma | -0.513 | <0.001 | -0.432 | <0.001 |
| 12 | hodgkin vs non hodgkin | -0.547 | <0.001 | -0.452 | <0.001 |
| 13 | hodgkin lymphoma prognosis | -0.227 | 0.003 | -0.201 | 0.009 |
| 14 | b cell lymphoma | -0.303 | <0.001 | -0.233 | 0.002 |
| 15 | b cell non hodgkin lymphoma | -0.538 | <0.001 | -0.493 | <0.001 |
| 16 | hodgkin lymphoma causes | -0.433 | <0.001 | -0.363 | <0.001 |
| 17 | stage 4 lymphoma | -0.416 | <0.001 | -0.366 | <0.001 |
| 18 | classical hodgkin lymphoma | -0.495 | <0.001 | -0.49 | <0.001 |
| 19 | hodgkin lymphoma stage 4 | -0.415 | <0.001 | -0.344 | <0.001 |
|  | **Search Term** | **Incidence** | | **Mortality** | |
|  |  | Rincidence | **P** | Rmortality | **P** |
| 1 | testicular cancer | -0.821 | <0.001 | 0.742 | <0.001 |
| 2 | causes of testicular cancer | -0.292 | <0.001 | 0.28 | <0.001 |
| 3 | symptoms for testicular cancer | -0.196 | 0.011 | 0.248 | 0.001 |
| 4 | testicular cancer ribbon | 0.342 | <0.001 | -0.275 | <0.001 |
| 5 | testicular cancer prognosis | -0.366 | <0.001 | 0.368 | <0.001 |
| 6 | testicular cancer risk factors | -0.185 | 0.016 | 0.217 | 0.005 |
| 7 | what does testicular cancer look like | 0.317 | <0.001 | -0.254 | 0.001 |
| 8 | how do you get testicular cancer | 0.32 | <0.001 | -0.214 | 0.005 |
| 9 | does testicular cancer spread | 0.297 | <0.001 | -0.217 | 0.005 |
| 10 | what are signs of testicular cancer | 0.557 | <0.001 | -0.476 | <0.001 |
|  | **Search Term** | **Incidence** | | **Mortality** | |
|  |  | Rincidence | **P** | Rmortality | **P** |
| 1 | mesothelioma | 0.45 | <0.001 | 0.446 | <0.001 |
| 2 | mesothelioma settlement | 0.193 | 0.012 | 0.195 | 0.012 |
| 3 | mesothelioma cells | -0.174 | 0.024 | -0.175 | 0.023 |
| 4 | treatment of mesothelioma | -0.158 | 0.041 | -0.162 | 0.036 |
