# Supplementary material for: Leveraging Internet Search Data to Improve the Prediction and Prevention of Noncommunicable Diseases: Retrospective Observational Study
Source: J Med Internet Res. 2020 Nov 12;22(11):e18998. doi: 10.2196/18998 (PMC7691086; doi:10.2196/18998)
Supplement: Multimedia Appendix 5 [file jmir_v22i11e18998_app5.doc]

1. Diabetes mellitus
2. Ischemic heart disease
3. Stroke
4. Atrial fibrillation and flutter
5. Breast cancer

1. Lung cancer

1. Colon and rectum cancer

1. Malignant skin melanoma

1. Non-Hodgkin lymphoma
2. Uterine cancer

1. Cardiomyopathy and myocarditis
2. Kidney cancer

1. Pancreatic cancer

1. Bladder cancer

1. Leukemia

1. Liver cancer
2. Stomach cancer

1. Lip and oral cavity cancer

1. Brain and nervous system cancer

1. Thyroid cancer
2. Multiple myeloma

1. Ovarian cancer

1. Cervical cancer

1. Esophageal cancer
2. Larynx cancer

1. Gallbladder and biliary tract cancer

1. Hodgkin lymphoma

1. Testicular cancer
2. Mesothelioma
