# Supplementary material for: Leveraging Internet Search Data to Improve the Prediction and Prevention of Noncommunicable Diseases: Retrospective Observational Study
Source: J Med Internet Res. 2020 Nov 12;22(11):e18998. doi: 10.2196/18998 (PMC7691086; doi:10.2196/18998)

1. Diabetes mellitus

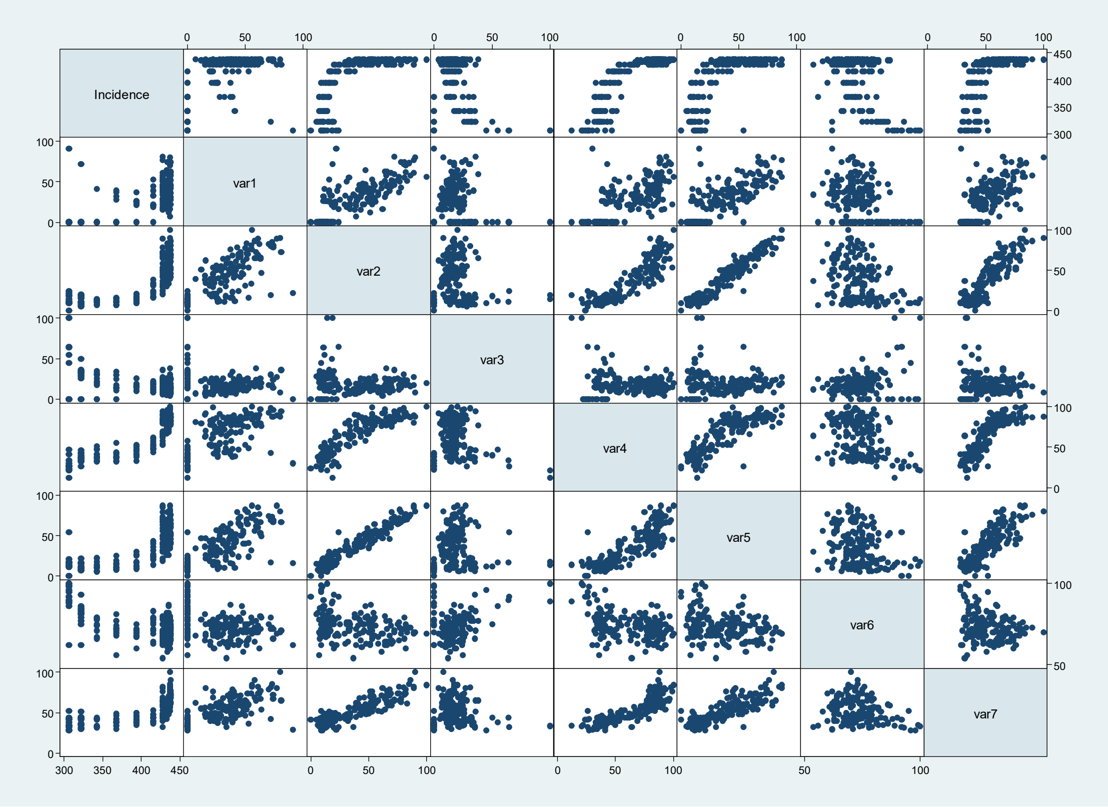

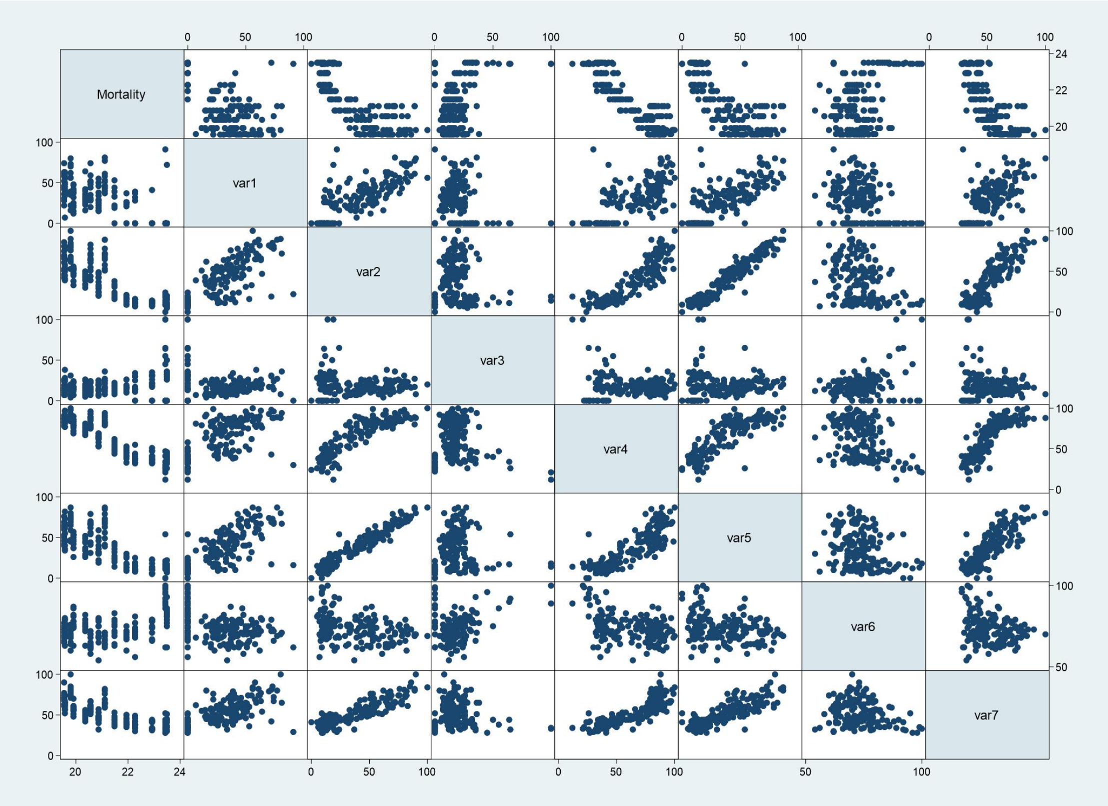

2. Ischemic heart disease

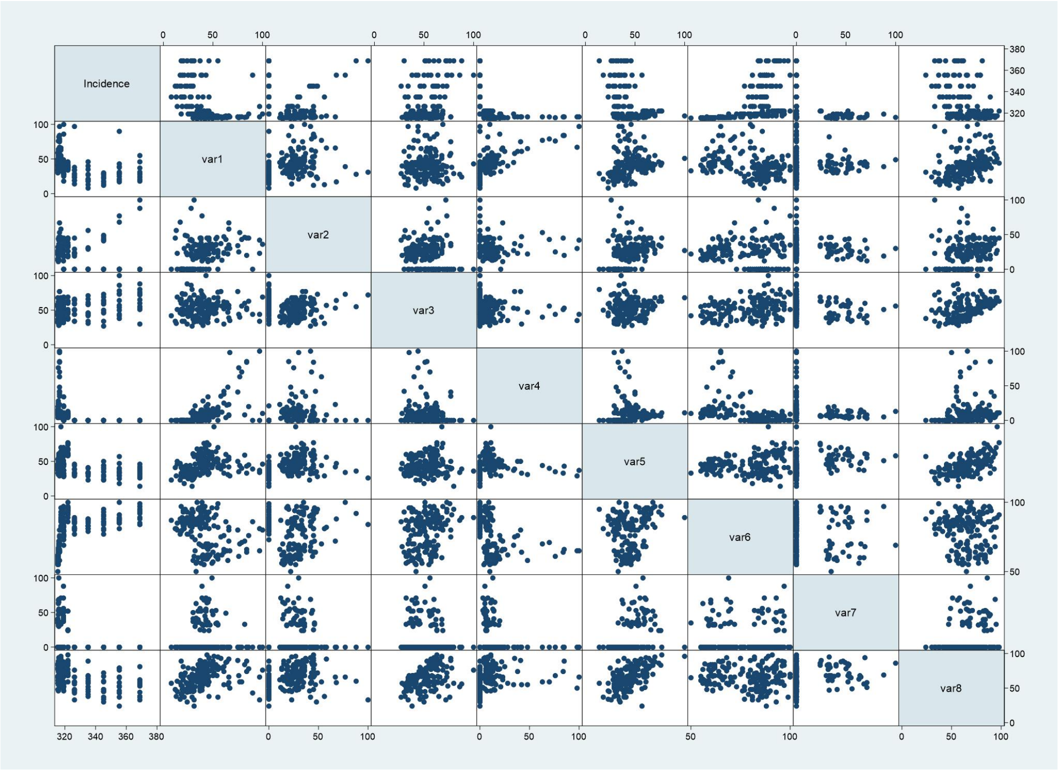

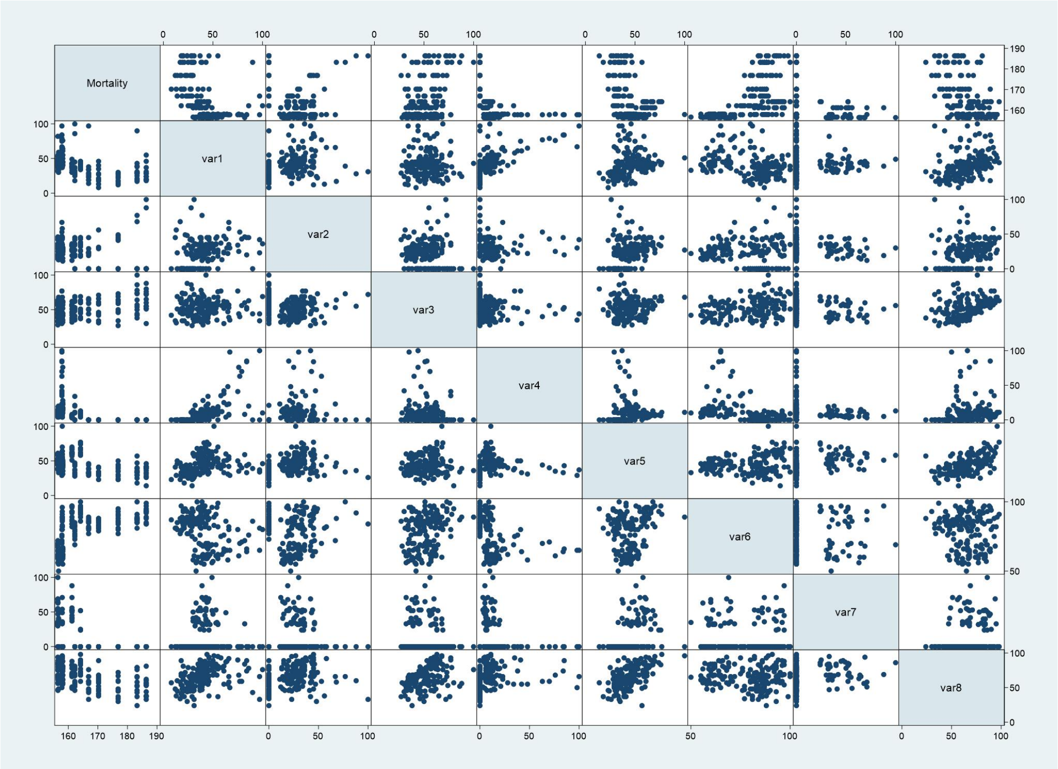

3. Stroke

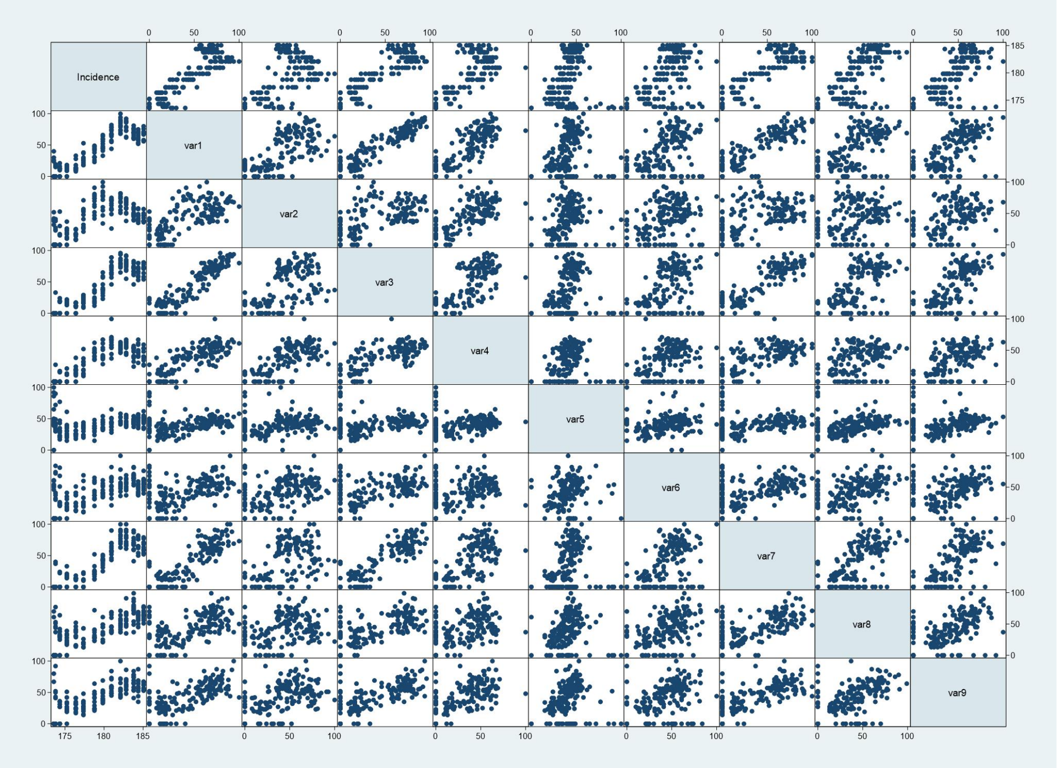

Incidence
var1
var2
var3
var4
var5
var6
var7
var8
var9

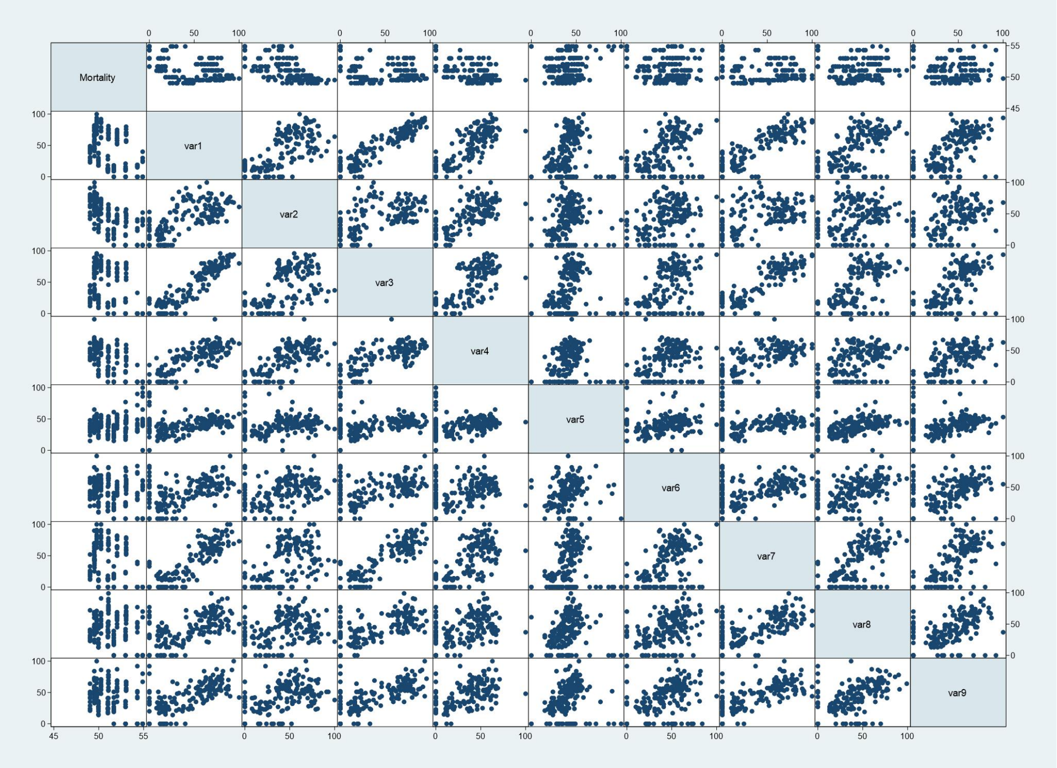

Mortality
var1
var2
var3
var4
var5
var6
var7
var8
var9

4. Atrial fibrillation and flutter

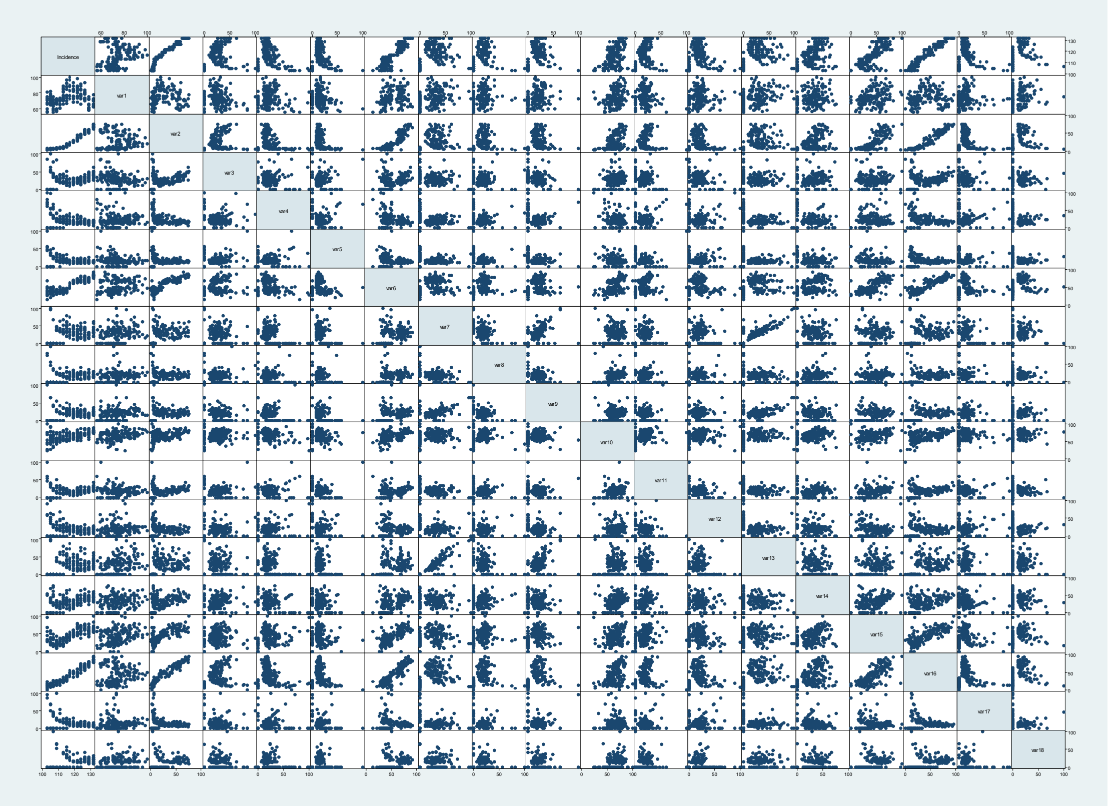

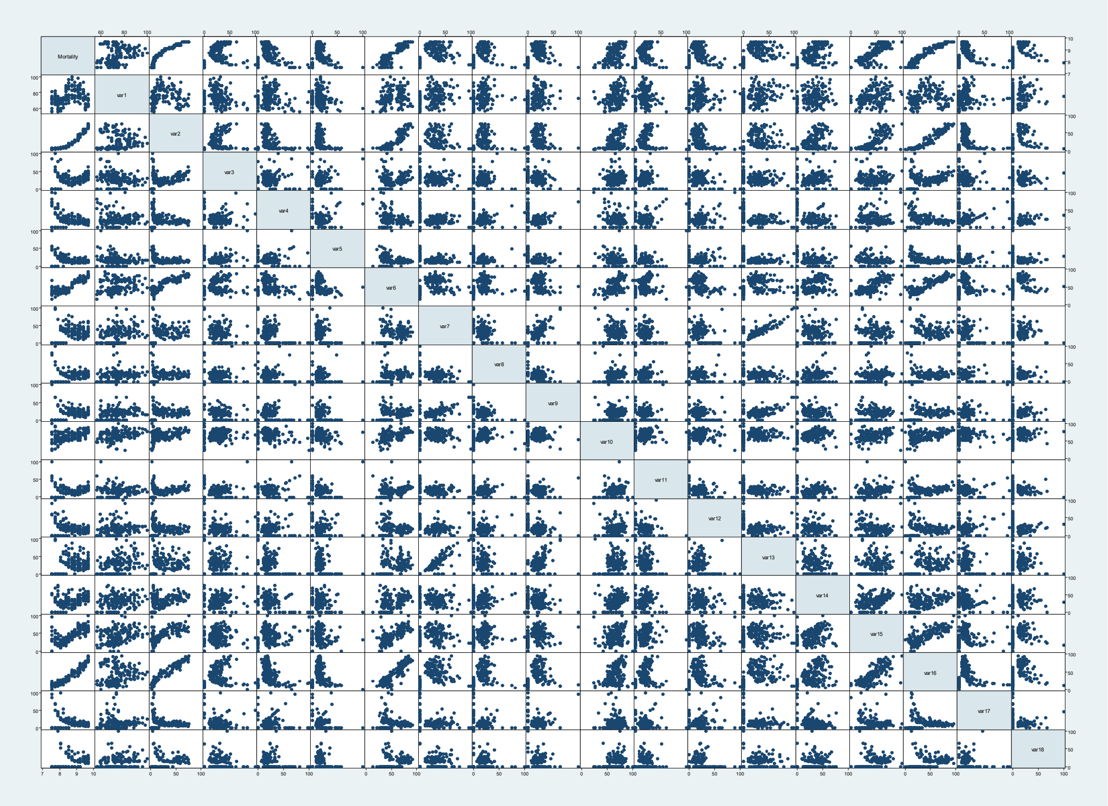

5. Breast cancer

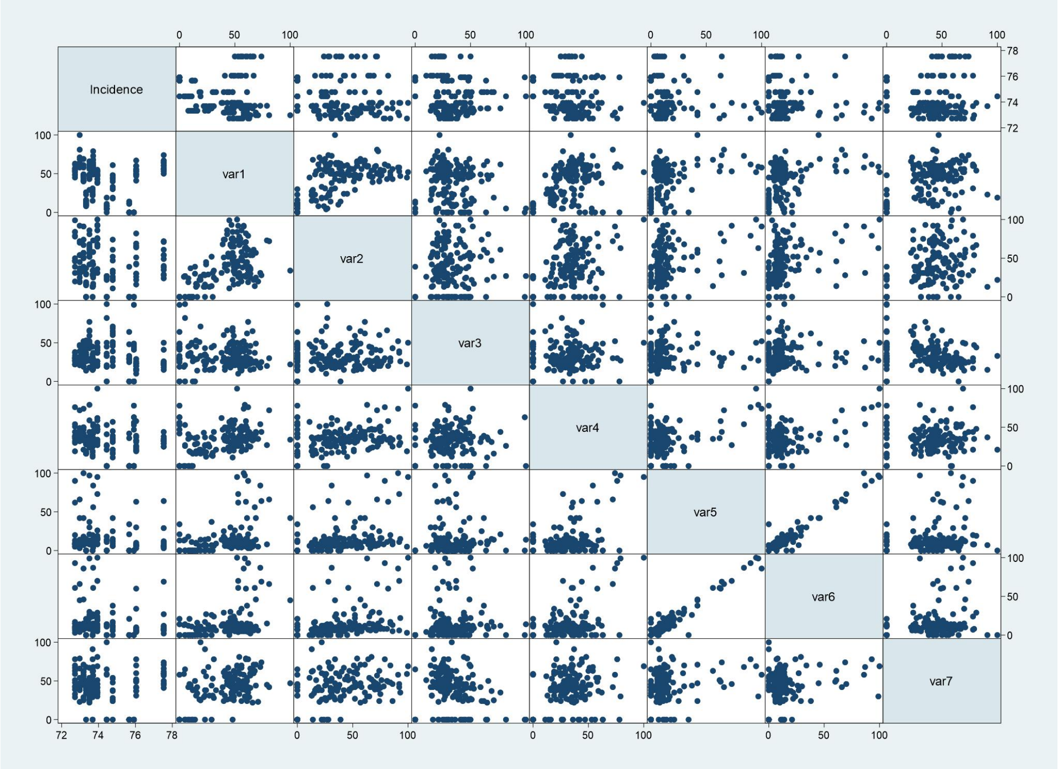

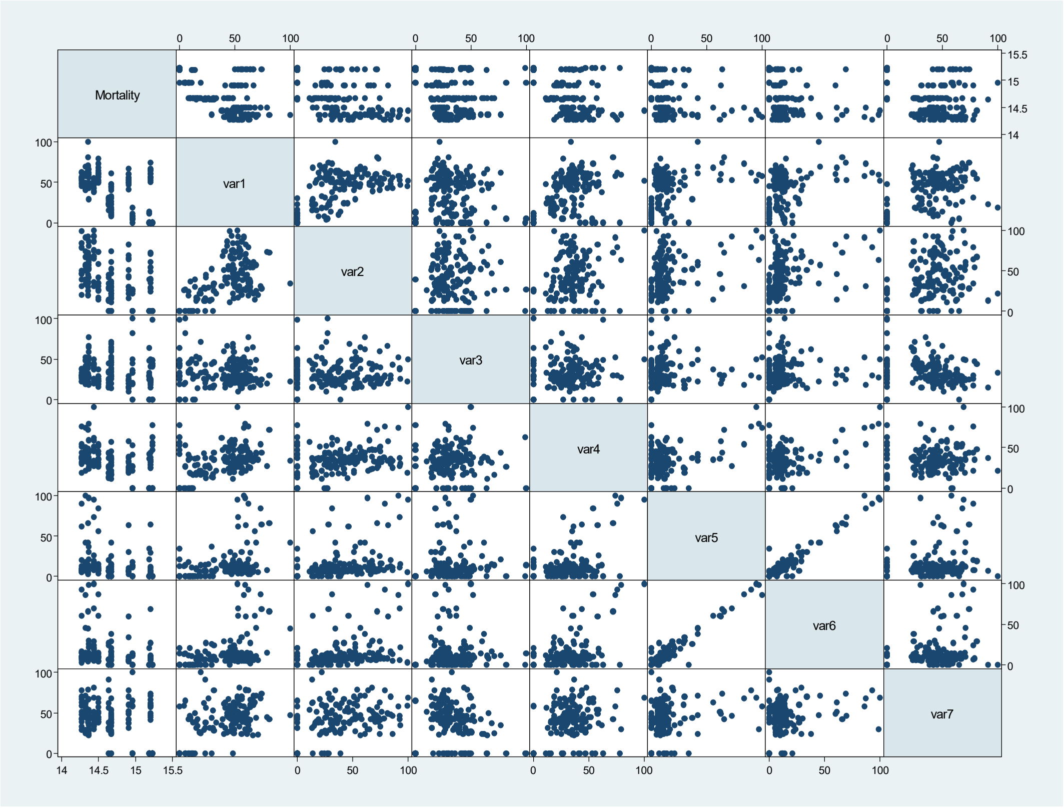

6. Lung cancer

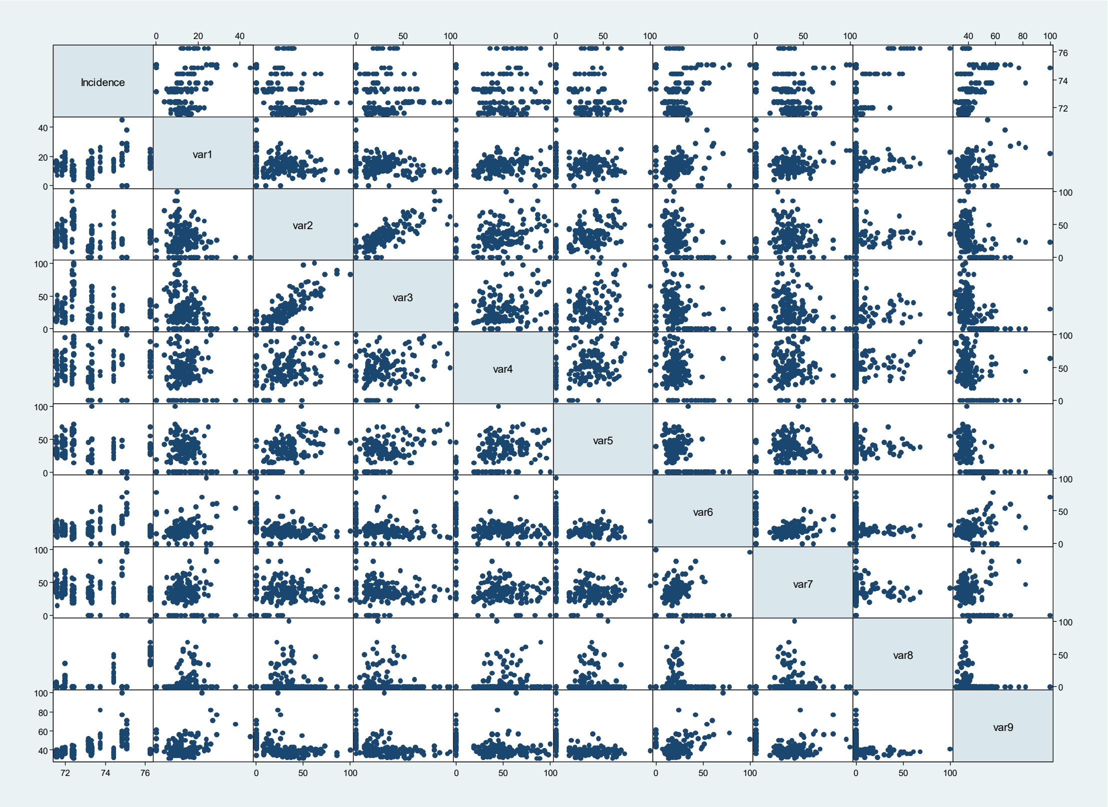

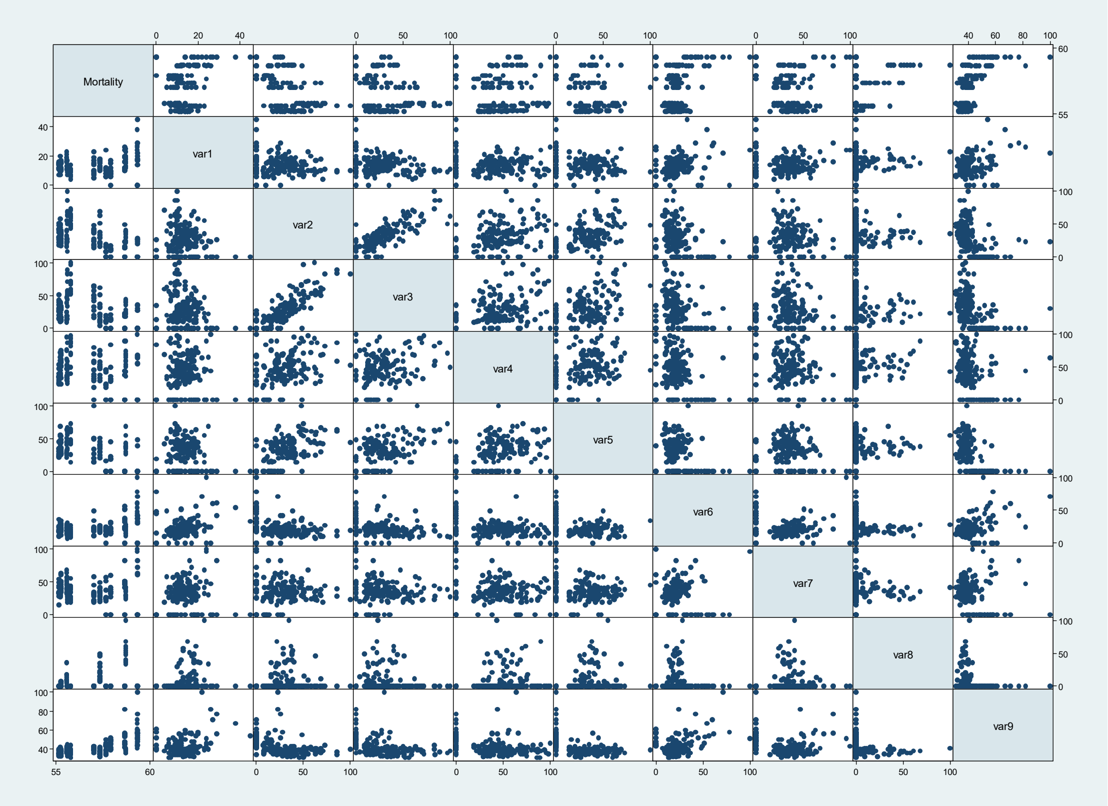

7. Colon and rectum cancer

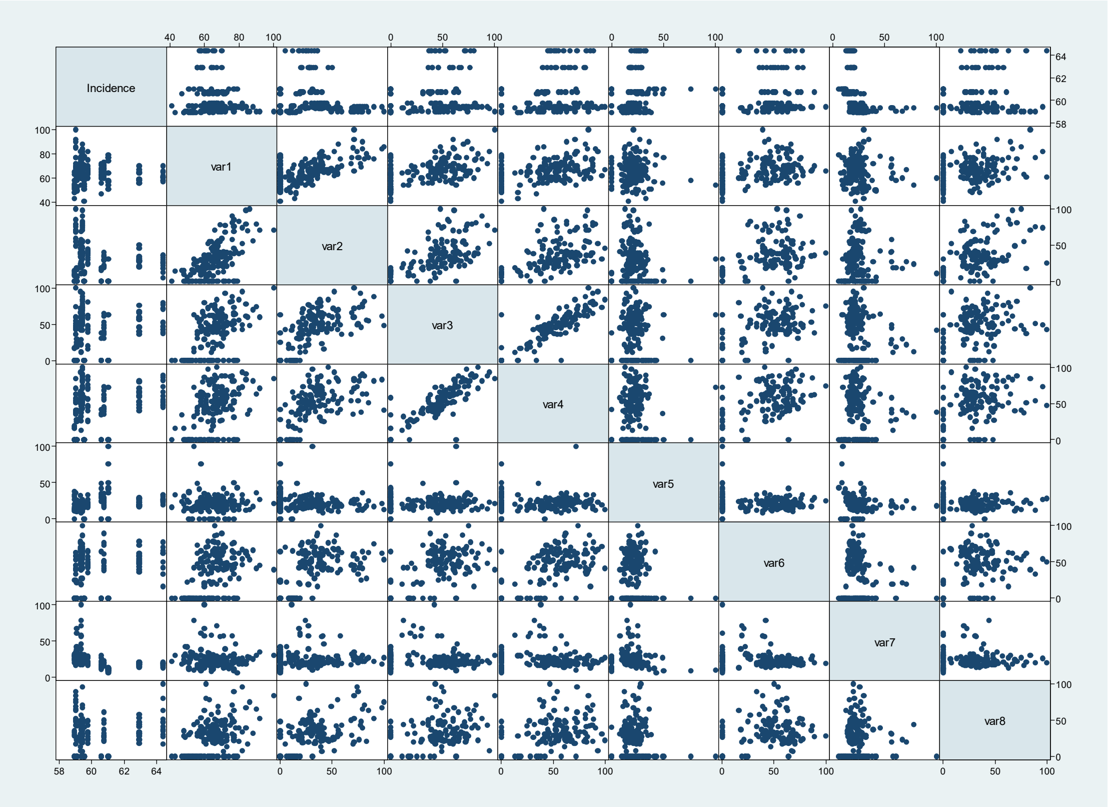

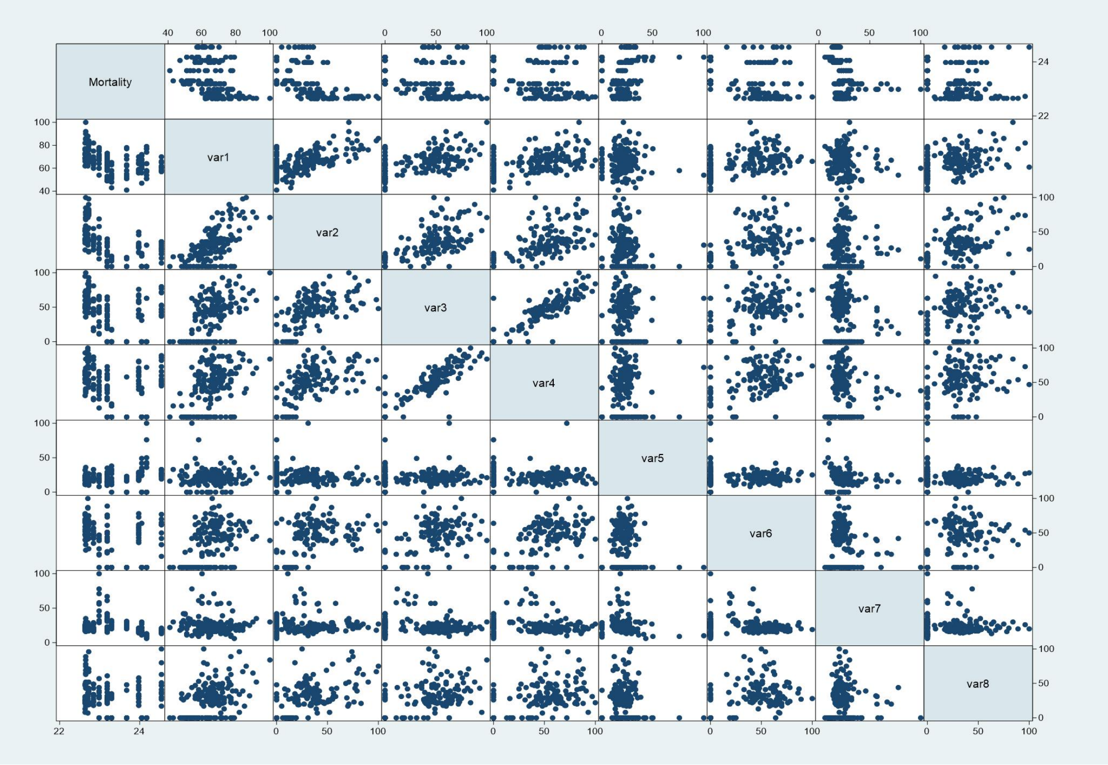

8. Malignant skin melanoma

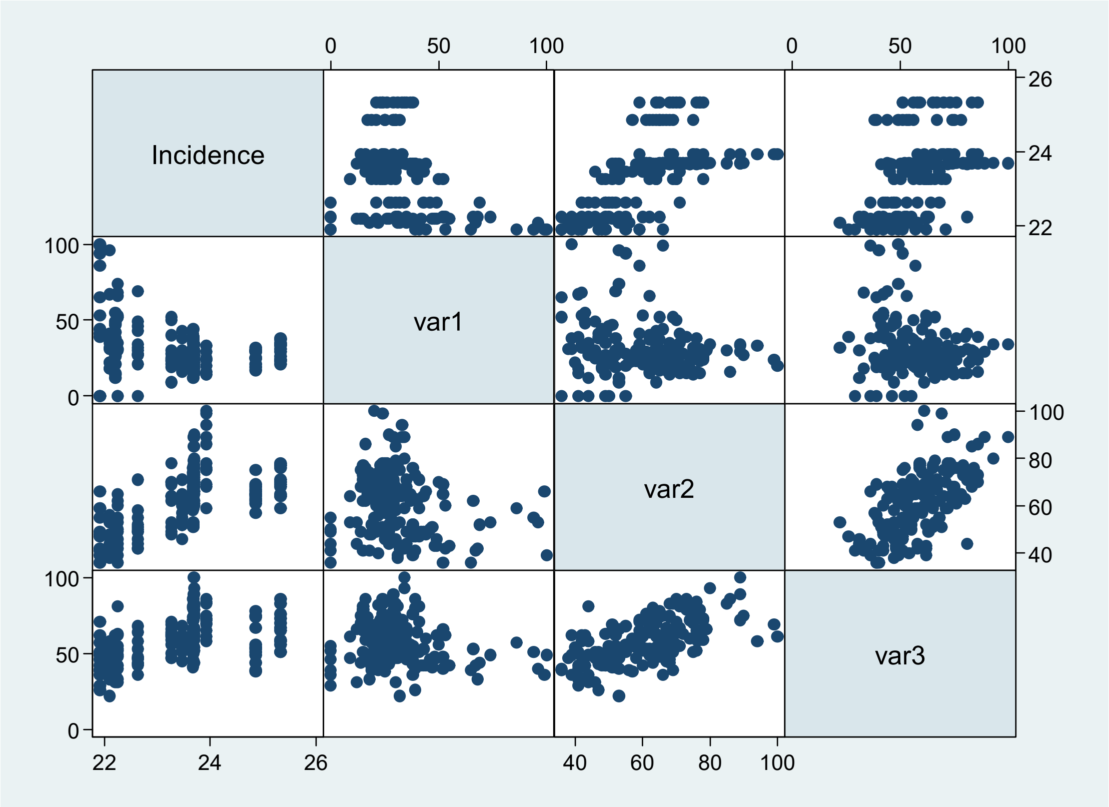

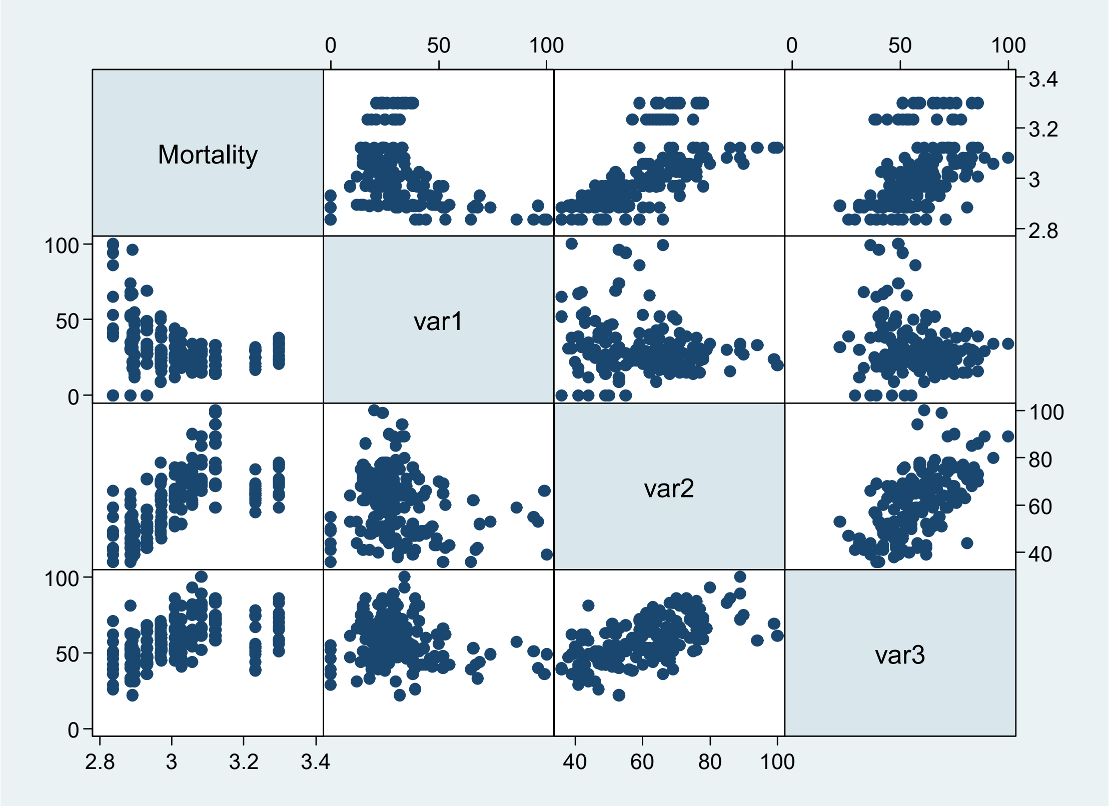

9. Non-Hodgkin lymphoma

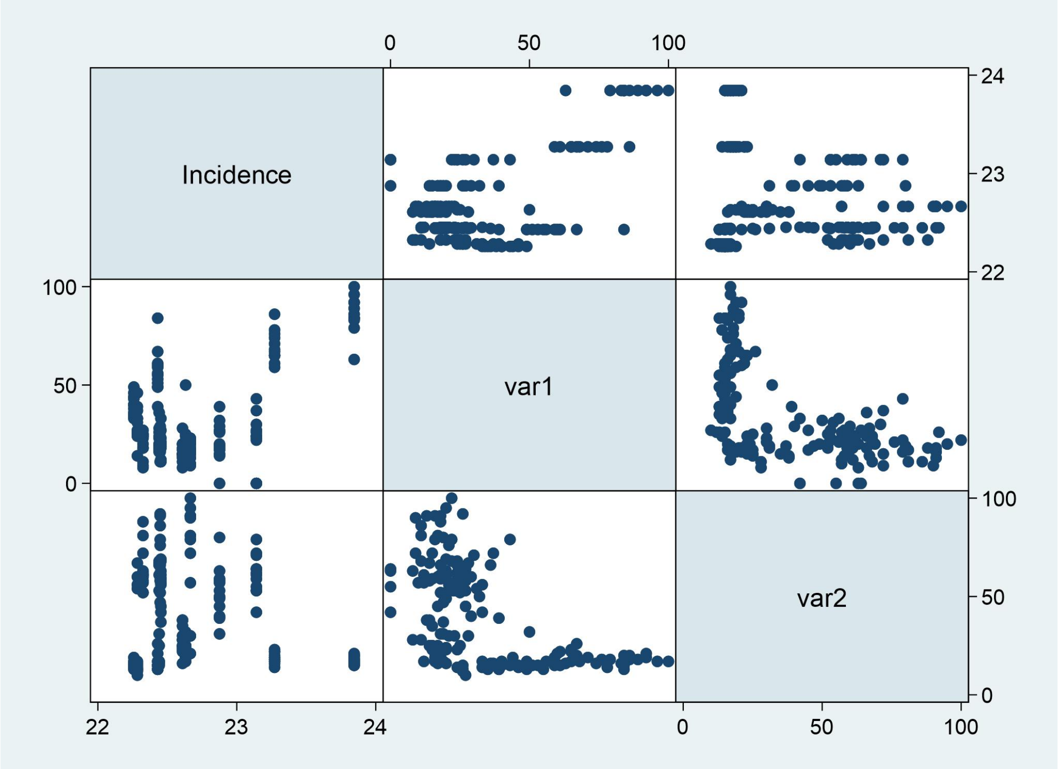

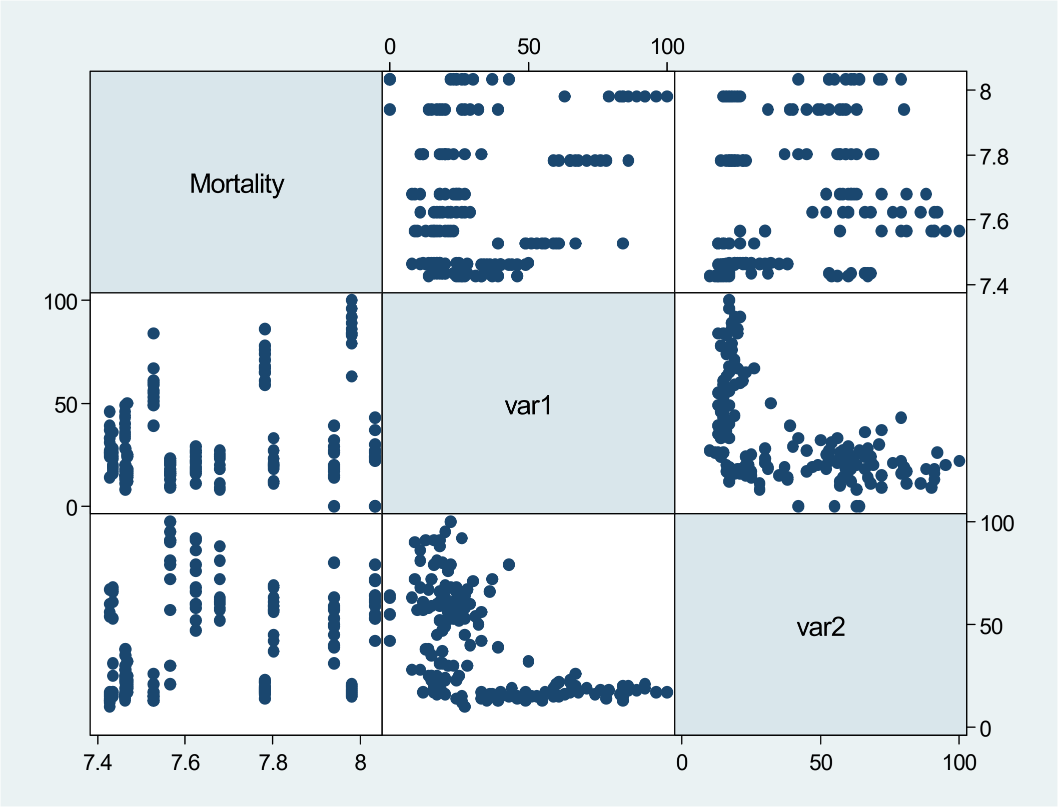

10. Uterine cancer

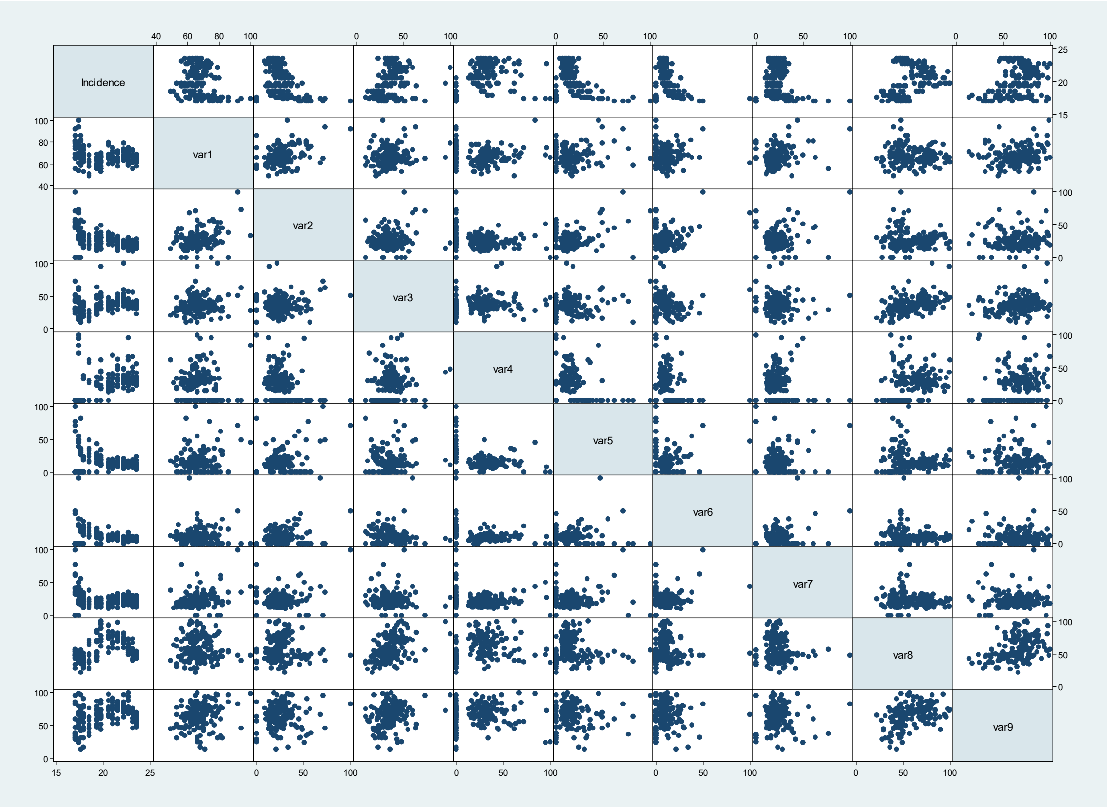

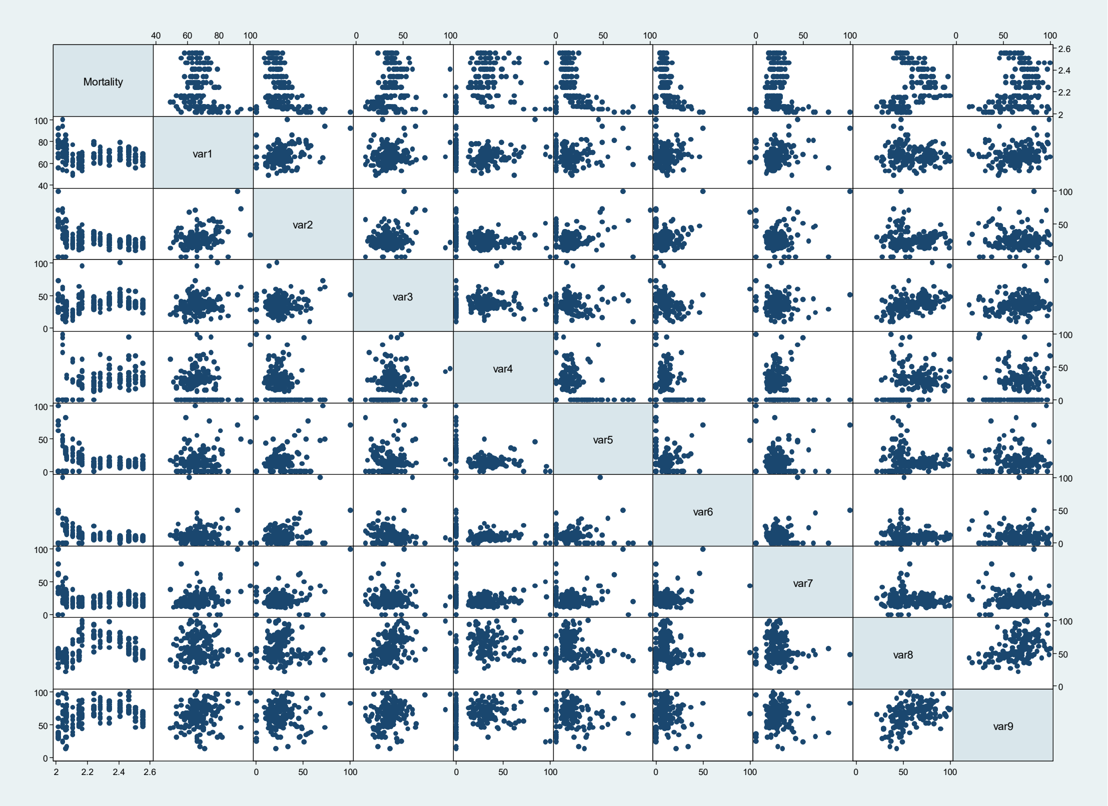

11. Cardiomyopathy

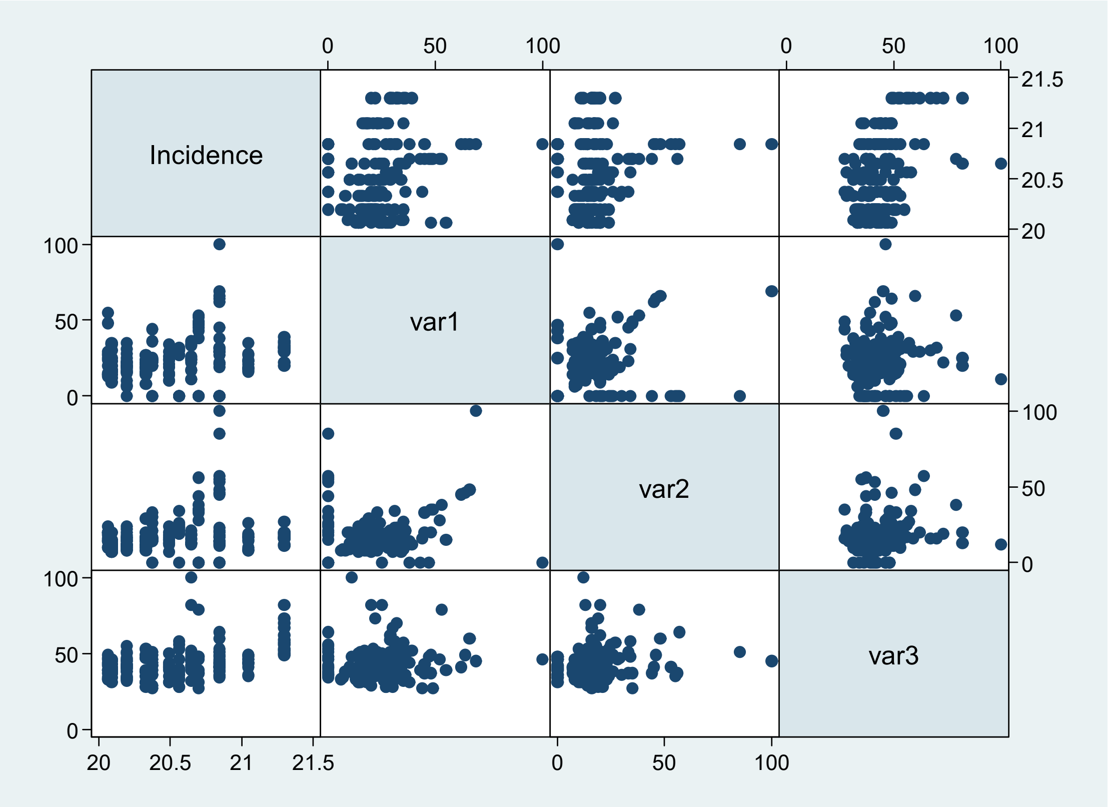

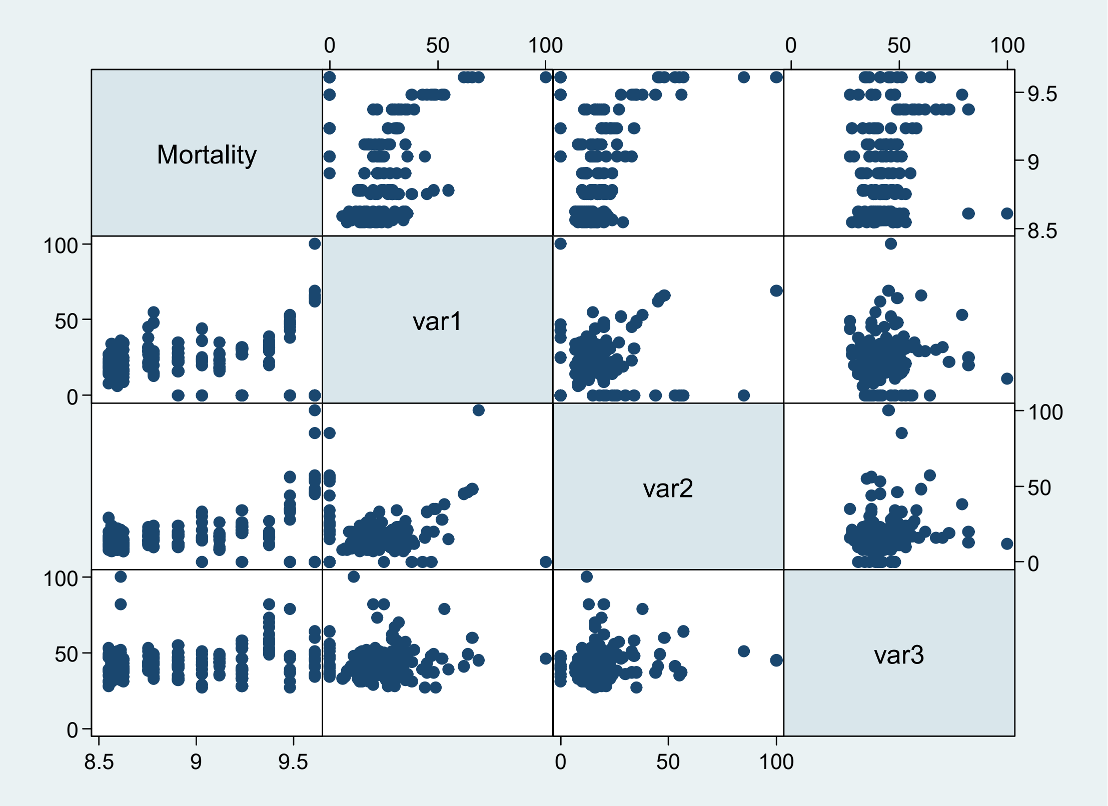

12. Kidney cancer

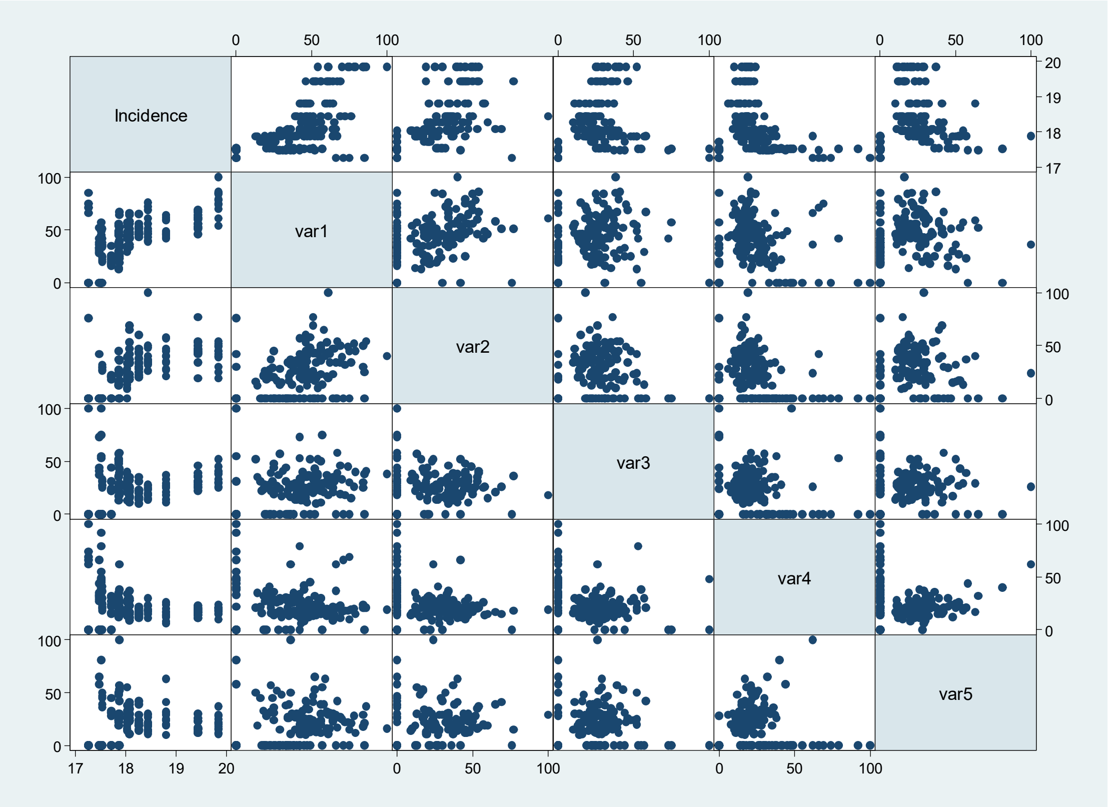

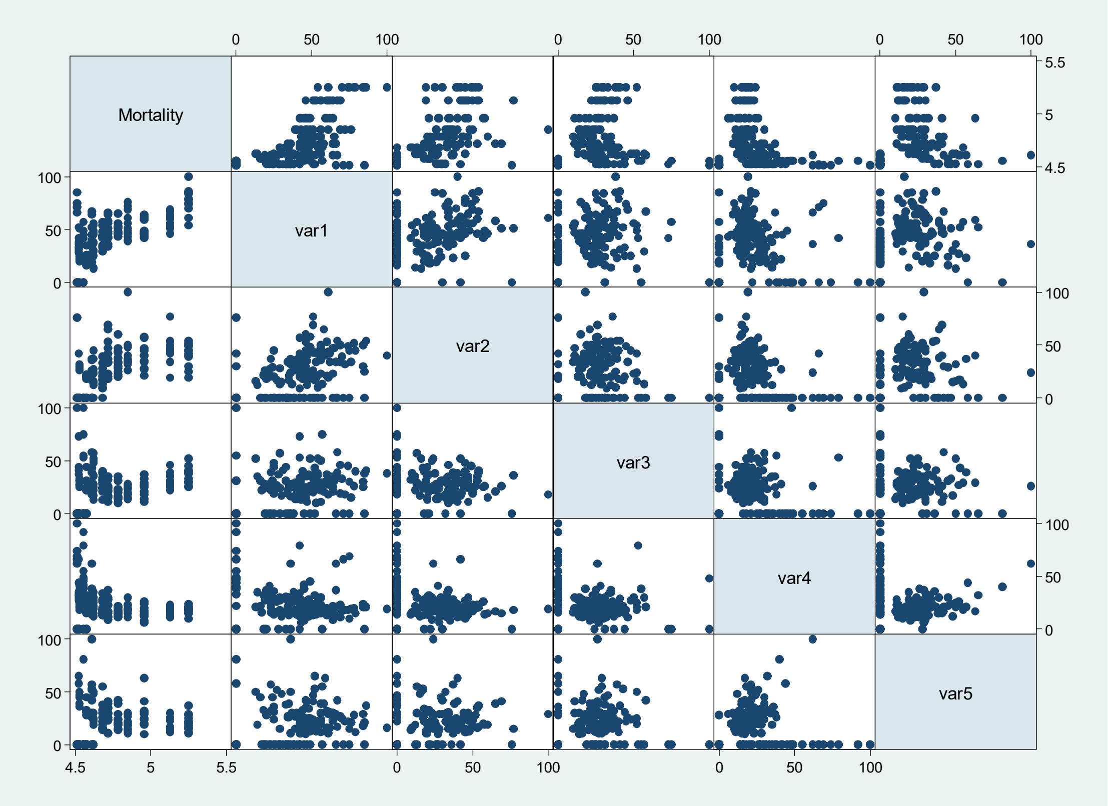

13. Pancreatic cancer

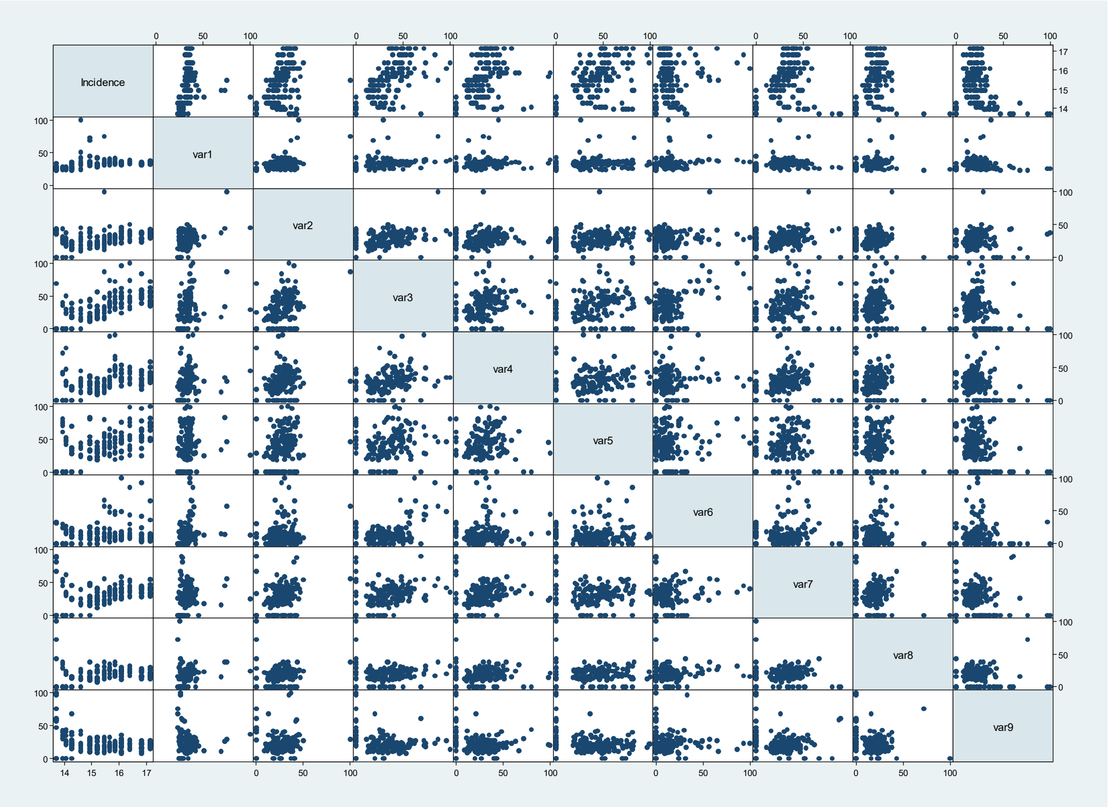

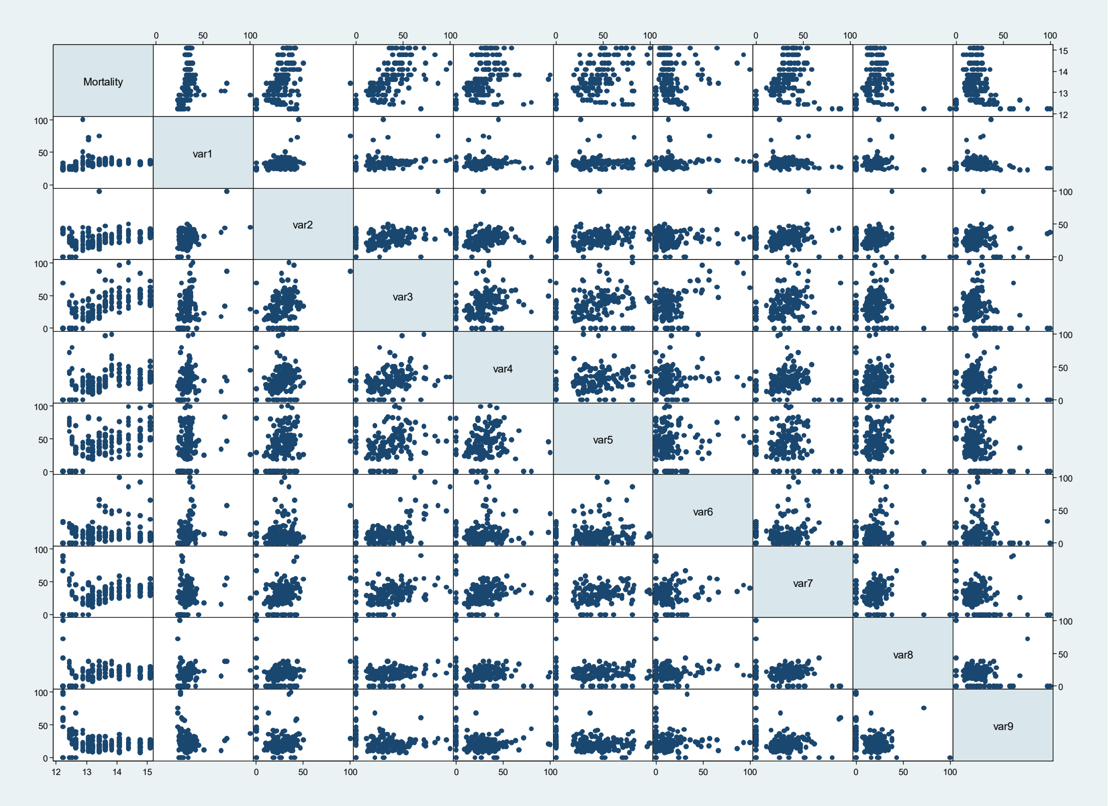

14. Bladder cancer

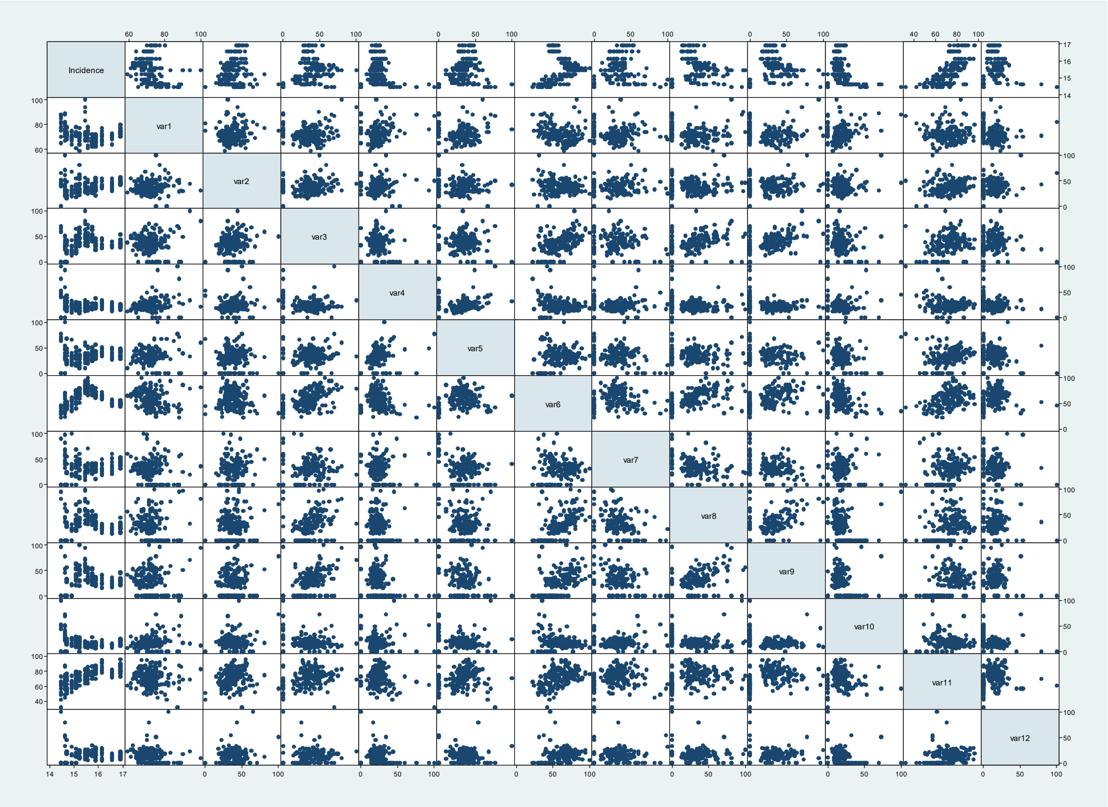

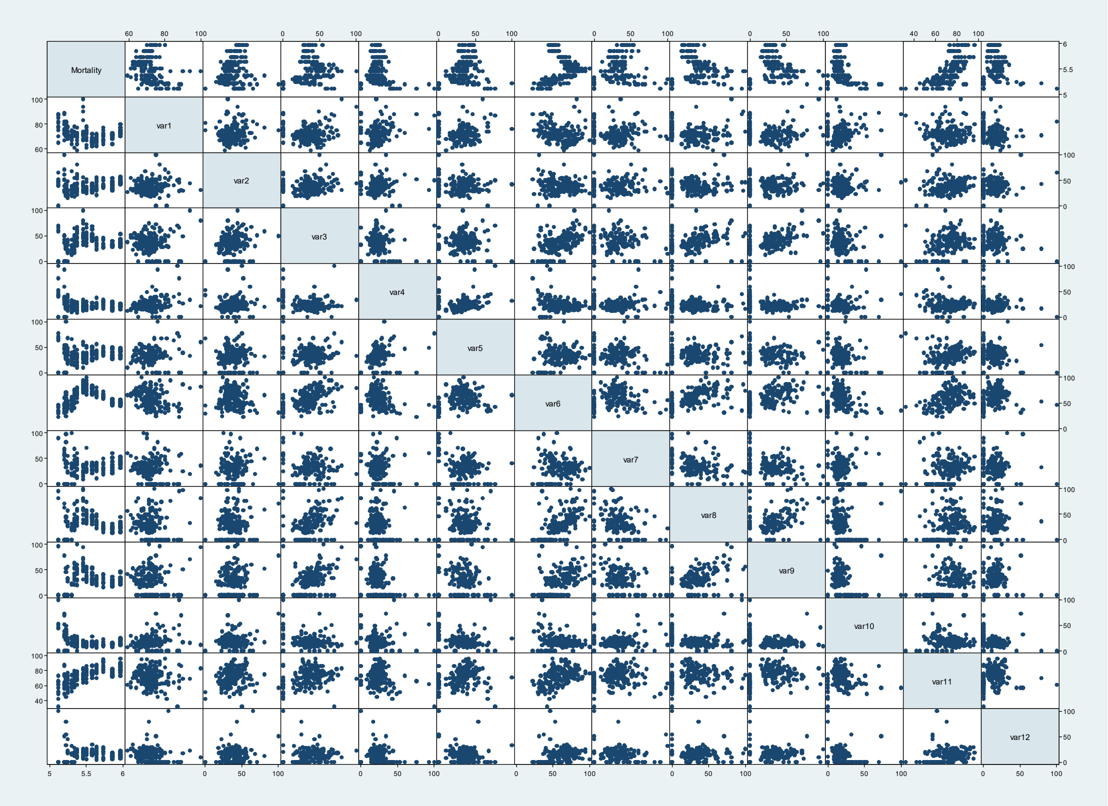

15. Leukemia

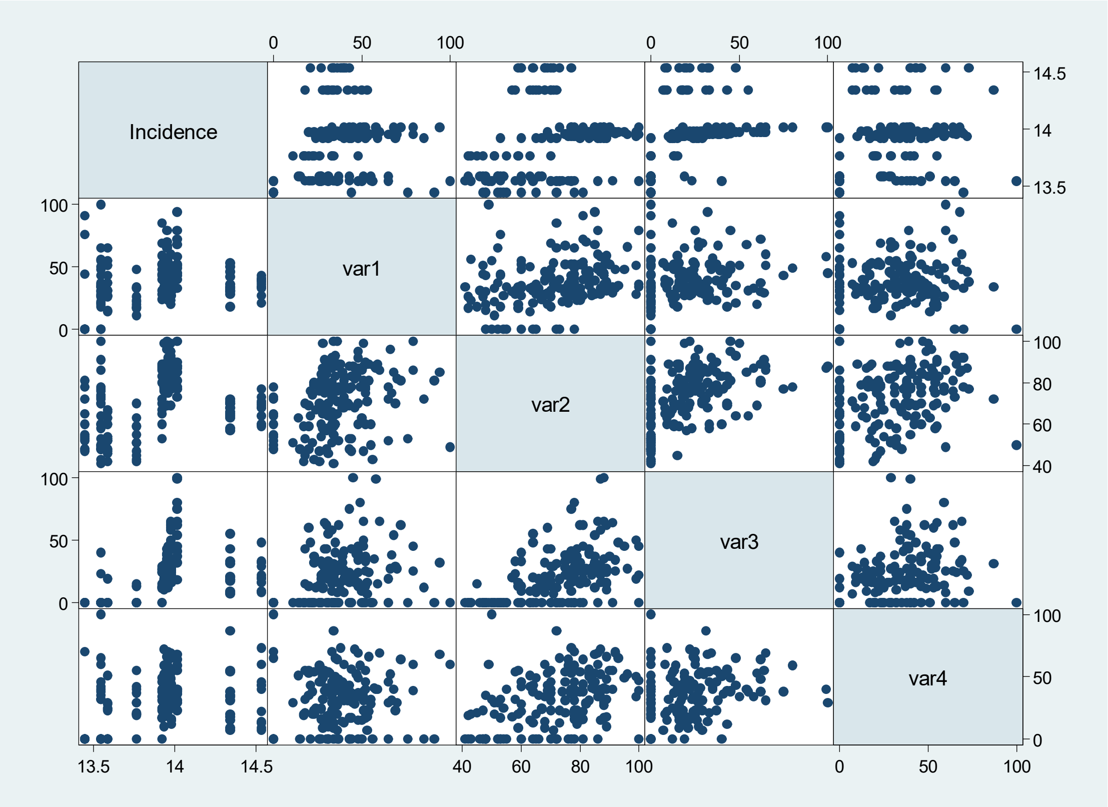
Incidence
var1
var2
var3
var4
0 50 100
0 50 100
14.5
14
13.5
100
50
0
100
80
60
40
100
50
0
100
50
0
13.5 14 14.5
40 60 80 100
0 50 100

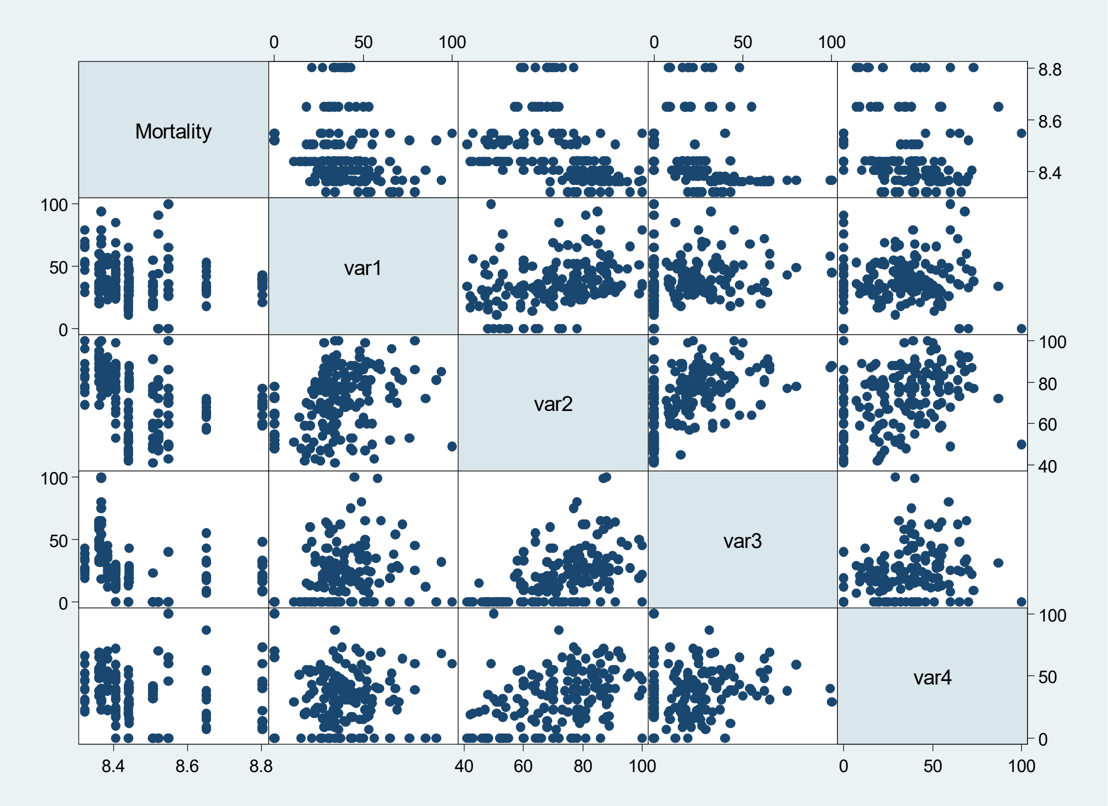
Mortality
var1
var2
var3
var4
0 50 100
0 50 100
8.8
8.6
8.4
100
50
0
100
80
60
40
100
50
0
100
50
0
8.4 8.6 8.8
40 60 80 100
0 50 100

16. Liver cancer

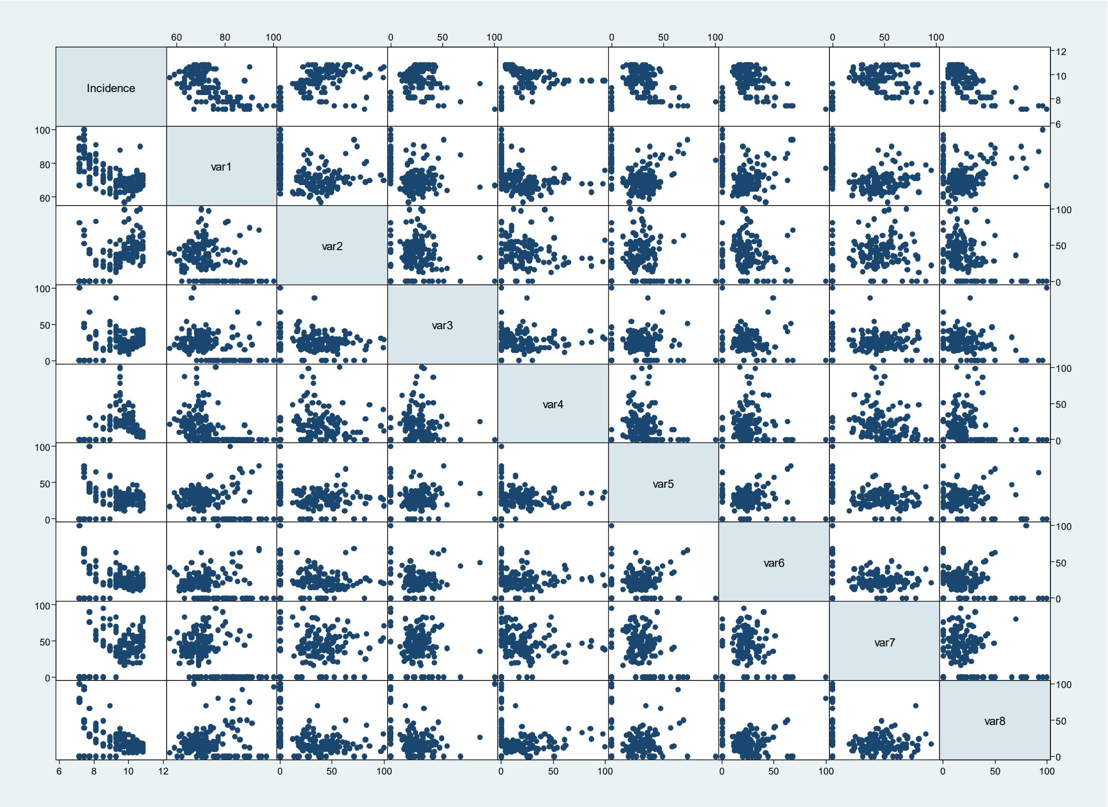

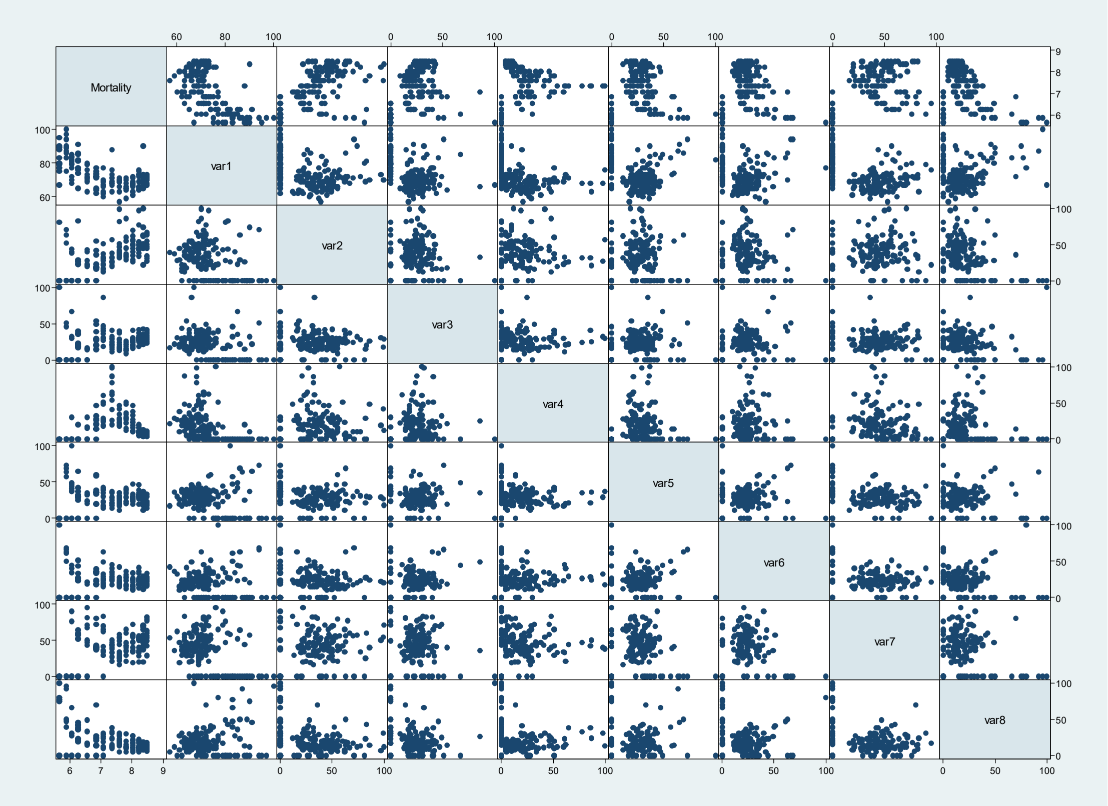

17. Stomach cancer

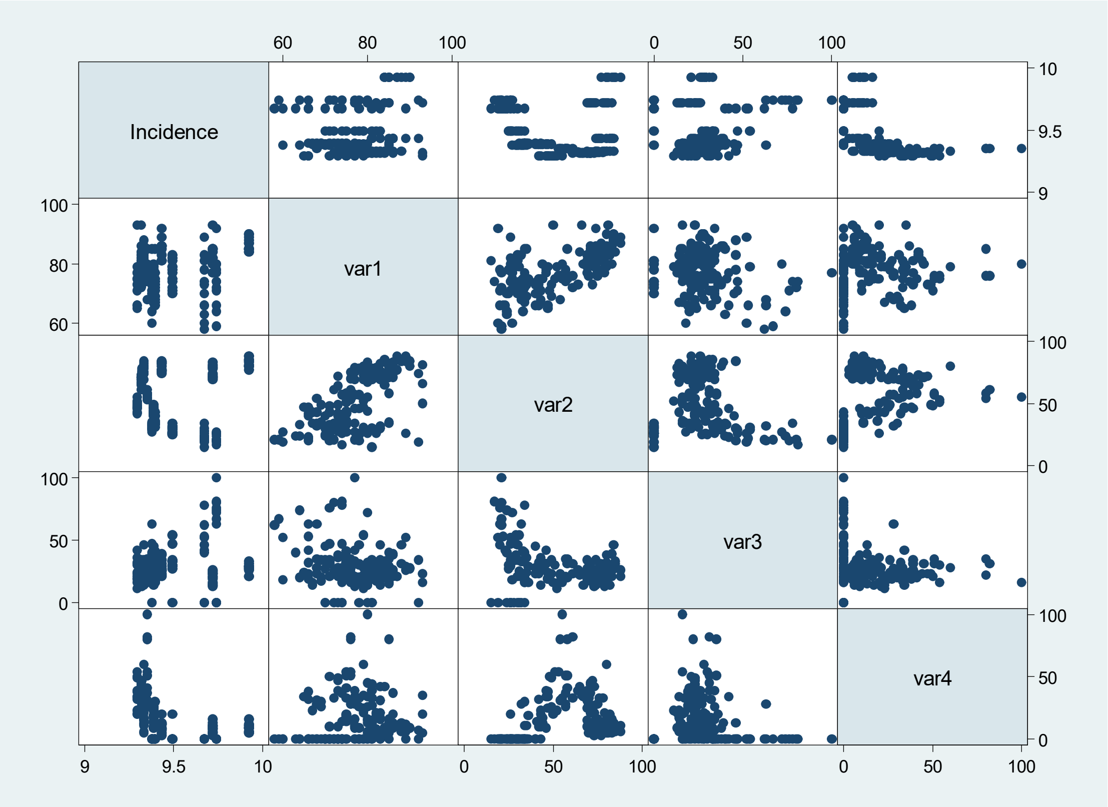

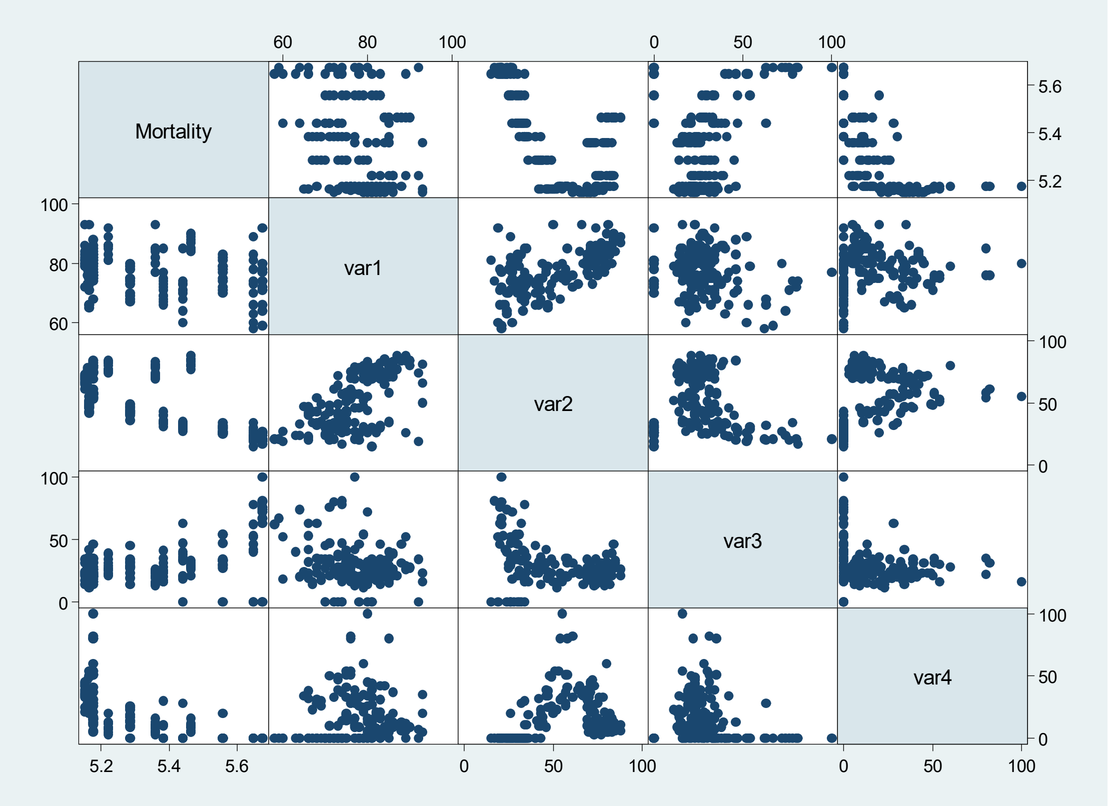

18. Lip and oral cavity cancer

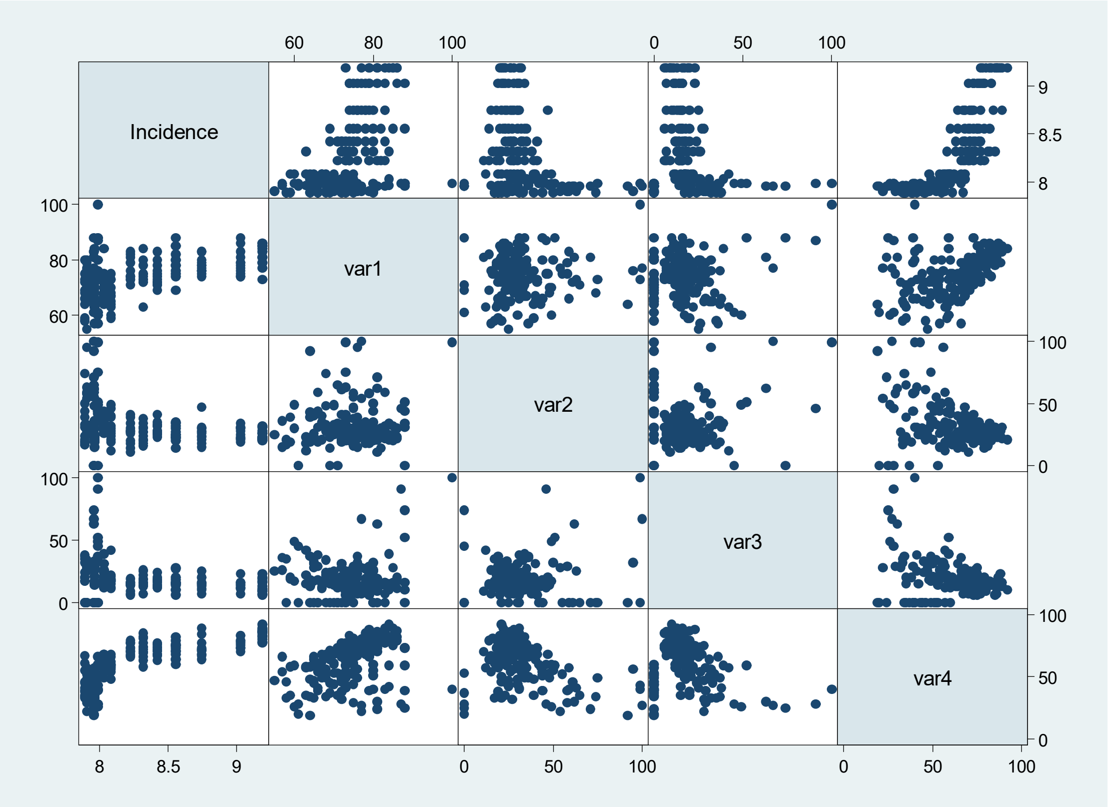

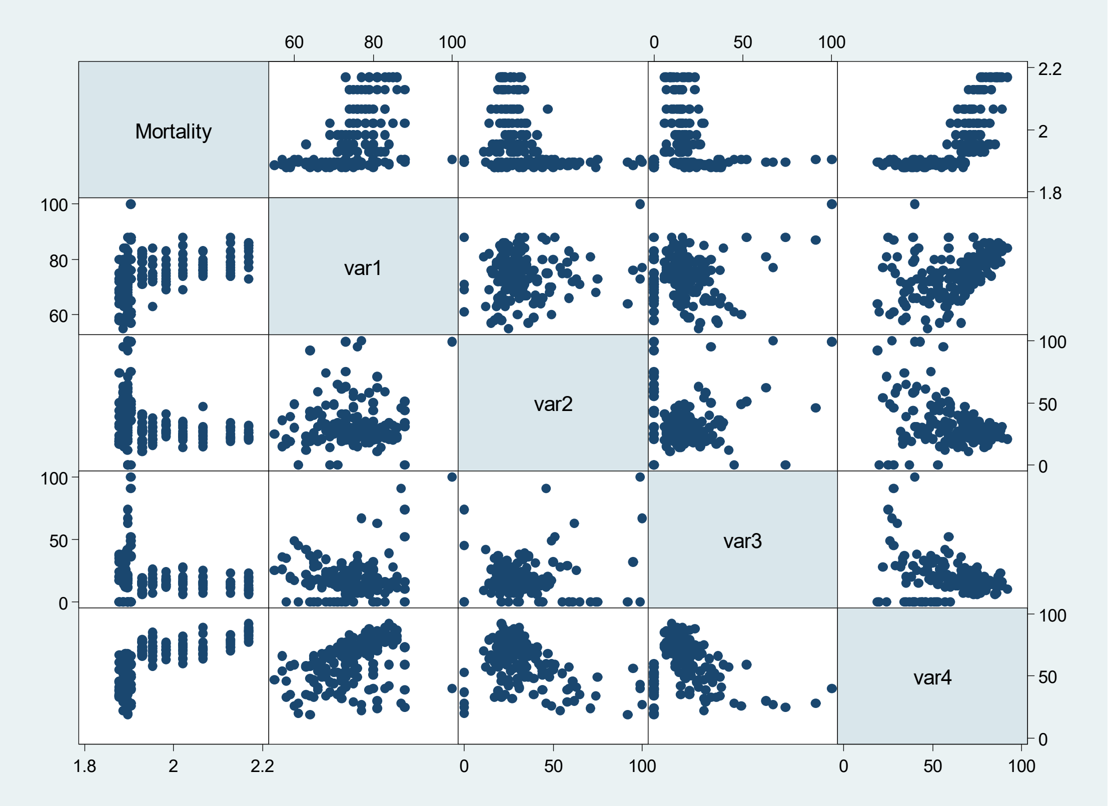

19. Brain and nervous system cancer

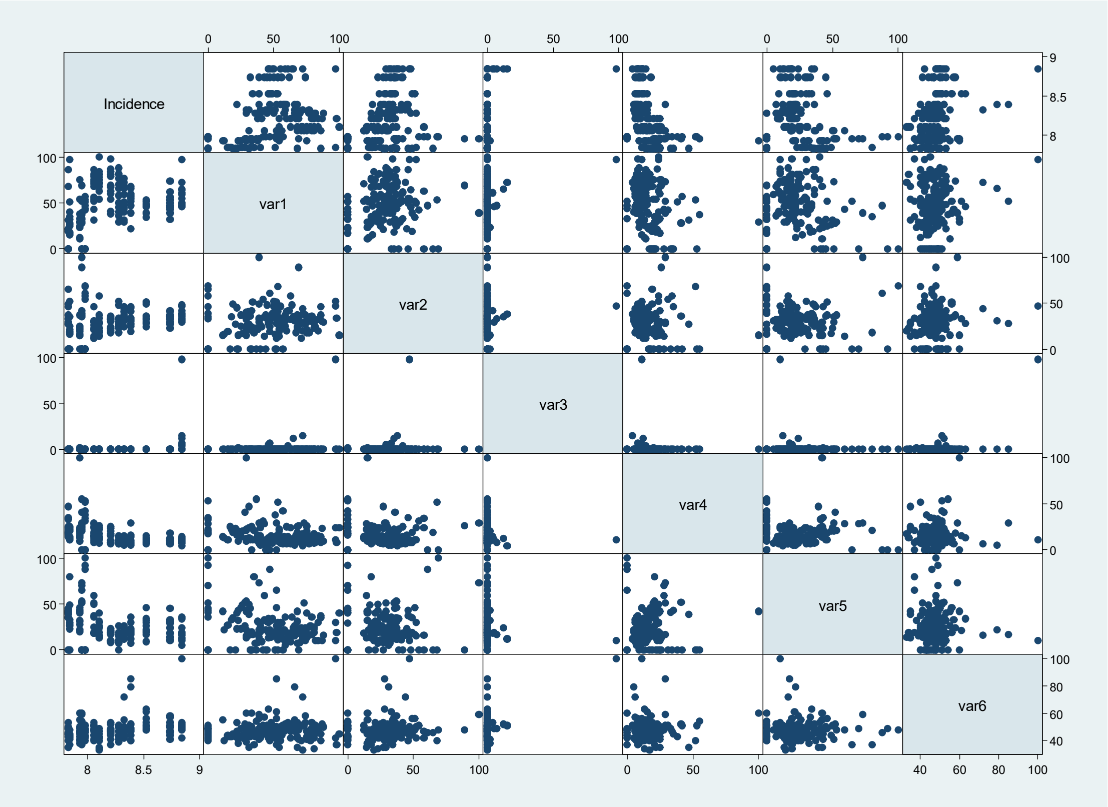

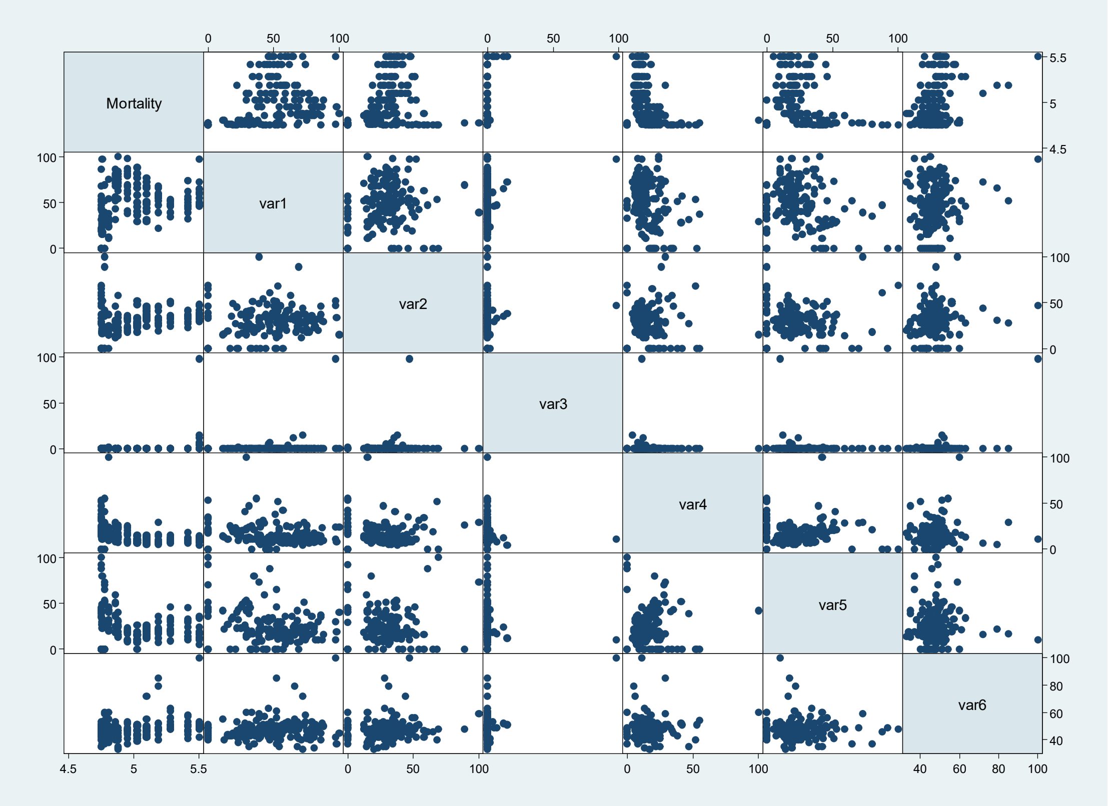

20. Thyroid cancer

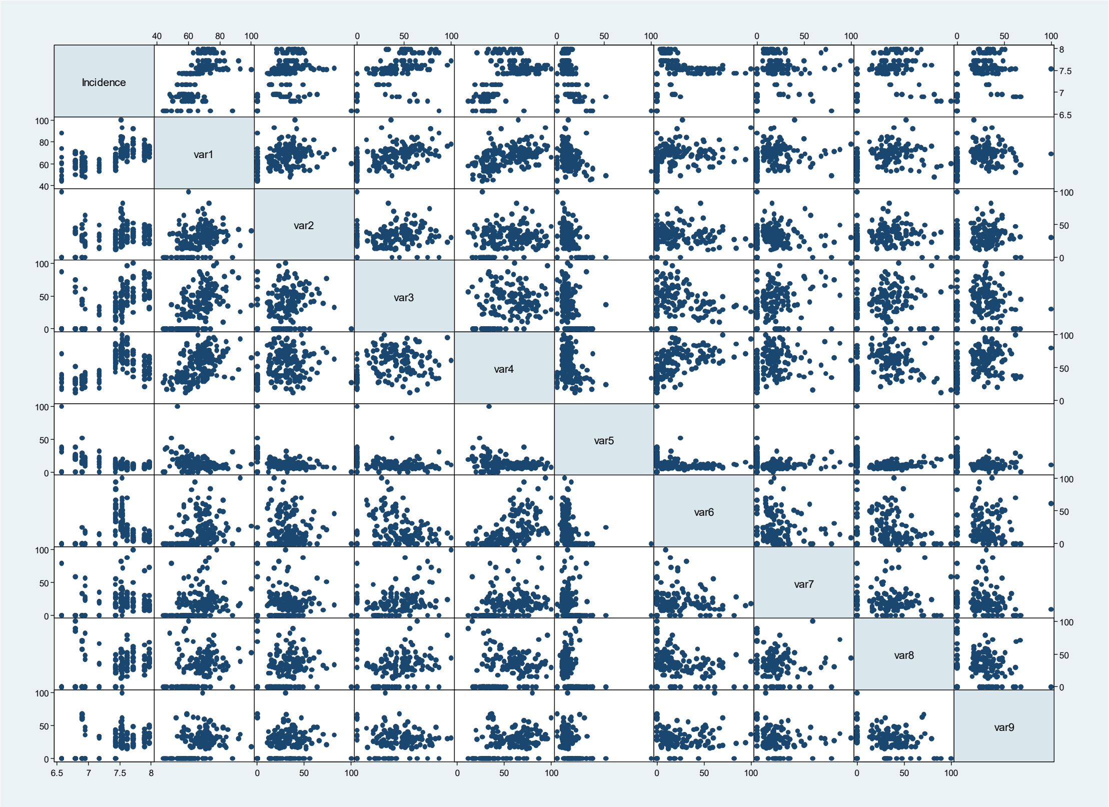

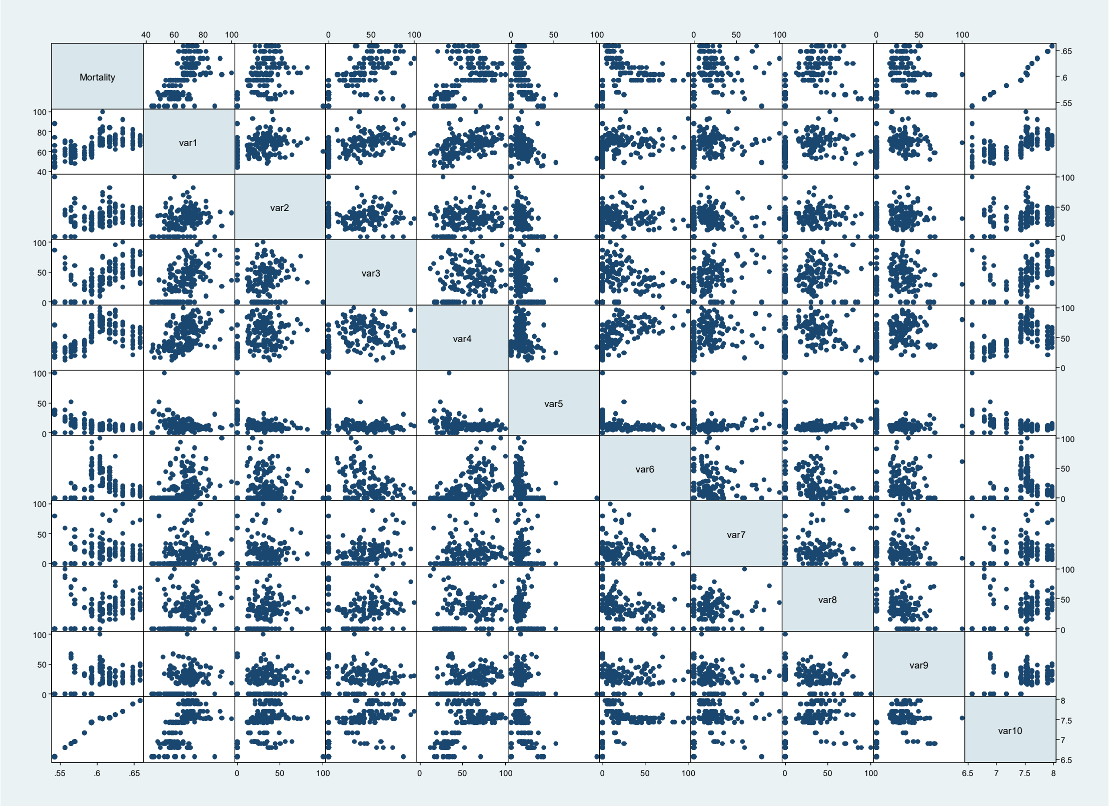

21. Multiple myeloma

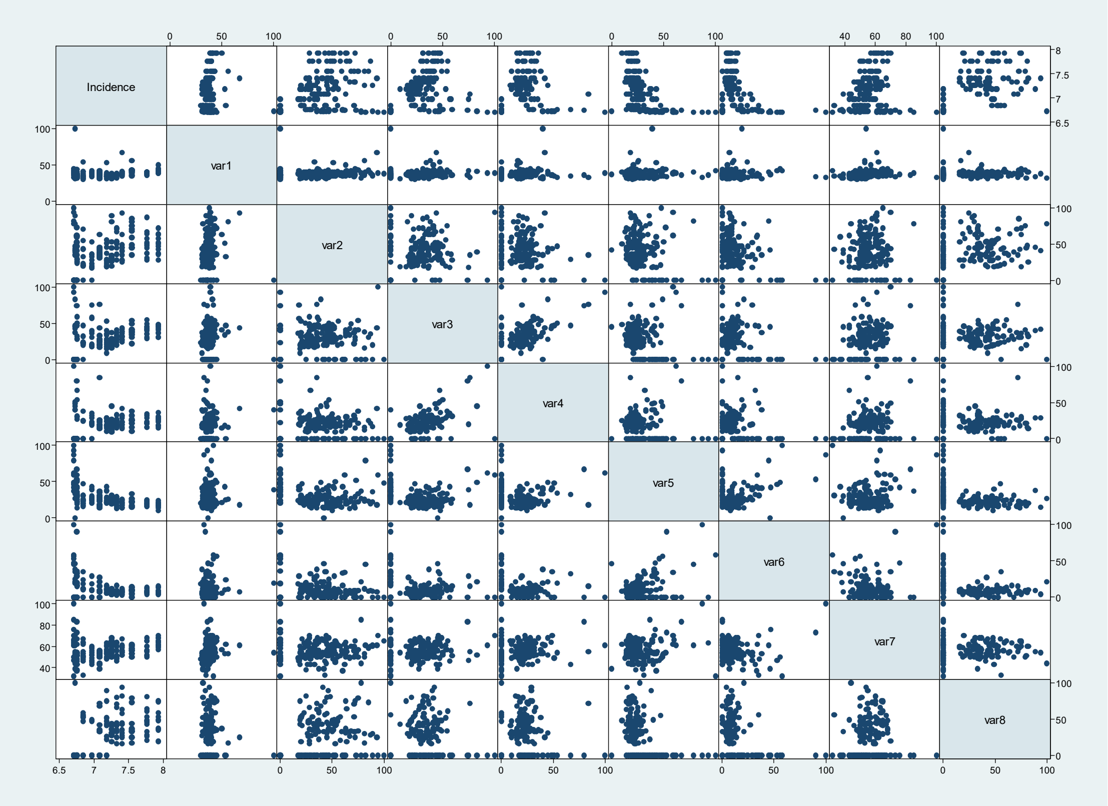

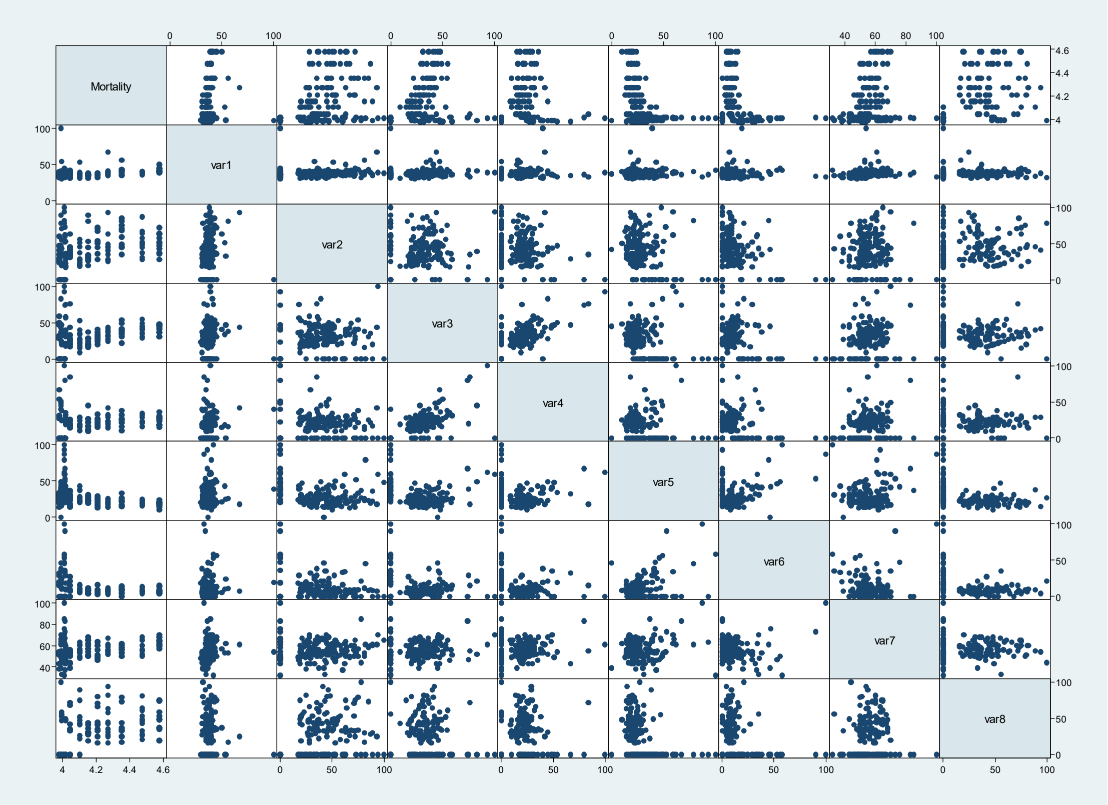

22. Ovarian cancer

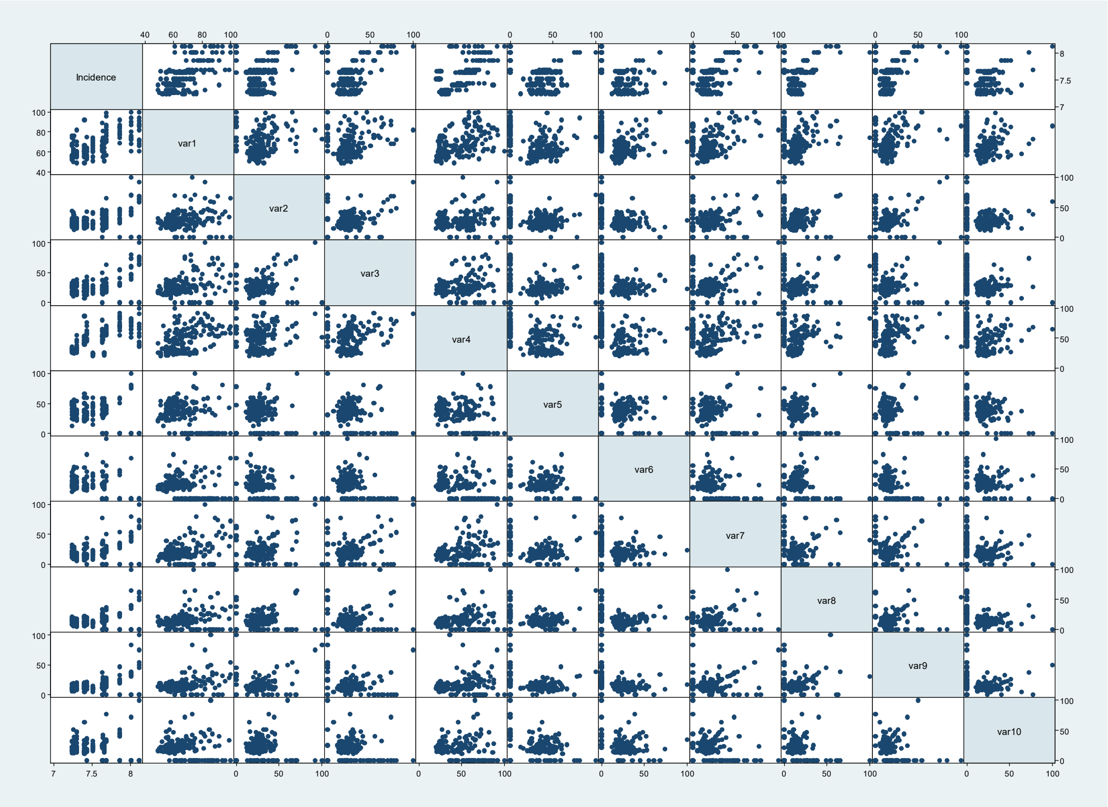

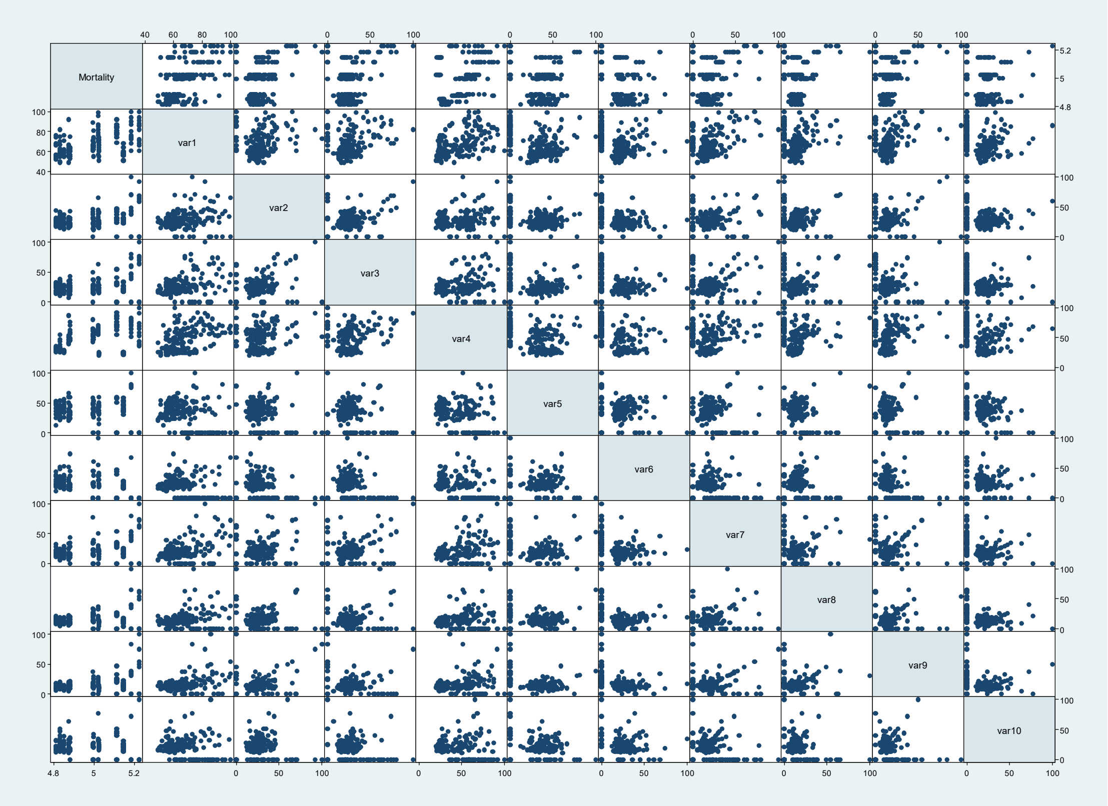

23. Cervical cancer

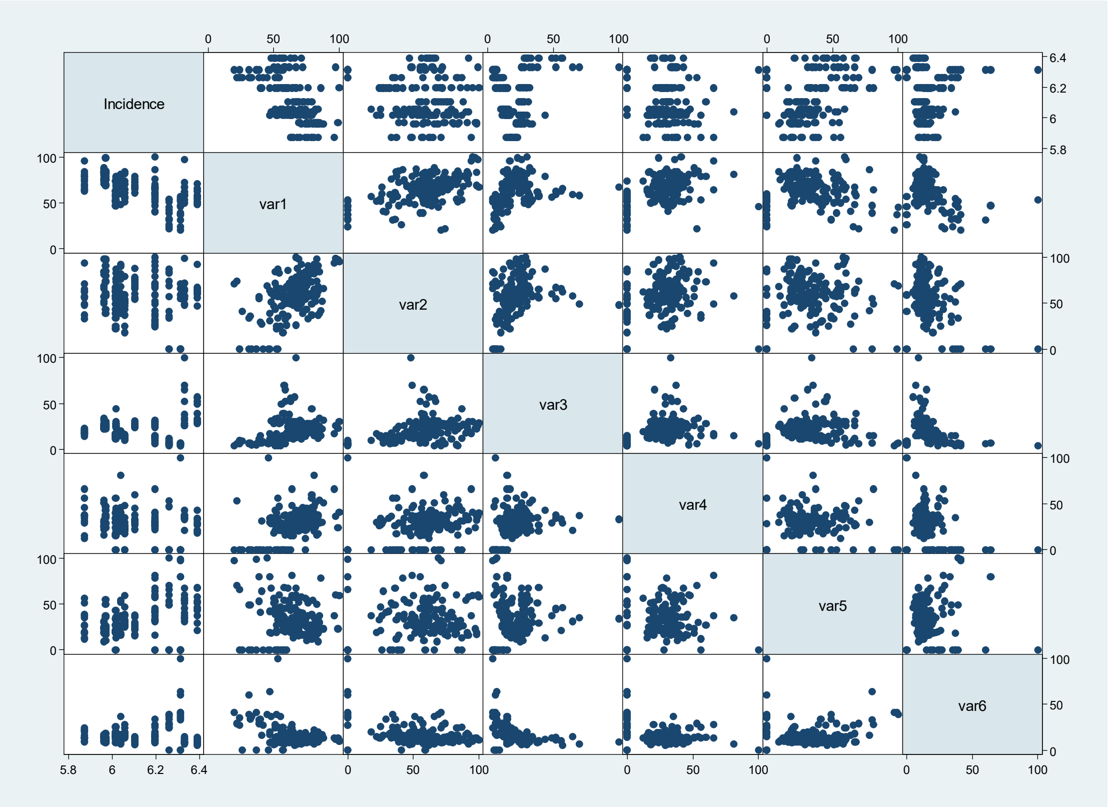

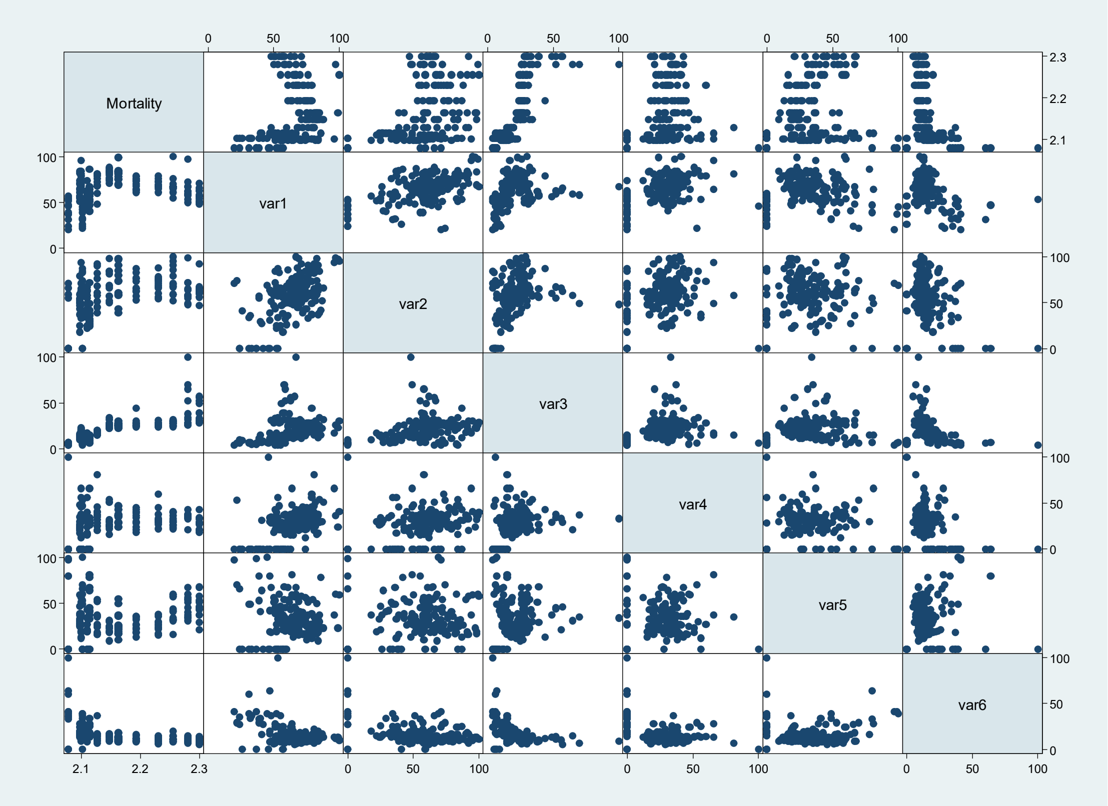

24. Esophageal cancer

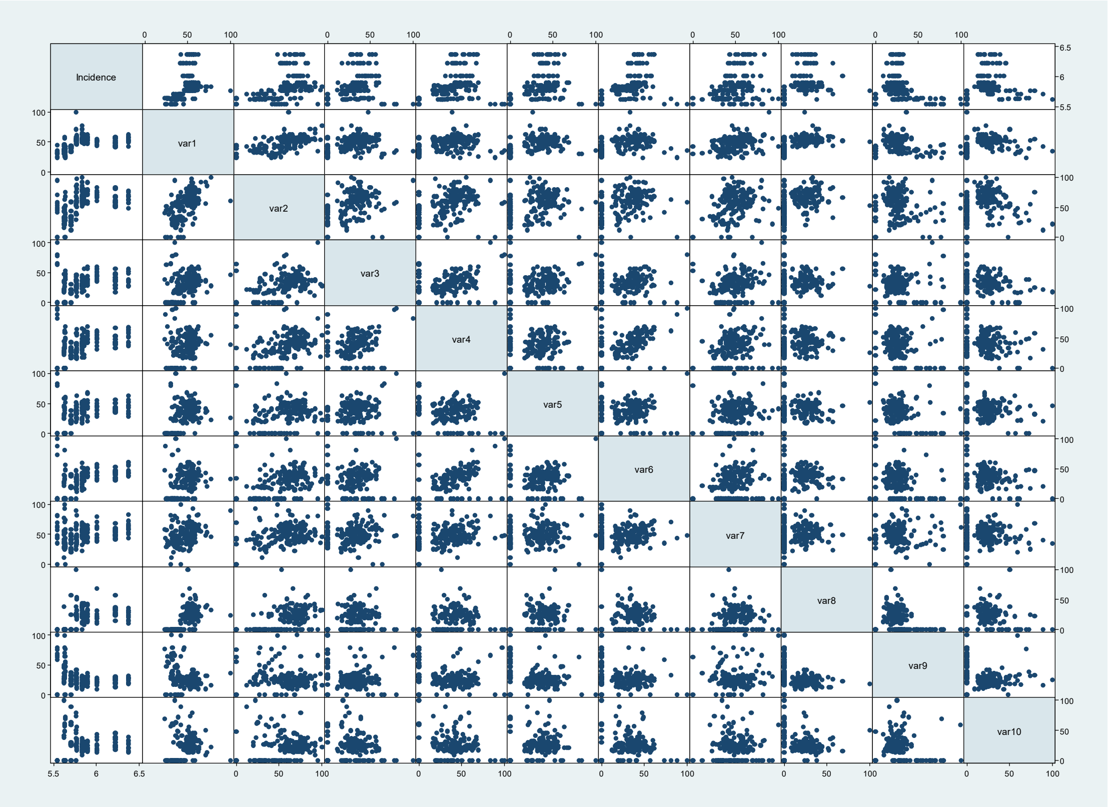

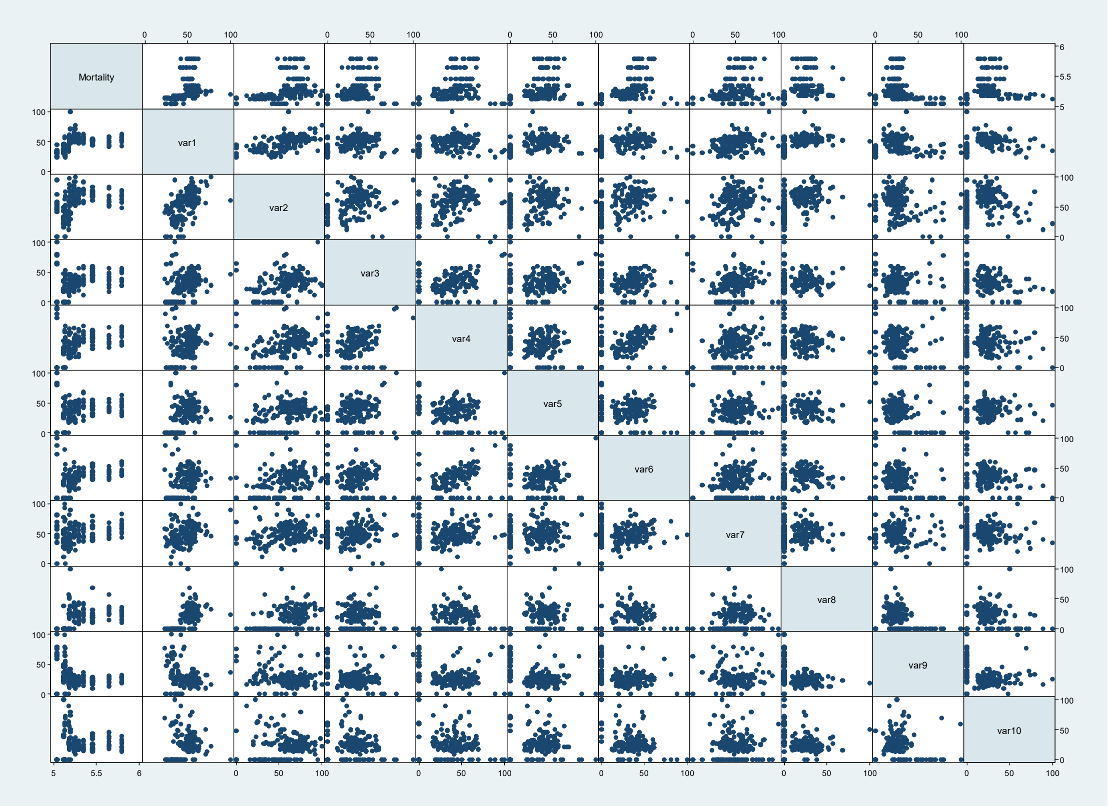

25. Larynx cancer

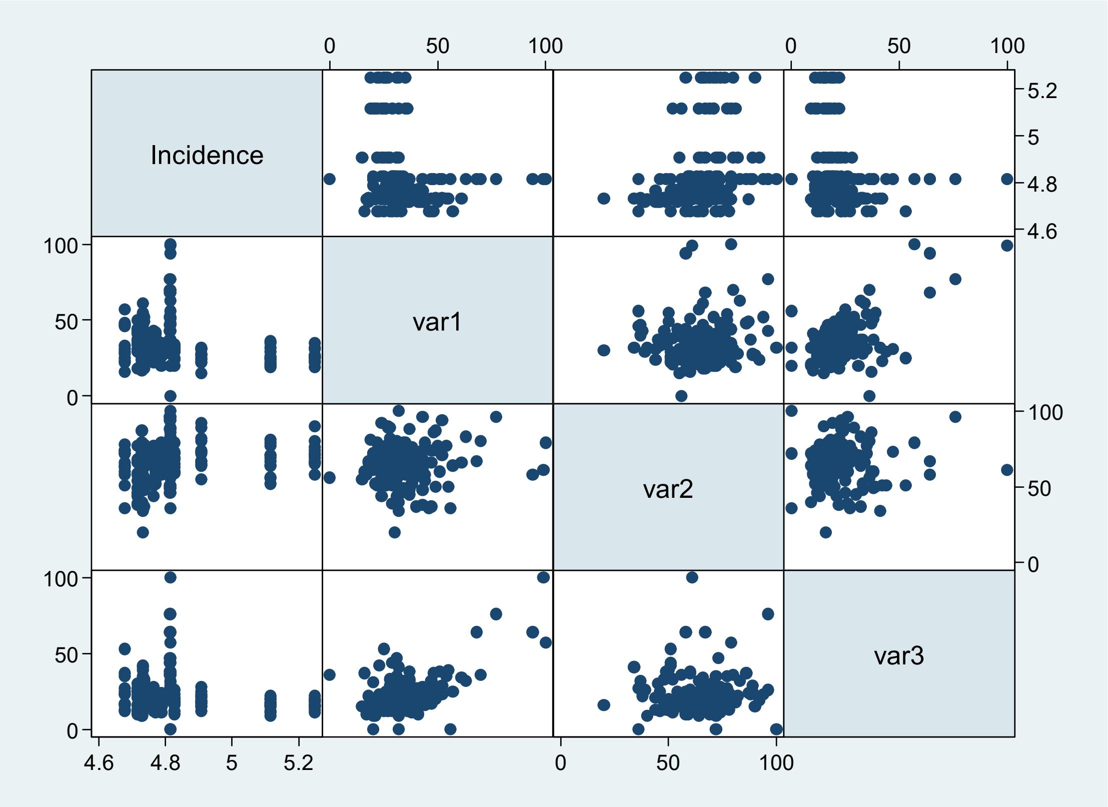

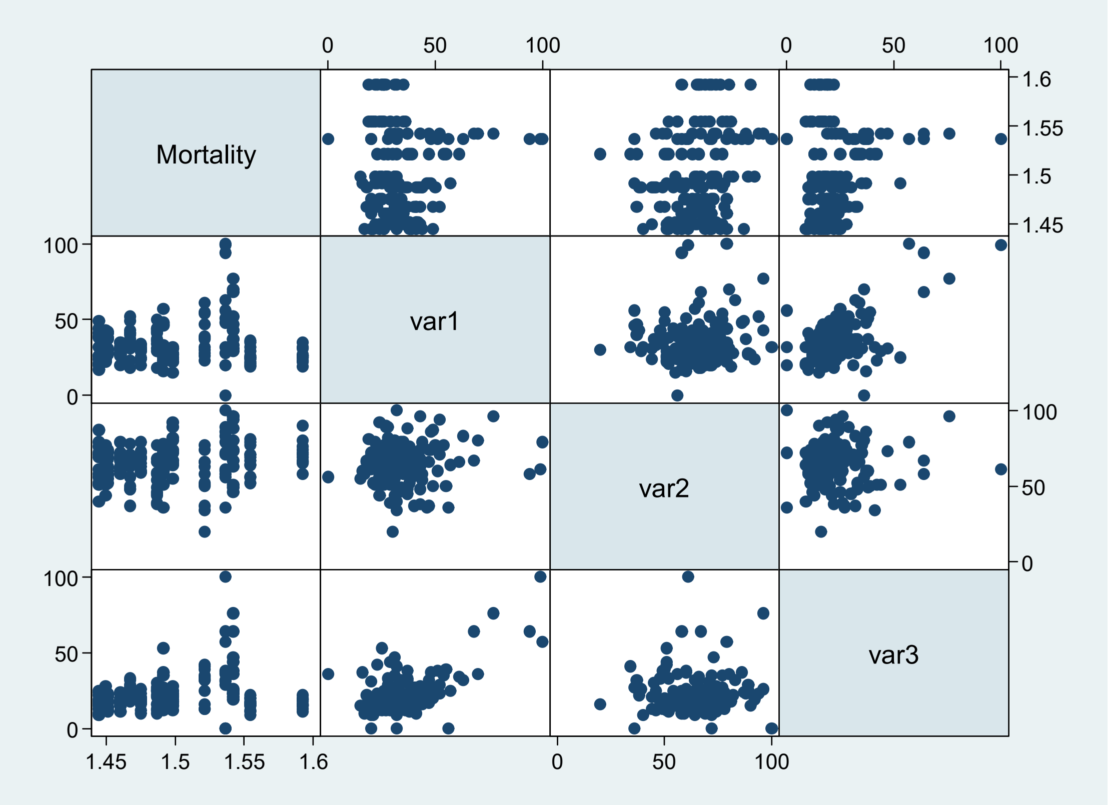

26. Gallbladder and biliary tract cancer

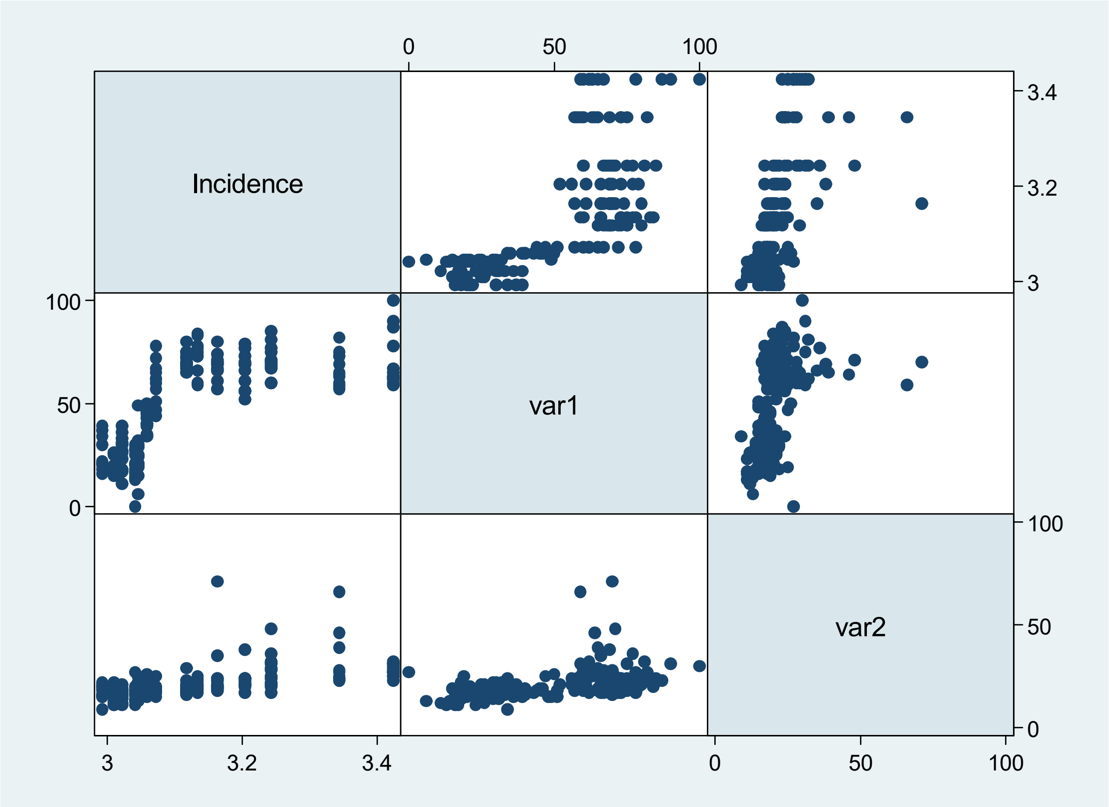

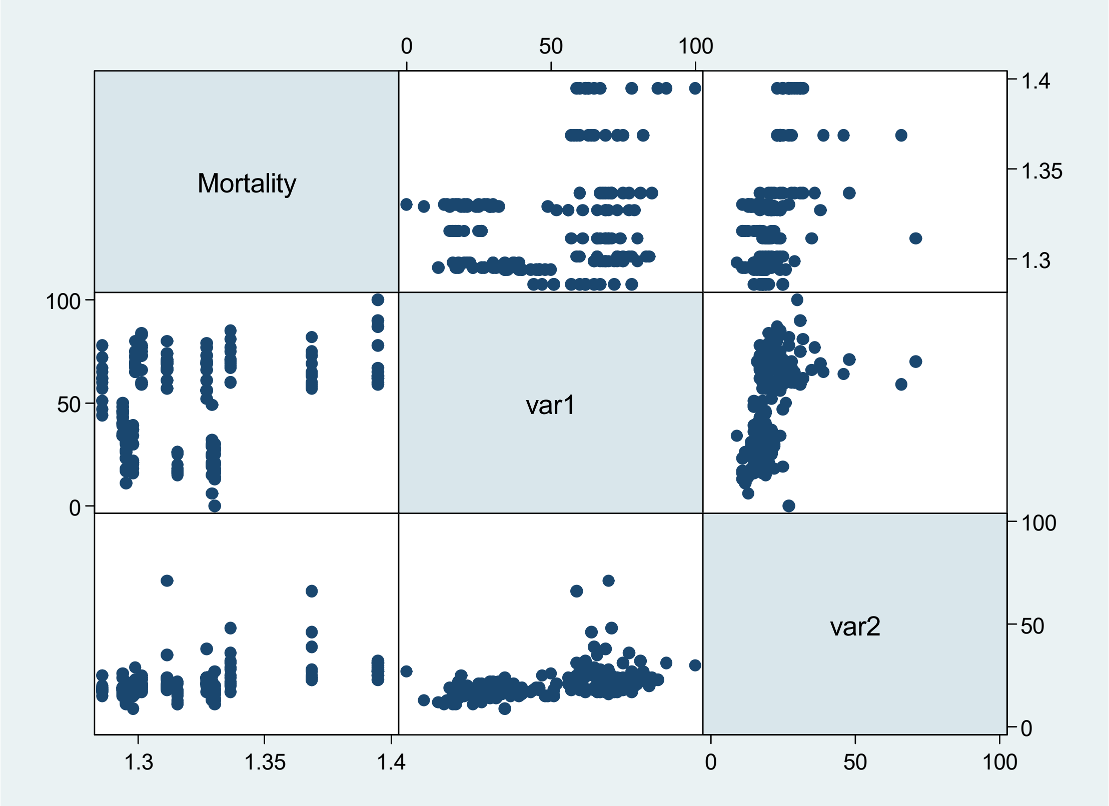

27. Hodgkin lymphoma

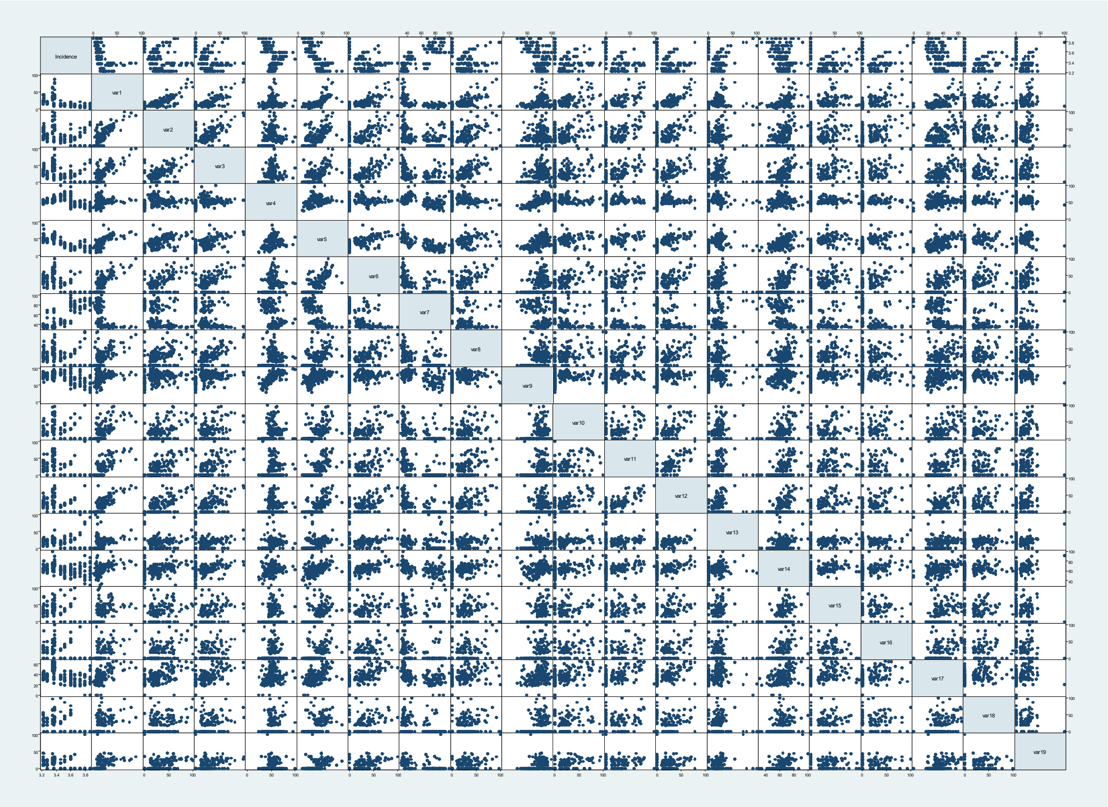

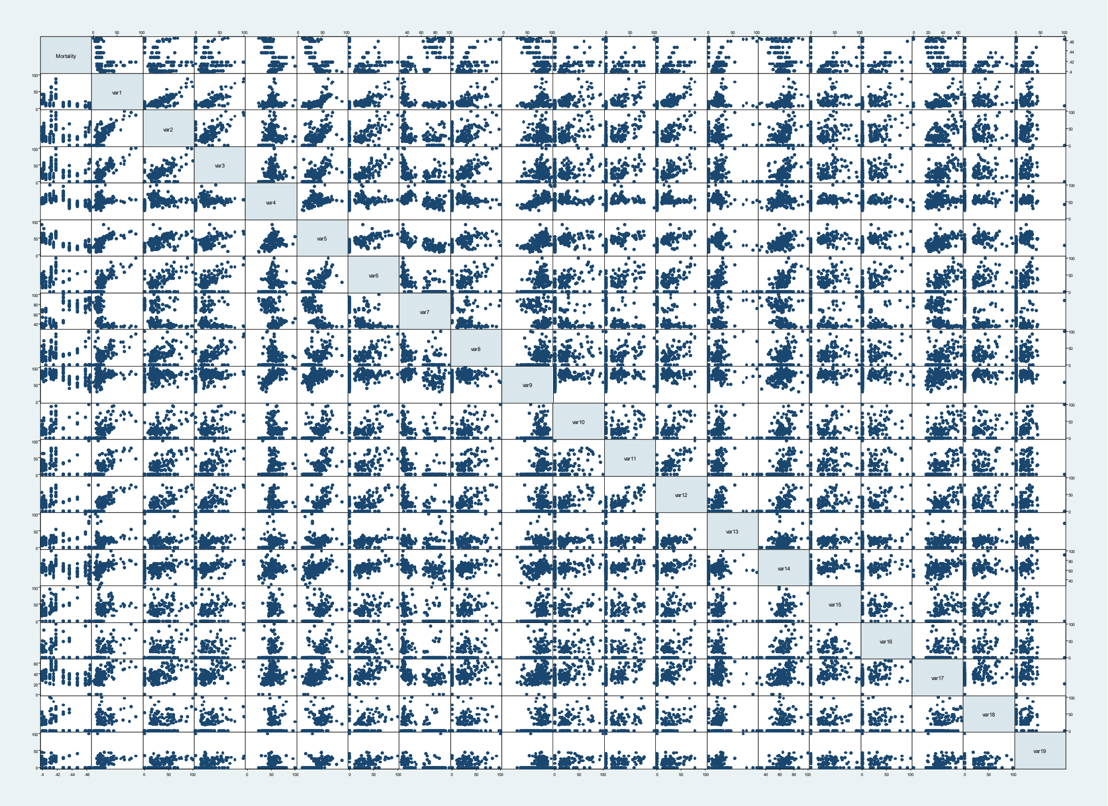

28. Testicular cancer

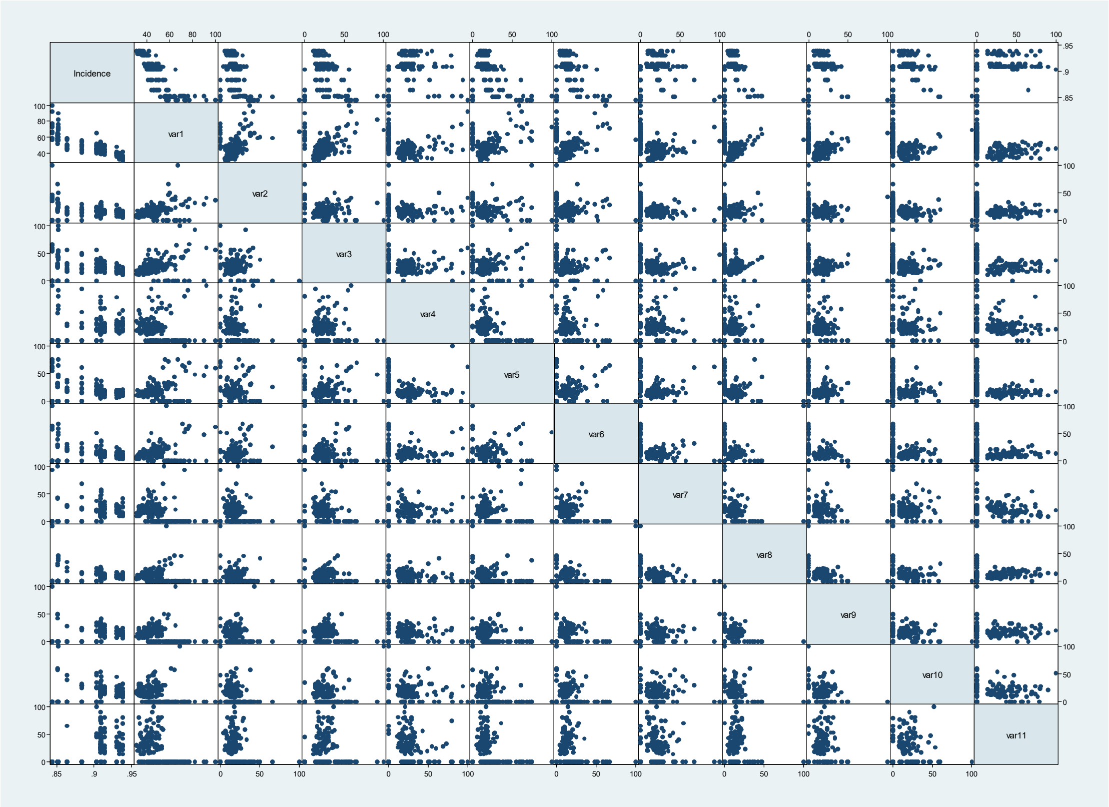

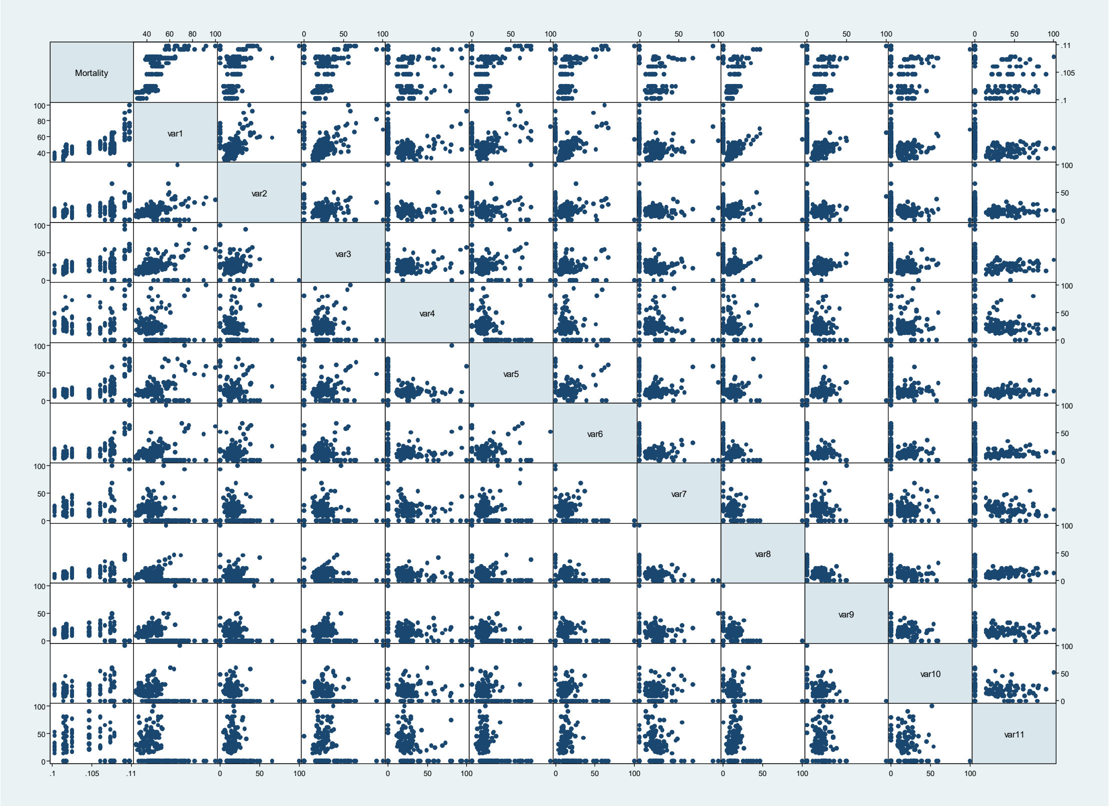

29. Mesothelioma

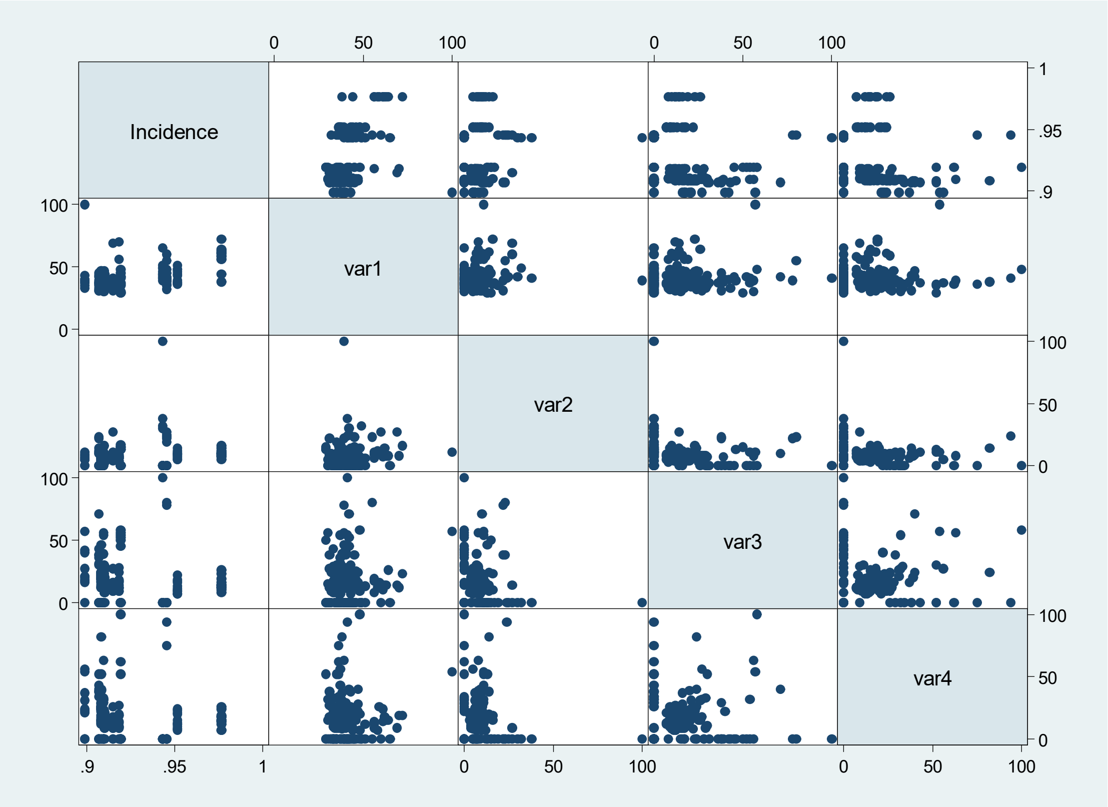

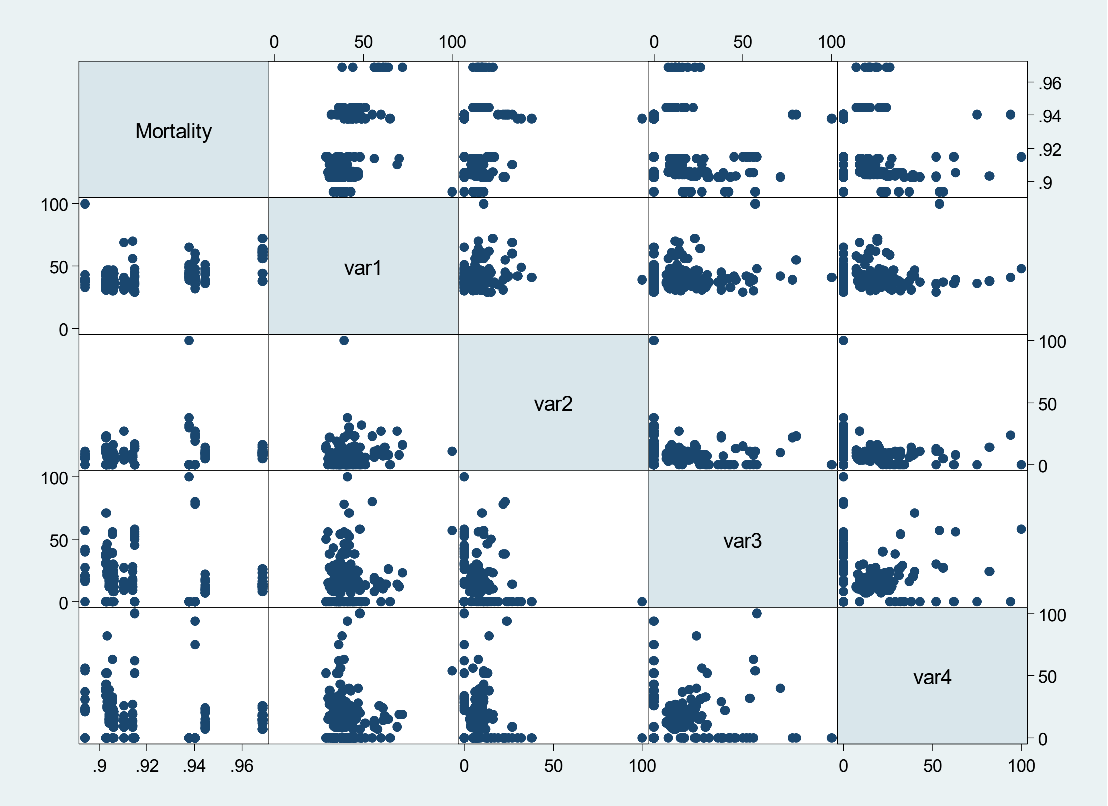

Supplement: Multimedia Appendix 6 [file jmir_v22i11e18998_app6.pdf]
