# Supplementary material for: Leveraging Internet Search Data to Improve the Prediction and Prevention of Noncommunicable Diseases: Retrospective Observational Study
Source: J Med Internet Res. 2020 Nov 12;22(11):e18998. doi: 10.2196/18998 (PMC7691086; doi:10.2196/18998)
Supplement: Multimedia Appendix 7 [file jmir_v22i11e18998_app7.doc]

**Breast cancer**

Incidence=75.4696+0.0014X_1_-0.0063x_2_-0.0099X_3_-0.0101X_4_+0.0118X_5_-0.0110X_6_-0.0085X_7_

Mortality=15.1679-0.0039X_1_-0.0030x_2_-0.0026X_3_-0.0025X_4_+0.0018X_5_-0.0009X_6_-0.0019X_7_

**Colon and rectum cancer**

Incidence=62.1028-0.0266X_1_-0.0408x_2_+0.0088X_3_-0.0155X_4_+0.0139X_5_+0.0058X_6_-0.0363X_7_+0.0201X_8_

Mortality=24.2771-0.0055X_1_-0.0158x_2_-0.0005X_3_+0.0043X_4_+0.0069X_5_-0.0053X_6_-0.0148X_7_+0.0048X_8_

**Lung cancer**

Incidence=71.5506-0.0034X_1_-0.0216X_2_+0.0168X_3_-0.0012X_4_-0.0127X_5_+0.0030X_6_-0.0104X_7_+0.0599X_8_+0.0515X_9_

Mortality=55.2328-0.0097X_1_-0.0311X_2_+0.0170X_3_-0.0027X_4_-0.0203X_5_+0.0008X_6_-0.0090X_7_+0.0412X_8_+0.0705X_9_

**Malignant skin melanoma**

Incidence=20.8237-0.0113X_1_+0.0394X_2_+0.0081X_3_

Mortality=2.6913-0.0013X_1_+0.0051X_2_+0.0009X_3_

**Non-Hodgkin lymphoma**

Incidence=22.0929+0.0136X_1_+0.0038X_2_

Mortality=7.3188+0.0049X_1_+0.0044X_2_

**Leukemia**

Incidence=13.6617+0.0011X_1_+0.0002X_2_+0.0054X_3_+0.0022X_4_

Mortality=8.6796-0.0006X_1_-0.0023X_2_-0.0006X_3_-0.0002X_4_

**Stomach cancer**

Incidence=9.0270+0.0057X_1_+0.0007X_2_+0.0022X_3_-0.0050X_4_

Mortality=5.2682+0.0046X_1_-0.0047X_2_+0.0015X_3_-0.0045X_4_

**Bladder cancer**

Incidence=14.1755-0.0227X_1_+0.0055X_2_+0.0022X_3_-0.0024X_4_+0.0082X_5_-0.0078X_6_+0.0042X_7_-0.0003X_8_+0.0038X_9_-0.0081X_10_+0.0387X_11_-0.0011X_12_

Mortality=5.0721-0.0082X_1_+0.0017X_2_+0.0006X_3_-0.0009X_4_+0.0029X_5_-0.0027X_6_+0.0014X_7_-0.0003X_8_+0.0011X_9_-0.0026X_10_+0.0135X_11_-0.0002X_12_

**Pancreatic cancer**

Incidence=13.7962-0.0088X_1_+0.0113X_2_+0.0251X_3_+0.0073X_4_+0.0127X_5_-0.0076X_6_+0.0022X_7_+0.0105X_8_-0.0045X_9_

Mortality=12.2934-0.0110X_1_+0.0108X_2_+0.0203X_3_+0.0057X_4_+0.0107X_5_-0.0063X_6_+0.0021X_7_+0.0080X_8_-0.0029X_9_

**Uterine cancer**

Incidence=20.0851-0.0419X_1_-0.0295X_2_+0.0244X_3_+0.0317X_4_-0.0197X_5_-0.0133X_6_-0.0114X_7_+0.0037X_8_+0.0378X_9_

Mortality=2.2482-0.0028X_1_-0.0025X_2_+0.0023X_3_+0.0027X_4_-0.0016X_5_-0.0012X_6_-0.0007X_7_-0.0008X_8_+0.0031X_9_

**Kidney cancer**

Incidence=17.1626+0.0148X_1_+0.0146X_2_+0.0047X_3_-0.0023X_4_-0.0008X_5_

Mortality=4.4313+0.0046X_1_+0.0048X_2_+0.0012X_3_-0.0006X_4_-0.0006X_5_

**Brain and nervous system cancer**

Incidence=7.6713+0.0024X_1_+0.0036X_2_-0.0001X_3_-0.0093X_4_-0.0025X_5_+0.0111X_6_

Mortality=4.5623+0.0015X_1_+0.0029X_2_-0.0001X_3_-0.0076X_4_-0.0018X_5_+0.0094X_6_

**Esophageal cancer**

Incidence=5.4182+0.0014X_1_+0.0006X_2_+0.0005X_3_+0.0013X_4_+0.0018X_5_+0.0026X_6_+0.0014X_7_+0.0026X_8_-0.0005X_9_+0.0006X_10_

Mortality=4.9505+0.0002X_1_+0.0003X_2_+0.0007X_3_+0.0011X_4_+0.0016X_5_+0.0027X_6_+0.0015X_7_+0.0022X_8_-0.0003X_9_+0.0004X_10_

**Ovarian cancer**

Incidence=7.0624+0.0060X_1_+0.0010X_2_+0.0014X_3_+0.0034X_4_-0.0026X_5_-0.0035X_6_+0.0007X_7_+0.0014X_8_+0.0018X_9_-0.0016X_10_

Mortality=4.7892+0.0029X_1_+0.0007X_2_+0.0008X_3_+0.0004X_4_-0.0012X_5_-0.0020X_6_+0.0005X_7_+0.0006X_8_+0.0009X_9_-0.0008X_10_

**Multiple myeloma**

Incidence=6.3785+0.0083X_1_+0.0021X_2_+0.0038X_3_-0.0006X_4_-0.0085X_5_-0.0013X_6_+0.0072X_7_+0.0055X_8_

Mortality=3.6522+0.0050X_1_+0.0013X_2_+0.0018X_3_-0.0005X_4_-0.0032X_5_-0.0002X_6_+0.0045X_7_+0.0025X_8_

**Liver cancer**

Incidence=11.4471-0.0474X_1_+0.0189X_2_+0.0113X_3_-0.0006X_4_+0.0044X_5_-0.0002X_6_+0.01111X_7_-0.0069X_8_

Mortality=8.8196-0.0355X_1_+0.0160X_2_+0.0086X_3_-0.0016X_4_+0.0031X_5_-0.0001X_6_+0.0079X_7_-0.0053X_8_

**Lip and oral cavity cancer**

Incidence=6.4793+0.0140X_1_-0.0024X_2_-0.0014X_3_+0.0148X_4_

Mortality=1.5407+0.0036X_1_-0.0005X_2_-0.0004X_3_+0.0030X_4_

**Cervical cancer**

Incidence=6.3747-0.0061X_1_+0.0001X_2_+0.0046X_3_-0.0008X_4_+0.0015X_5_+0.0005X_6_

Mortality=2.1076-0.0009X_1_+0.0006X_2_+0.0038X_3_-0.00004X_4_+0.0004X_5_-0.0010X_6_

**Mesothelioma**

Incidence=0.8859+0.0010X_1_+0.0002X_2_-0.0002X_3_-0.0002X_4_

Mortality=0.8829+0.0010X_1_+0.0002X_2_-0.0002X_3_-0.0002X_4_

**Thyroid cancer**

Incidence=6.4841+0.0043X_1_+0.0034X_2_+0.0060X_3_+0.0038X_4_-0.0036X_5_+0.0005X_6_-0.0009X_7_+0.0024X_8_+0.0034X_9_

Mortality=0.5214+0.0005X_1_+0.0003X_2_+0.0005X_3_+0.0002X_4_-0.0002X_5_-0.0001X_6_-0.0001X_7_+0.0002X_8_+0.0003X_9_

**Hodgkin lymphoma**

Incidence=3.3028+0.0024X_1_+0.0004X_2_-0.0001X_3_+0.0004X_4_-0.0045X_5_-0.0014X_6_+0.0046X_7_+0.0004X_8_-0.0014X_9_+0.0005X_10_+0.0002X_11_-0.0010X_12_+0.0001X_13_+0.0028X_14_-0.0012X_15_-0.0003X_16_+0.0012X_17_-0.0004X_18_-0.0018X_19_

Mortality=0.4063+0.0003X_1_+0.00004X_2_+0.00002X_3_-0.0004X_4_-0.0003X_5_-0.0001X_6_+0.0006X_7_+0.00003X_8_-0.0001X_9_+0.0001X_10_+0.00004X_11_-0.0001X_12_+0.00004X_13_+0.0004X_14_-0.0001X_15_-0.0001X_16_+0.0002X_17_-0.0001X_18_-0.0002X_19_

**Larynx cancer**

Incidence=4.7308-0.0025X_1_+0.0030X_2_-0.0005X_3_

Mortality=1.4516-0.0002X_1_+0.0006X_2_+0.0007X_3_

**Gallbladder and biliary tract cancer**

Incidence=2.8703+0.0031X_1_+0.0051X_2_

Mortality=1.2837+0.0001X_1_+0.0015X_2_

**Testicular cancer**

Incidence=0.9616-0.0018X_1_+0.0001X_2_+0.0001X_3_+0.0003X_4_+0.00004X_5_+0.0001X_6_+0.0001X_7_-0.0001X_8_+0.0002X_9_+0.0001X_10_+0.0003X_11_

Mortality=0.0982+0.0001X_1_+0.00001X_2_+0.00002X_3_-0.00002X_4_+0.00001X_5_+0.000008X_6_-0.000007X_7_+0.00003X_8_+0.00001X_9_+0.000004X_10-_0.00003X_10_

**Ischemic heart disease**

Incidence=310.5169-0.0069X_1_-0.0624X_2_+0.4702X_3_-0.0568X_4_-0.2386X_5_+0.4583X_6_-0.0616X_7_-0.4175X_8_

Incidence=151.8077-0.0464X_1_-0.0392X_2_+0.2517X_3_-0.0361X_4_-0.1439X_5_+0.3279X_6_-0.0536X_7_-0.2316X_8_

**Stroke**

Incidence=173.2640+0.0340X_1_-0.0064X_2_+0.0267X_3_+0.0234X_4_-0.0013X_5_+0.0069X_6_+0.0219X_7_+0.0288X_8_+0.0160X_9_

Mortality=51.8119+0.0092X_1_-0.0321X_2_-0.0106X_3_-0.0416X_4_+0.0358X_5_+0.0050X_6_+0.0107X_7_+0.0055X_8_-0.0004X_9_

**Atrial fibrillation and flutter**

Incidence=101.2412+0.0281X_1_+0.1583X_2_+0.0171X_3_-0.0127X_4_-0.0065X_5_+0.0506X_6_+0.0130X_7_+0.0029X_8_+0.0014X_9_-0.0221X_10_+0.0030X_11_-0.0035X_12_+0.0160X_13_+0.0095X_14_-0.0285X_15_+0.1864X_16_-0.0066X_17_+0.0215X_18_

Mortality=7.3492+0.0043X_1_+0.0091X_2_+0.0015X_3_-0.0013X_4_-0.0007X_5_+0.0055X_6_+0.0010X_7_-0.00001X_8_+0.0001X_9_-0.0026X_10_-0.0002X_11_-0.0005X_12_+0.0013X_13_+0.0008X_14_-0.0020X_15_+0.0150X_16_-0.0005X_17_+0.0017X_18_

**Cardiomyopathy**

Incidence=19.8478+0.0036X_1_+0.0035X_2_+0.0127X_3_

Mortality=8.4928+0.0031X_1_+0.0101X_2_+0.0046X_3_

**Diabetes mellitus**

Incidence=372.9845+0.1916X_1_-0.6313X_2_-0.3292X_3_+1.7090X_4_+0.6203X_5_-0.8534X_6_-0.2871X_7_

Mortality=24.3108-0.0020X_1_+0.0077X_2_+0.0076X_3_-0.0529X_4_-0.0066X_5_+0.0023X_6_-0.0007X7
