# Supplementary material for: Leveraging Internet Search Data to Improve the Prediction and Prevention of Noncommunicable Diseases: Retrospective Observational Study
Source: J Med Internet Res. 2020 Nov 12;22(11):e18998. doi: 10.2196/18998 (PMC7691086; doi:10.2196/18998)
Supplement: Multimedia Appendix 8 [file jmir_v22i11e18998_app8.doc]

| **Evaluation results of prediction models for all the chronic diseases** | | | | | | | |
| --- | --- | --- | --- | --- | --- | --- | --- |
|  |  | **Incidence** | | | **Mortality** | | |
|  |  | **R²** | **Adj R²** | **RMSE** | **R²** | **Adj R²** | **RMSE** |
| 1 | Diabetes mellitus | 80% | 80% | 20.54 | 82% | 81% | 0.59 |
| 2 | Ischemic heart disease | 69% | 67% | 9.50 | 77% | 76% | 4.86 |
| 3 | Stroke | 88% | 87% | 1.43 | 62% | 60% | 1.15 |
| 4 | Atrial fibrillation and flutter | 96% | 95% | 1.91 | 94% | 94% | 0.17 |
| 5 | Breast cancer | 12% | 8% | 1.28 | 44% | 41% | 0.25 |
| 6 | Lung cancer | 65% | 62% | 0.85 | 72% | 71% | 0.83 |
| 7 | Colon and rectum cancer | 42% | 40% | 1.21 | 52% | 49% | 0.44 |
| 8 | Malignant skin melanoma | 47% | 46% | 0.73 | 43% | 42% | 0.1 |
| 9 | Non-Hodgkin lymphoma | 39% | 39% | 0.34 | 27% | 26% | 0.18 |
| 10 | Uterine cancer | 49% | 46% | 1.61 | 43% | 39% | 0.14 |
| 11 | Cardiomyopathy and myocarditis | 20% | 19% | 0.32 | 21% | 19% | 0.32 |
| 12 | Kidney cancer | 61% | 60% | 0.46 | 61% | 60% | 0.14 |
| 13 | Pancreatic cancer | 70% | 69% | 0.59 | 69% | 67% | 0.5 |
| 14 | Bladder cancer | 60% | 57% | 0.48 | 61% | 57% | 0.16 |
| 15 | Leukemia | 25% | 23% | 0.26 | 16% | 14% | 0.12 |
| 16 | Liver cancer | 71% | 69% | 0.66 | 68% | 66% | 0.54 |
| 17 | Stomach cancer | 31% | 29% | 0.16 | 69% | 69% | 0.1 |
| 18 | Lip and oral cavity cancer | 63% | 62% | 0.26 | 57% | 56% | 0.06 |
| 19 | Brain and nervous system cancer | 38% | 36% | 0.25 | 37% | 34% | 0.20 |
| 20 | Thyroid cancer | 68% | 67% | 0.24 | 67% | 65% | 0.02 |
| 21 | Multiple myeloma | 58% | 56% | 0.26 | 50% | 48% | 0.14 |
| 22 | Ovarian cancer | 63% | 61% | 0.17 | 45% | 41% | 0.1 |
| 23 | Cervical cancer | 50% | 48% | 0.11 | 68% | 66% | 0.04 |
| 24 | Esophageal cancer | 44% | 41% | 0.17 | 38% | 34% | 0.17 |
| 25 | Larynx cancer | 12% | 10% | 0.15 | 6% | 4% | 0.04 |
| 26 | Gallbladder and biliary tract cancer | 57% | 57% | 0.08 | 19% | 18% | 0.03 |
| 27 | Hodgkin lymphoma | 80% | 77% | 0.10 | 78% | 75% | 0.01 |
| 28 | Testicular cancer | 78% | 77% | 0.01 | 62% | 60% | 0.00 |
| 29 | Mesothelioma | 28% | 26% | 0.02 | 28% | 26% | 0.02 |
